# Condition-specific mediolateral sway and task-dependent postural complexity in individuals with generalized joint hypermobility

**DOI:** 10.3389/fbioe.2026.1911414

**Published:** 2026-09-21

**Authors:** Luqi Guo, Ye Luo, Linguang Feng, Xiaowen Chen, Qiuyang Xiao, Fei Tian, Shaobai Wang

**Affiliations:** 1 Key Laboratory of Exercise and Health Sciences of the Ministry of Education, Shanghai University of Sport, Shanghai, China; 2 Department of Rehabilitation Medicine, Changzhi Medical College, Changzhi, Shanxi, China

**Keywords:** balance, center of pressure, generalized joint hypermobility, multiscale entropy, postural control

## Abstract

**Objective:**

Generalized joint hypermobility (GJH) features ligamentous laxity and proprioceptive deficits, which impair balance. Traditional center of pressure (COP) metrics may not fully elucidate postural instability mechanisms. Multiscale entropy (MSE) evaluates nonlinear time series complexity across scales. This study integrated linear COP and MSE metrics across task conditions to evaluate GJH static postural control and compensatory strategies.

**Methods:**

Twenty-eight GJH and twenty-six healthy controls (all right-limb dominant) were recruited. COP data were recorded using a Kistler 3D force plate. Each participant performed double-leg, left-leg, and right-leg stances under eyes-open and eyes-closed conditions. Pairwise comparisons targeted the eyes-closed tasks. Mixed-design ANOVA examined the effects of group and condition on balance parameters (α = 0.05). Spearman correlations between COP and MSE metrics were computed for each group across all six conditions (FDR-corrected); between-group differences in correlation strength were further tested via Fisher’s r-to-z transformation. Trial-to-trial reliability was assessed via ICC (2,1).

**Results:**

For COP metrics, ANOVA revealed significant task main effects across most parameters (P < 0.01, except LFS). Except for AP_SI (P = 0.04), no other significant main effects of group were observed (P > 0.05). Group × task interactions were not significant. Pairwise comparisons indicated that under the LEC, the GJH group showed significantly greater AP_SI and ML_Amp (FDRq <0.05) and smaller ML_SI (FDRq <0.05). For MSE metrics, significant group × task interactions (P < 0.05), group main effects and task main effects (P < 0.01) were observed across most parameters. The GJH displayed significantly higher MSE during bipedal and right-limb tasks (P < 0.05). However, these between-group differences were a descriptive pattern of attenuation with increasing task difficulty. Twenty-nine COP-MSE correlations reached FDR significance in the GJH group, versus none in healthy controls. However, direct between-group comparisons using Fisher’s r-to-z transformation, FDR-corrected across all 204 COP-MSE pairs, did not identify a significant difference in correlation strength.

**Conclusion:**

Individuals with young GJH showed localized, condition-specific differences in mediolateral sway during LEC and a descriptive pattern of task-dependent attenuation in postural complexity as demands increased. Combining COP parameters with MSE analysis provided complementary insights that could be captured by neither measure alone.

## 1 Introduction

Generalized joint hypermobility (GJH) is a hallmark of heritable connective tissue disorders, characterized by a range of motion (ROM) in multiple joints that exceeds normal physiological limits (Hakim and Grahame, 2003). The prevalence of GJH in adults ranges from 10% to 25%, with females being five times more likely to be affected than males (Remvig et al., 2007). Evidence suggests that individuals with this trait often present with multisystemic involvements, including the musculoskeletal, gastrointestinal, and cardiovascular systems, which remarkably affect long-term health and quality of life.

Postural stability relies on the central nervous system’s integration of various sensory inputs and the precise coordination of motor effectors (Horak, 2006). In individuals with GJH, researchers show that ligamentous and connective tissue laxity reduce the sensitivity of joint mechanoreceptors, potentially impairing proprioceptive function and compromising afferent signal reliability (Bates et al., 2021). As a result, the neuromuscular system is compelled to increase active muscular engagement to compensate for reduced passive joint stability (Simmonds and Keer, 2007). Task constraints, such as visual deprivation or unilateral stance on nondominant limbs (Promsri et al., 2018), escalate sensory and motor demands. When these sensory and biomechanical challenges interact with GJH-related joint laxity, the postural control system needs to continuously reweight sensory inputs and adapt its control strategies to prevent instability. Such compensatory adjustments are hypothesized to interfere with postural control strategies and ultimately affect balance function. Limb dominance is another important factor in postural control. The dominant limb demonstrates superior neuromuscular coordination and greater motor control precision during single-leg stance compared with the nondominant limb (Promsri et al., 2018). In individuals with GJH where proprioceptive reliability is already compromised, this asymmetry may be further amplified, potentially reducing the nondominant limb’s capacity to compensate for joint laxity. When visual input is additionally removed, the postural system may be simultaneously deprived of its primary compensatory sensory channel and the motor advantage of the dominant limb. Given that balance is a critical function for performing activities of daily living, its quantification represents a milestone in planning appropriate rehabilitation interventions (Rigoldi et al., 2012).

Static posturography is one of the most widely utilized techniques for assessing human balance. Force platforms are typically employed to record the drift of the center of pressure (COP) and investigate displacement variations. The range of COP displacement during quiet standing serves as a classic indicator of postural stability, where large displacements are generally associated with diminished stability (Prieto et al., 1996). While these traditional approaches address the magnitude of variability, they often rely on the assumption of stationarity and randomness, thereby overlooking the temporal structure of COP fluctuations (Croft et al., 2008). Linear metrics quantify only the spatial magnitude or overall dispersion of sway but cannot differentiate between distinct underlying control strategies, as individuals with identical sway amplitudes may employ fundamentally different dynamic control mechanisms (Busa and Richard, 2016). The lack of analysis regarding the dynamical characteristics of COP displacement limits a deep understanding of the mechanisms underlying postural instability. Furthermore, these linear methods fail to account for the temporal variations arising from complex sensorimotor integration (Madeleine et al., 2010).

Researchers have adopted novel analytical frameworks to address this gap in data analysis and identification. Entropy, a nonlinear dynamical parameter applied to feature extraction, is a physical measure of system complexity widely used in computational complexity analysis (Busa and Richard, 2016). Established by Richman and Moorman (Richman and Moorman, 2000), sample entropy (SampEn) measures time-series regularity by excluding self-matches; low values indicate regular signals, and high values reflect greater irregularity. Originally proposed by Costa et al. (2005), multiscale entropy (MSE) incorporates the advantages of SampEn while characterizing the complexity of nonlinear time series across multiple scales (Costs et al., 2002). MSE quantifies the intrinsic complexity of single- and multichannel signals (Costa et al., 2005) and provides meaningful measures of regularity for biological signals, such as COP measurements (Kafantaris et al., 2023). In their foundational work, healthy biological systems tend to exhibit higher MSE values across coarser scales compared with pathological or aging-related states, reflecting their greater adaptive complexity and biological functional capacity (Manor et al., 2010). Conversely, reduced MSE indicates a regular, stereotyped signal, reflecting diminished system flexibility and adaptability. However, this framework was established primarily in the context of age- or disease-related degradation of system complexity. An elevated entropy does not inherently indicate adaptive postural control; high MSE values may also reflect increased signal irregularity or noise. Therefore, the interpretation of MSE in the context of postural control must be considered in relation to task demands and group-specific mechanisms. Stergiou et al. (2006) proposed a framework of optimal movement variability positing that healthy biological systems operate at an intermediate level of complexity: neither too rigid (overly regular, low entropy) nor too random (chaotic, excessively high entropy). Deviations in either direction may reflect maladaptive control strategies.

The interpretation of MSE changes in postural control requires a multifaceted, bidirectional perspective rather than a simplified one. Low MSE values (reflecting increased regularity) may indicate rigid mechanical stiffening, joint locking (such as knee hyperextension), or overly constrained control strategies used to freeze degrees of freedom against joint laxity. Elevated MSE values do not inherently denote successful adaptive compensation; excessively high entropy may also signify a loss of deterministic neuromuscular coordination, resulting in uncorrelated, noisy postural output due to impaired proprioceptive feedback or high sensory challenge (Busa and Richard, 2016). Therefore, MSE dynamics must be interpreted in close conjunction with linear sway magnitudes, mechanical joint constraints (such as knee alignment), and task-dependent postural demands.

The primary objective of this study was to evaluate the balance characteristics of the GJH population by integrating traditional linear measures with nonlinear multiscale entropy analysis across tasks of varying difficulty. This study hypothesizes that: 1) compared with healthy controls, the GJH group would exhibit greater postural sway under high-difficulty conditions, particularly during nondominant-limb eyes-closed stance; and 2) the MSE values in the GJH group would be higher during lower-demand tasks, reflecting greater COP signal complexity, and this complexity advantage would diminish progressively as task demands increased, with the most constrained condition potentially revealing a point of attenuation.

## 2 Methods

### 2.1 Participants

This study was approved by the Ethics Committee of the Shanghai University of Sport (China) (Ethics Approval No.102772025RT171). Sample size was estimated using G*Power 3.1.9.7. Prior work applying sample entropy to postural sway in a comparable hypermobility population (Ehlers-Danlos syndrome hypermobility type) reported significantly lower AP and ML entropy values in the hypermobile group relative to controls, reflecting a loss of signal complexity and increasingly ineffective postural control strategies (Rigoldi et al., 2012), consistent with medium-to-large between-group effects. A conservative effect size (partial η^2^ = 0.10) was assumed for the present power calculation, yielding a minimum of 22 participants per group (total N = 44) for a repeated-measures ANOVA with six measurements, a correlation among repeated measures of 0.5, two-tailed α = 0.05, and power = 0.80. This calculation was designed to detect the primary Group × Task interaction for linear COP outcomes and does not account for the higher dimensionality of the MSE analysis (two directions × eight scales × six tasks) or the exploratory correlation analyses. Findings from scale-specific MSE comparisons and correlation analyses should therefore be interpreted with the possibility of limited statistical power in mind. Among the 54 participants recruited, 28 individuals were in the GJH group and 26 in the healthy control group. Prior to the commencement of the experiment, all participants read and provided written informed consent. All subjects were screened and recruited by professional physical therapists. GJH diagnosis was based on the revised Beighton criteria (Juul-Kristensen et al., 2017). The inclusion criteria for the GJH group were a Beighton score of ≥5 and the presence of hyperextension in at least one knee joint. The Beighton score alone does not require lower-limb joint involvement. In addition to the Beighton score, lower limb joint laxity was further assessed using the lower limb assessment score (LLAS) (Ferrari et al., 2005), with a unilateral score of ≥7 out of 12 indicating the presence of lower limb joint laxity. Healthy controls were included if they had a Beighton score of ≤2, exhibited no knee hyperextension, and showed no lower limb joint laxity as assessed using LLAS (<7). Exclusion criteria for both groups included Marfan syndrome, Ehlers–Danlos syndrome, a history of severe lower limb structural trauma, and previous lower limb surgery. The right leg was identified as the dominant limb for all participants. Leg dominance was determined by asking participants which leg they preferred to use to kick a ball. Basic information of the subjects is presented in Table 1.

**TABLE 1:** Basic information of subjects.

Group	GJH	Healthy	P
Number	28 (13M,15F)	26 (13M,13F)	>0.05
Age	20.41 ± 1.95	20.76 ± 2.07	>0.05
Height (cm)	169.02 ± 8.63	170.05 ± 9.40	>0.05
Weight (kg)	63.34 ± 13.56	65.19 ± 13.05	>0.05
LLAS (L/R) (score)	L (8.55 ± 1.06), R (8.59 ± 1.01)	L (2.50 ± 1.10), R (2.82 ± 1.18)	<0.001
Beighton score (score)	7.11 ± 1.02	1.08 ± 0.85	<0.001
R_KNEE_	3	0	_
L_KNEE_	1	0	_
D_KNEE_	24	0	_

Abbreviations: Male(M), Female(F), R_KNEE_ (Right knee hyperextension), L_KNEE_ (Left knee hyperextension), D_KNEE_ (Double knee hyperextension), LLAS (Lower Limb Assessment Score).

### 2.2 Beighton score assessment and lower limb assessment score

First, GJH was evaluated using the 9-point Beighton score system, which consists of five standardized clinical maneuvers: (1) passive dorsiflexion of the fifth metacarpophalangeal joint >90° (bilateral, 1 point each); (2) passive apposition of the thumb to the flexor aspect of the forearm (bilateral, 1 point each); (3) hyperextension of the elbow joint >10° (bilateral, 1 point each); (4) hyperextension of the knee joint >10° (bilateral, 1 point each); and (5) trunk flexion with knees fully extended, placing palms flat on the floor (1 point).

Second, lower limb joint laxity was further quantified using LLAS, evaluating 12 standardized items per limb (24 items in total). The LLAS protocol systematically assessed multijoint ROM and ligamentous laxity across the lower extremity using specific cut-off thresholds: hip flexion (>90°), hip abduction (>50°), knee hyperextension (heel-to-bed distance >3 cm), knee anterior drawer test, tibial internal/external rotation (>2 cm displacement at 90° hip and knee flexion), ankle dorsiflexion (>15° at 45° knee flexion), ankle anterior drawer test, subtalar inversion (>45°), midtarsal joint inversion (>45°), forefoot mobility (dorsiflexion/plantarflexion/abduction/adduction >1 cm), hallux dorsiflexion (>90° relative to the metatarsal), and medial longitudinal arch flexibility (assessed weight-bearing with foot inversion-relaxation). Each item was scored binarily (0 = normal, 1 = hypermobile), yielding a total score ranging from 0 to 24, with higher scores indicating greater lower limb laxity.

For rigorous methodological standardization, all passive angular measurements for Beighton and LLAS protocols were precisely quantified using a standard handheld goniometer. All assessments were performed by a single licensed physical therapist (with 5 years of clinical musculoskeletal experience) who had completed formal training in standardized hypermobility testing procedures prior to data collection. The evaluator was strictly blinded to the study hypotheses, experimental conditions, and participant group assignments to minimize observer bias. Furthermore, all evaluations were conducted in a single session per participant to eliminate interrater variability.

### 2.3 Data acquisition and experimental tasks

Postural COP data were acquired using a Kistler 3D force platform (Model 9286BA; Kistler Corporation, Winterthur, Switzerland) with dimensions of 600 × 400 × 35 mm (length × width × height). The platform was positioned on a level laboratory floor and calibrated to zero before each task. COP displacement was recorded in the antero-posterior (AP) and medio-lateral (ML) directions at a sampling frequency of 1000 Hz. The force platform was interfaced with a computer via a charge amplifier, and BioWare software (v5.2.3.9) was utilized for signal acquisition and storage. Each participant was required to perform six randomized static standing tasks, crossing three postural conditions (double-leg, left-leg, and right-leg stance) with two visual conditions (eyes-open and eyes-closed). The tasks were performed with the following (Figure 1): 1) double-leg stance with eyes open (DEO); 2) double-leg stance with eyes closed (DEC); 3) right-leg stance with eyes open (REO); 4) right-leg stance with eyes closed (REC); 5) left-leg stance with eyes open (LEO); and 6) left-leg stance with eyes closed (LEC). The order in which the experimental tasks were performed was randomized across participants. Following standard guidelines (Ruhe et al., 2010), three 30-s trials were recorded per condition and visually screened for artifacts. All 54 participants completed all six conditions. Linear and MSE metrics were computed independently for each individual trial, and the resulting values were averaged across all valid trials for that participant and condition (up to three) to yield a single representative value per condition for each participant; trials failing quality control (e.g., due to recording artifacts, or extreme outlier values) were excluded prior to averaging, such that a small number of participant–condition combinations were based on fewer than three trials.

**FIGURE 1:**
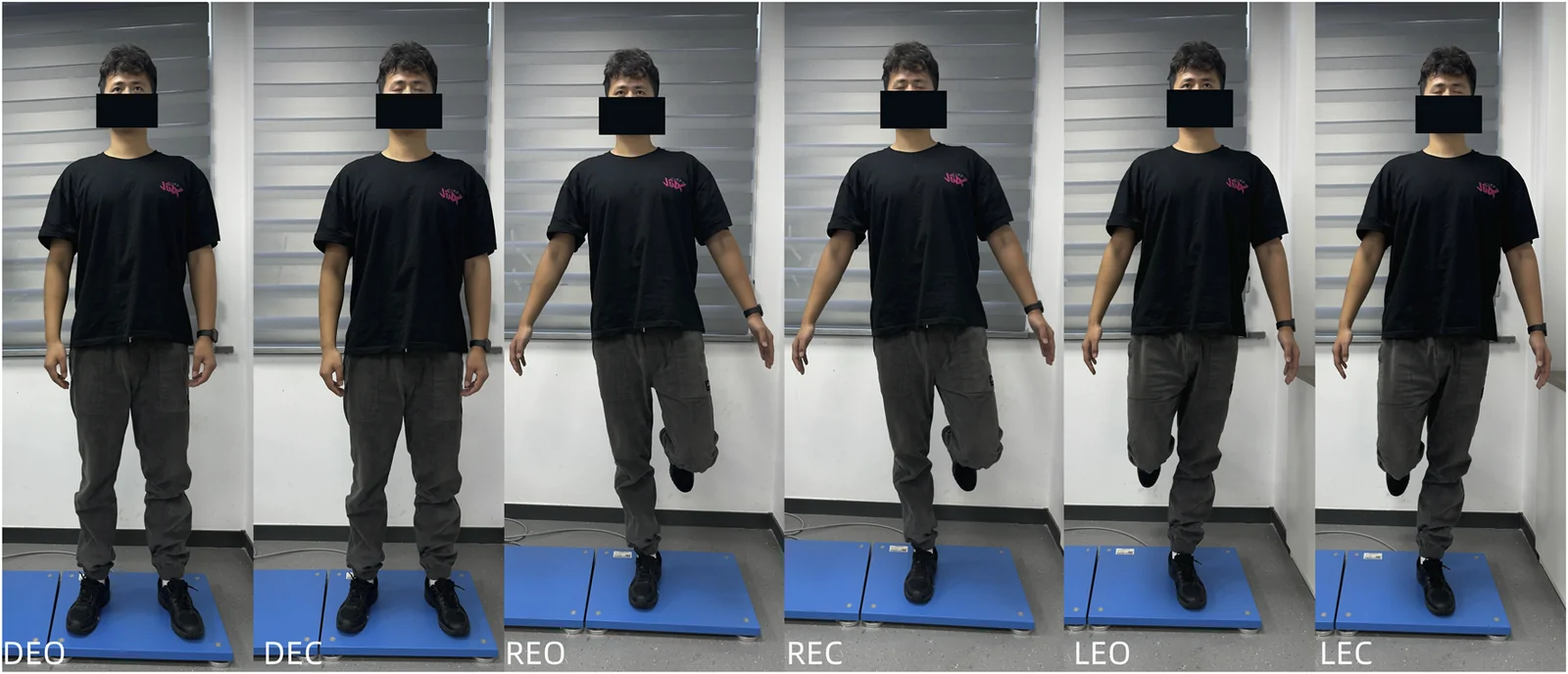
Schematic diagram of the six standing tasks. Abbreviations: Double-leg stance with eyes open (DEO); Double-leg stance with eyes closed (DEC); Right-leg stance with eyes open (REO); Right-leg stance with eyes closed (REC); Left-leg stance with eyes open (LEO); Left-leg stance with eyes closed (LEC).

### 2.4 Data collection procedure

During testing, the participants were instructed to stand with their arms positioned naturally at their sides. All participants wore standardized socks and sneakers provided by the laboratory throughout the testing session (Figure 2). Foot placement was standardized for all stance conditions. During double-leg stance trials, the participants were instructed to stand with their feet approximately hip-width apart, with toes pointed outward at 10°–15°. During single-leg stance trials, the participants were instructed to position the supporting foot at the center of the force platform, with the toe pointed outward at 0°–5°. In accordance with the standardized testing protocol, the participants stood at the center of the force platform. All participants received identical verbal instructions and were required to maintain the same standardized posture throughout testing. Prior to formal data collection, the participants completed one familiarization trial for each of the six standing tasks to minimize learning effects.

**FIGURE 2:**
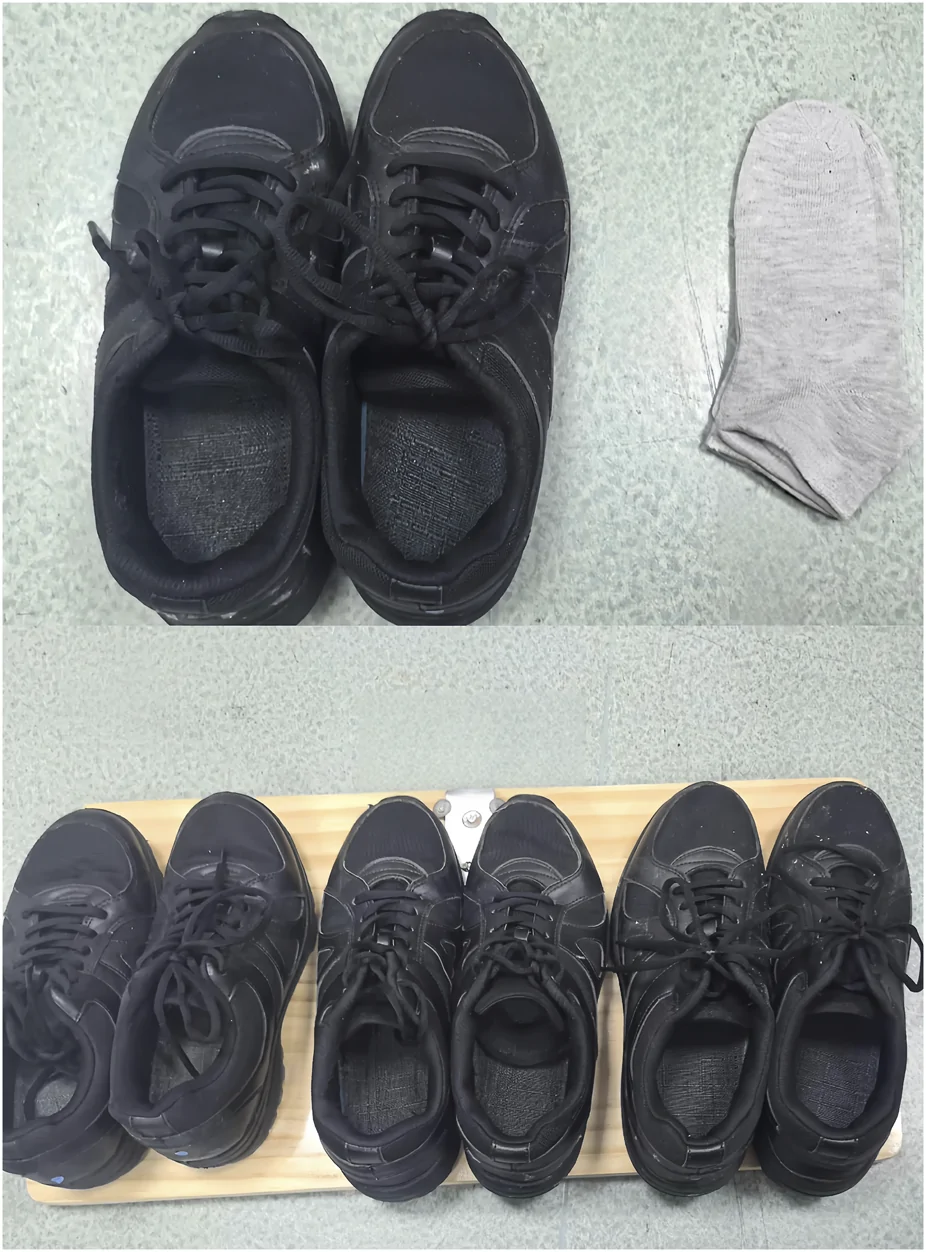
Uniform sneakers and socks for the Lab.

During single-leg stance trials, contact between the nonsupporting and supporting limbs was strictly prohibited. For double-leg stance trials, the participants were instructed to “remain as still and stable as possible.” Throughout all standing tasks, they were instructed to maintain a relaxed, natural knee position. Active knee locking was discouraged; however, no fixed knee flexion angle was imposed. Knee posture was monitored visually by a trained investigator throughout each trial to ensure compliance. In the single-leg stance tasks, if the nonsupporting foot touched the ground, then the trial was considered invalid and was repeated until a successful 30-s recording was obtained.

During eyes-open conditions, the participants were instructed to look straight ahead on the wall at eye level. A quiet environment was maintained throughout the entire procedure to ensure an optimal testing condition for all participants. A 30-s rest period (seated) was provided between consecutive trials to minimize the effects of physical fatigue.

### 2.5 Data processing

Time-series data of COP_x_ and COP_y_ were exported using BioWare software (v5.2.3.9) and subsequently processed in MATLAB R2023a (MathWorks, Inc., Natick, MA, USA). All entropy calculations were implemented using a custom in-house MATLAB (R2023a) script rather than a published toolbox, following the vectorized template-matching procedure described above (Chebyshev distance, no self-matches). First, the raw COP trajectories were downsampled to 100 Hz, after which a 4th-order zero-phase Butterworth low-pass filter with a cutoff frequency of 10 Hz was applied to eliminate high-frequency noise (Ruhe et al., 2010). The 10-Hz cutoff was selected to remove measurement noise and high-frequency mechanical artifacts while preserving the physiologically relevant frequency content of COP sway during quiet standing, which is concentrated below several hertz (Ruh et al., 2010); This cutoff also satisfies the recommended sampling-to-cutoff ratio for reliable MSE estimation at the analyzed scales (Gow et al., 2015). The same filter and downsampling procedure were applied identically to every trial, for both linear and nonlinear analyses, prior to any entropy calculation.No additional normalization or detrending was applied beyond this filtering and downsampling step. From the filtered data, the following traditional linear balance parameters were calculated: total path length (TPL), antero-posterior path length (AP_PL), medio-lateral path length (ML_PL), antero-posterior displacement amplitude (AP_Amp), medio-lateral displacement amplitude (ML_Amp), antero-posterior sway index (AP_SI), medio-lateral sway index (ML_SI), antero-posterior displacement standard deviation (AP_SD), medio-lateral displacement standard deviation (ML_SD), total mean velocity (MV), antero-posterior velocity standard deviation (AP_Vel_SD), medio-lateral velocity standard deviation (ML_Vel_SD), antero-posterior root-mean-square displacement (AP_RMS), medio-lateral root-mean-square displacement (ML_RMS), 95% confidence ellipse area (Area_95), convex hull area (CHA), and length per unit area of sway trajectory (LFS). The definitions of all indicators are presented in Table 2.

**TABLE 2:** Operational definitions, mathematical formulas, and units for evaluated center of pressure (COP) linear and geometric parameters.

Abbreviation	Parameter full name	Unit	Operational definition and formula
TPL	Total path length	mm	Cumulative 2D displacement of the COP trajectory over the sampling period. $\text{TPL}=\sum_{i=1}^{n}\sqrt{{\left(x_{i}-x_{i-1}\right)}^{2}+{\left(y_{i}-y_{i-1}\right)}^{2}}$ where n is the total number of sampling points, and $x_{i}$ , $y_{i}$ are the COP coordinates at the *i*th sample
AP_PL	Antero-posterior path length	mm	Cumulative COP trajectory length along the antero-posterior (AP, sagittal) axis. $\text{AP}\_\text{PL}=\sum_{i=1}^{n}\left|y_{i}-y_{i=1}\right|$ , where *y_i* is the AP coordinate at the *i*th sample
ML_PL	Medio-lateral path length	mm	Cumulative COP trajectory length along the medio-lateral (ML, coronal) axis. $\text{ML}\_\text{PL}=\sum_{i=1}^{n}\left|x_{i}‐x_{i‐1}\right|$ , where $x_{i}$ is the ML coordinate at the *i*th sample
AP_Amp	Antero-posterior displacement amplitude	mm	Peak-to-peak displacement range along the AP axis, representing maximum AP sway extent, $\text{AP}\_\text{Amp}=\max\left(y_{i}\right)‐\min\left(y_{i}\right)$
ML_Amp	Medio-lateral displacement amplitude	mm	Peak-to-peak displacement range along the ML axis, representing maximum ML sway extent, $\text{ML}\_\text{Amp}=\max\left(x_{i}\right)‐\min\left(x_{i}\right)$
AP_SD	Antero-posterior displacement standard deviation	mm	Standard deviation of COP displacement along the AP axis, quantifying spatial dispersion around the mean AP position. $\text{AP}\_\text{SD}=\sqrt{\frac{1}{n}\sum_{i=1}^{n}{\left(y_{i}-\bar{y}\right)}^{2}}$ where $\bar{y}$ is the mean value of all AP coordinates
ML_SD	Medio-lateral displacement standard deviation	mm	Standard deviation of COP displacement along the ML axis, quantifying spatial dispersion around the mean ML position. $\text{ML}\_\text{SD}=\sqrt{\frac{1}{n}\sum_{i=1}^{n}{\left(x_{i}-\bar{x}\right)}^{2}}$ where $\bar{x}$ is the mean value of all ML coordinates
AP_SI	Antero-posterior sway index	%	Normalized index representing the relative contribution of AP direction to total horizontal swa y dispersion. $\text{AP}\_\text{SI}=\frac{\text{AP}\_\text{RMS}}{\sqrt{{\text{AP}\_\text{RMS}}^{2}+{\text{ML}\_\text{RMS}}^{2}}}\times 100\%$
ML_SI	Medio-lateral sway index	%	Normalized index representing the relative contribution of ML direction to total horizontal sway dispersion. $\text{ML}\_\text{SI}=\frac{\text{ML}\_\text{RMS}}{\sqrt{{\text{AP}\_\text{RMS}}^{2}+{\text{ML}\_\text{RMS}}^{2}}}\times 100\%$
MV	Total mean velocity	mm/s	Average speed of COP excursion over the entire sampling duration. $\text{MV}=\frac{\text{TPL}}{T}$ , where *T* is the total test duration in seconds
AP_Vel_SD	Antero-posterior velocity standard deviation	mm/s	Standard deviation of instantaneous COP velocity along the AP axis, reflecting variability of AP movement speed. $\text{AP}\_\text{Vel}\_\text{SD}=\sqrt{\frac{1}{n}\sum_{i=1}^{n}{\left(v_{y,i}-{\bar{v}}_{y}\right)}^{2}}$ , where $v_{y,i}$ = ( $y_{i}$ − $y_{i‐1}$ )/*Δt* is the instantaneous AP velocity, and *Δt* is the sampling interval
ML_Vel_SD	Medio-lateral velocity standard deviation	mm/s	Standard deviation of instantaneous COP velocity along the ML axis, reflecting variability of ML movement speed. $\text{ML}\_\text{Vel}\_\text{SD}=\sqrt{\frac{1}{n-1}\sum_{i=1}^{n-1}{\left(v_{x,i}-{\bar{v}}_{x}\right)}^{2}}$ , where $v_{x,i}$ = ( $x_{i}$ − $x_{i‐1}$ )/*Δt* is the instantaneous ML velocity
AP_RMS	Antero-posterior root-mean-square displacement	mm	Root-mean-square value of AP displacement, quantifying average magnitude of AP sway relative to the mean position (unaffected by sign cancellation). $\text{AP}\_\text{RMS}=\sqrt{\frac{1}{n}\sum_{i=1}^{n}{\left(y_{i}-\bar{y}\right)}^{2}}$
ML_RMS	Medio-lateral root-mean-square displacement	mm	Root-mean-square value of ML displacement, quantifying average magnitude of ML sway relative to the mean position. $\text{ML}\_\text{RMS}=\sqrt{\frac{1}{n}\sum_{i=1}^{n}{\left(x_{i}-\bar{x}\right)}^{2}}$
Area_95	95% confidence ellipse area	mm²	Area of the bivariate normal 95% confidence ellipse enclosing approximately 95% of all COP points, a standard global measure of postural sway area. $\text{Area}\_95=2\pi \cdot F_{0.95}\left(2,n‐2\right)\cdot \sqrt{\sigma_{x}^{2}\sigma_{y}^{2}‐\sigma_{xy}^{2}}$ , where $\sigma_{x}^{2},\sigma_{y}^{2}$ are variances in ML and AP directions, $\sigma_{xy}$ is the covariance, and *F* is the critical value of the F-distribution
CHA	Convex hull area	mm²	Area of the smallest convex polygon that encloses all COP trajectory points, representing the actual maximum planar region covered by COP excursion. Operational definition: Computed by first constructing the convex hull of all ( $x_{i}$ $y_{i}$ ) COP coordinate points (via Graham scan or Jarvis March algorithm), then calculating the polygon area using the shoelace formula.Smaller CHA values should not be interpreted as evidence of improved postural stability; a reduced sway area may instead reflect a more constrained or stiffened control strategy, and CHA should be treated as a descriptor of sway geometry rather than a direct surrogate for stability
LFS	Length per unit area of sway trajectory	1/mm	Ratio of total path length to sway area (95% confidence ellipse area), reflects the geometric relationship between trajectory length and sway area. $\text{LFS}=\frac{\text{TPL}}{\text{Area}\_95}$ Higher values indicating a more tortuous trajectory and lower values a smoother one. LFS is a geometric descriptor and should not be interpreted as an index of postural control quality
MSE	Multiscale entropy (summary index)	—	A complexity-based summary index quantifying the regularity and adaptability of postural sway dynamics across multiple temporal scales. ${MSE}_{sum}=\sum_{\tau =1}^{8}SampEn\left(\tau \right)$ , where *SampEn(τ)* is the sample entropy of the coarse-grained COP time series at scale *τ*, computed with *m* = 2, *r* = 0.15 × *SD*
AP_MSE_Sum	AP-multiscale entropy (summary index)	—	A complexity-based summary index quantifying the regularity and adaptability of COP time-series dynamics in the AP direction across multiple temporal scales. $\text{AP}\_\text{MSE}\_\text{Sum}=\sum_{\tau =1}^{8}{SampEn}_{AP}\left(\tau \right)$
ML_MSE_Sum	ML-multiscale entropy (summary index)	—	A complexity-based summary index quantifying the regularity and adaptability of COP time-series dynamics in the ML direction across multiple temporal scales. $\text{ML}\_\text{MSE}\_\text{Sum}=\sum_{\tau =1}^{8}{SampEn}_{ML}\left(\tau \right)$

x i and y i represent instantaneous center of pressure (COP) coordinates along the mediolateral (ML) and anteroposterior (AP) axes at sample point i, respectively (i = 1, 2, …, n); n is the total number of sampling data points per trial; $\bar{x}$ and $\bar{y}$ denote the mean COP position along the ML and AP axes; Δ*t* indicates the sampling period (Δ*t* = 1/fs, where fs is the sampling frequency in Hz); T represents trial duration (*T* = *n*·Δ*t*); $s_{x}^{2}$ and $s_{y}^{2}$ denote sample variances, and $s_{xy}$ represents sample covariance between x and y coordinates; *F*_0.95_ (2, *n*-2) is the critical value of the *F*-distribution at a 95% confidence level with 2 and *n*-2 degrees of freedom; SampEn ( τ ) represents Sample Entropy at scale factor τ , calculated using template length *m* = 2 and tolerance *r* = 0.15 × SD of the original time series. Metric units marked with “—“ indicate dimensionless values.

For nonlinear analysis, SampEn and its multiscale extension, MSE, were employed to evaluate the complexity of COP signals. Proposed by Richman and Moorman (2000), SampEn overcomes the bias of approximate entropy by excluding self-matches, providing a consistent measure of time series regularity. Low SampEn values indicate regular and predictable signals, and high values reflect greater irregularity and complexity. In MSE analysis, the embedding dimension m was set to 2. Sample entropy was calculated using the Chebyshev (maximum-norm) distance for template matching, consistent with the original algorithm of Richman and Moorman (2000). The tolerance r (=0.15 × SD) was computed once from the standard deviation of the scale-1 (non-coarse-grained) filtered time series for each direction (AP, ML) and held constant across all eight scales, rather than being recalculated at each scale. Coarse-graining followed the standard non-overlapping window-averaging procedure of Costa, et al. with scale factors τ ranging from 1 to 8 (Costa et al., 2002).

The scale range (1–8) and tolerance parameters (m = 2, r = 0.15) were selected in accordance with common practice in COP-MSE literature (Gow et al., 2015). The 100-Hz sampling rate relative to the 10-Hz filter cutoff satisfies the minimum recommended ratio of ≥5 samples per cycle at the highest analyzed frequency (Gow et al., 2015), and the resulting 375 data points retained at scale 8 substantially exceeds the minimum reliability threshold (14–23×m points) recommended for stable SampEn estimation.When a scale produced an undefined SampEn value due to zero template matches, that scale was excluded from the AP_MSE_Sum/ML_MSE_Sum calculation rather than treated as zero. In practice, no undefined SampEn values occurred at any scale for any participant or condition in this dataset, so AP_MSE_Sum and ML_MSE_Sum reflect the sum of all eight scales for every participant. Each 30-s trial was processed independently, and the resulting scale-wise entropy values were averaged across the three trials per condition. The sum of sample entropy across all scales in the AP direction was defined as AP_MSE_Sum, and similarly for the ML direction as ML_MSE_Sum, both serving as characterizations of the complexity index (CI). All entropy calculations were implemented in a custom MATLAB (R2023a) pipeline.

### 2.6 Trial-to-trial reliability of linear and nonlinear measures

To evaluate trial-to-trial reliability, intraclass correlation coefficients (ICC) were computed between the repeated trials recorded for each condition, for all 17 linear COP metrics (Table 2) and all multiscale entropy (MSE) indices, the two summary measures (AP_MSE_Sum, ML_MSE_Sum) and the scale-wise entropy values at each of the eight scales in both directions (AP_MSE_Scale1–8, ML_MSE_Scale1–8), separately for each of the six task conditions. Reliability analyses were restricted to the 54 participants (28 GJH, 26 controls) comprising the main study sample. A two-way random-effects model with absolute agreement [ICC (2,1)] was used to estimate single-measure reliability, as absolute agreement was considered important for evaluating the reproducibility of repeated COP and MSE measurements. Reliability was interpreted according to the benchmarks of Koo and Li (2016): <0.50 poor, 0.50–0.75 moderate, 0.75–0.90 good, >0.90 excellent. ICC analyses were performed using participants with valid trial-level data after quality control procedures. Trials affected by excessive noise or extreme signal values were excluded, resulting in fewer than 54 participants for some condition–metric combinations (N_pairs range: 41–54).

### 2.7 Statistical analysis

Statistical analysis was performed using SPSS 27.0 (IBM Corp., Armonk, NY, USA). Data normality and homogeneity of variance were verified using Shapiro–Wilk test and Levene’s test, respectively. A 2 (Group: GJH vs. control) × 6 (Task: DEO, DEC, REO, REC, LEO, and LEC) mixed-design ANOVA was conducted to test our primary hypothesis regarding balance impairments under challenged sensory conditions. Group served as the between-subject factor, and task served as the within-subject factor.

The six stance–vision combinations were treated as a single six-level Task factor, reflecting the study’s *a priori* focus on the six complete task configurations rather than on the isolated main effects of stance or vision. This model does not separately estimate the main effects of vision, stance, or their interaction; consequently, references to ‘task difficulty’ throughout this manuscript describe a qualitative ordering of increasing postural and sensory constraint rather than a formally tested ordinal or monotonic gradient.

Mauchly’s test was used to assess sphericity where the sphericity assumption was violated, and the Greenhouse–Geisser correction was applied to adjust degrees of freedom and p-values. For between-group comparisons under specific conditions, Welch’s t-test was employed if homogeneity of variance was violated. A two-tiered correction strategy was applied. For *a priori* group comparisons under DEC, REC, and LEC, Multiple comparisons were controlled using the Benjamini-Hochberg FDR procedure across the 17 biomechanical metrics per condition (q < 0.05) (Table 3). The targeted pairwise comparisons under DEC, REC, and LEC (Table 3) were treated as confirmatory. The correlation analysis between COP and MSE metrics (Section 3.4) was treated as exploratory. The trial-to-trial reliability analysis (Section 2.6) is distinct from these group-comparison analyses; it does not test between-group hypotheses but instead evaluates the psychometric consistency of the outcome measures themselves, supporting the interpretation of the confirmatory and exploratory analyses described above. Bonferroni correction was applied to multiple comparisons, including the *post hoc* pairwise comparisons of the task main effect on COP measures (Supplementary Appendix 1), and the simple main effect analyses of the group factor on MSE measures (Supplementary Appendix 2). Spearman correlations were computed between all 17 linear COP metrics and the two MSE summary indices (AP_MSE_Sum, ML_MSE_Sum), separately for each group and each of the six standing conditions. Within each group-condition combination (34 comparisons), P values were corrected using the Benjamini-Hochberg false discovery rate (FDR) method. For 204 COP-MSE pairs, between-group differences in correlation strength were tested using Fisher’s r-to-z transformation, with the standard error adjusted for rank correlations. The resulting P values were further corrected for multiple comparisons using the FDR method. All tests were two-tailed with the significance level set at α = 0.05. Mixed-design ANOVA (covering COP and MSE parameters) and independent-samples t-tests were performed on 54 participants, comprising 28 individuals with GJH and 26 healthy controls.

**TABLE 3:** targeted pairwise comparison results between GJH and control groups under pre-specified conditions.

Indicator	Condition	GJH	Healthy	*P*	*FDR q*	*Cohen’s_d/r*	*95%CI/Z*
ML_SI (%)	LEC	49.55 ± 5.45	53.48 ± 4.15	<0.01	0.039*	−0.81	(-1.36,-0.25)
ML_Amp (mm)	LEC	68.68 ± 49.77	54.82 ± 35.20	<0.01	0.045*	0.36(r)	2.67(Z)
AP_SI (%)	LEC	50.45 ± 5.45	46.52 ± 4.15	<0.01	0.039*	0.81	(0.25, 1.36)

Abbreviations: Medio-lateral Sway Index (ML_SI), Medio-lateral Displacement Amplitude (ML_Amp), Antero-posterior Sway Index (AP_SI); *, FDRq<0.05; Cohen’s d and 95% CI, are reported for parametric comparisons; rank-biserial correlation (r) and Mann–Whitney Z are reported for nonparametric comparisons (ML_Amp). This table shows only statistically significant indicators. No statistically significant group differences were observed under the DEC, and REC, condition (FDR q > 0.05).

## 3 Results

In this work, linear COP analysis comprised two independent analytical components. Section 3.2 presents targeted pairwise comparisons under prespecified, high-difficulty conditions (DEC, REC, and LEC) designed to directly test the *a priori* clinical hypotheses. Section 3.1, Section 3.2, Section 3.3 present the mixed-design ANOVA results across all six conditions, which independently examined the overall effects of group, task condition, and their interaction. These two analyses address separate research questions (Table 4).

**TABLE 4:** Main effects of task and group × task interaction condition on linear center of pressure (COP) metrics.

Indicators	*F*_*TASK*_	*DF*_*TASK*_	*P*_*TASK*_	*Task_η* ^*2*^_*p*_	*F*_*Int*_	*DF*_*Int*_	*P*_*INT*_	*Inter_η* ^*2*^_*p*_
TPL (mm)	210.38	(1.54,80.05)	<0.001***	0.802	0.58	(1.54,80.05)	0.52	0.011
AP_PL (mm)	125.45	(1.54,79.87)	<0.001***	0.707	1.34	(1.54,79.87)	0.26	0.025
ML_PL (mm)	309.23	(1.69,88.78)	<0.001***	0.856	0.37	(1.69,88.78)	0.66	0.007
MV (mm/s)	135.46	(2.16,112.32)	<0.001***	0.723	0.95	(2.16,112.32)	0.40	0.018
AP_Vel_SD (mm/s)	69.13	(1.60,83.14)	<0.001***	0.571	2.81	(1.60,83.14)	0.08	0.051
ML_Vel_SD (mm/s)	78.68	(1.79,93.20)	<0.001***	0.602	0.68	(1.79,93.20)	0.49	0.013
AP_Amp (mm)	59.90	(2.01,104.44)	<0.001***	0.54	2.66	(2.01,104.44)	0.07	0.049
ML_Amp (mm)	44.18	(1.56,80.61)	<0.001***	0.459	1.76	(1.56,80.61))	0.19	0.033
AP_SD (mm)	57.60	(2.28,118.40)	<0.001***	0.526	2.77	(2.28,118.40)	0.06	0.050
ML_SD (mm)	68.27	(1.68,85.15)	<0.001***	0.564	2.16	(1.68,85.15)	0.13	0.040
AP_SI (%)	37.66	(3.45,179.37)	<0.001***	0.420	1.04	(3.45,179.37)	0.39	0.020
ML_SI (%)	54.34	(3.83,199.05)	<0.001***	0.511	1.61	(3.83,199.05)	0.18	0.030
AP_RMS (mm)	34.43	(2.22,115.25)	<0.001***	0.398	2.45	(2.22,115.25)	0.09	0.045
ML_RMS (mm)	67.27	(1.64,85.15)	<0.001***	0.564	2.16	(1.64,85.15)	0.13	0.040
Area_95 (mm^2^)	15.35	(1.14,59.18)	<0.001***	0.228	1.85	(1.14,59.42)	0.18	0.034
CHA (mm^2^)	13.01	(1.19,61.96)	<0.001***	0.200	1.58	(1.19,61.96)	0.22	0.029
LFS(1/mm)	1.35	(1.00,52.10)	0.25	0.025	1.11	(1.00,52.10)	0.30	0.021

Abbreviations: Total Path Length (TPL), Antero-posterior Path Length (AP_PL),Medio-lateral Path Length (ML_PL),Antero-posterior Displacement Amplitude (AP_Amp),Medio-lateral Displacement Amplitude (ML_Amp),Antero-posterior Sway Index (AP_SI),Medio-lateral Sway Index (ML_SI),Antero-posterior Displacement Standard Deviation (AP_SD),Medio-lateral Displacement Standard Deviation (ML_SD),Total Mean Velocity (MV),Antero-posterior Velocity Standard Deviation (AP_Vel_SD),Medio-lateral Velocity Standard Deviation (ML_Vel_SD), Antero-posterior Root-Mean-Square Displacement (AP_RMS),Medio-lateral Root-Mean-Square Displacement (ML_RMS),95% Confidence Ellipse Area (Area_95),Convex Hull Area (CHA). ***,P < 0.001; **,P < 0.01; *, P < 0.05; F-statistics, adjusted degrees of freedom (DF), p-values, and partial eta squared (η^2^_p_) were derived from repeated-measures ANOVA., Degrees of freedom were corrected using the Greenhouse-Geisser method when Mauchly’s test indicated a violation of the sphericity assumption (resulting in non-integer df values).

### 3.1 Main effect of task condition on postural sway

For all COP parameters, no significant group × task condition omnibus interaction effect was identified (η^2^_p_ ranging from 0.007 to 0.051, all below the conventional medium-effect threshold of 0.06). The main effect of task condition (including visual and stance variations) was highly significant across all evaluated metrics (P < 0.01) except for LFS (P = 0.25), with large effect sizes for most parameters (η^2^_p_ = 0.20–0.86; e.g., TPL η^2^_p_ = 0.802, ML_PL η^2^_p_ = 0.856). Post-hoc pairwise comparisons confirmed that the difficulty of the balance tasks (support base and visual condition) significantly affected postural stability. First, regarding visual conditions, the AP-PL, AP_Vel_SD and AP_SI under the DEC condition were significantly higher than those under the eyes-open condition (P < 0.05), except ML_SI is lower. Second, regarding the support base, single-leg stance (whether eyes open or eyes closed) significantly increased most measured parameters compared with double-leg stance (P < 0.05), except AP_RMS (mm) (DEO-REO, DEO-LEO). For instance, the TPL (mm) increased dramatically from DEO to REC (mean_diff = −1734.79 mm, P < 0.05, d = −2.78). Finally, regarding visual compensation during high-difficulty single-leg tasks, most parameters under the single-leg eyes-closed condition were significantly greater than those under the eyes-open condition (P < 0.05). (Supplementary Appendix 2 for complete pairwise comparison results).

### 3.2 Group differences in postural sway across task conditions

For all COP parameters, no significant group × task condition omnibus interaction effect was identified. Except for AP_SI,which showed a significant main effect of group (F = 4.32, P = 0.04, η^2^_p_ = 0.077), no other significant main effects of Group were observed (P > 0.05) (Table 5).

**TABLE 5:** Main effects of group and group × task interaction on linear center of pressure (COP) metrics.

Indicators	*F*_*GROUP*_	*DF*_*GROUP*_	*P*_*GROUP*_	*Group_η* ^*2*^_*p*_	*F*_*Int*_	*DF*_*INT*_	*P*_*INT*_	*Inter_η* ^*2*^_*p*_
TPL (mm)	0.09	(1,52)	0.76	0.002	0.58	(1.54,80.05)	0.52	0.011
AP_PL (mm)	0.68	(1,52)	0.41	0.012	1.34	(1.54,79.87)	0.26	0.025
ML_PL (mm)	0.17	(1,52)	0.68	0.003	0.37	(1.69,88.78)	0.66	0.007
MV (mm/s)	0.10	(1,52)	0.76	0.002	0.95	(2.16,112.32)	0.40	0.018
AP_Vel_SD (mm/s)	2.39	(1,52)	0.13	0.044	2.81	(1.60,83.14)	0.08	0.051
ML_Vel_SD (mm/s)	0.20	(1,52)	0.65	0.004	0.68	(1.79,93.20)	0.49	0.013
AP_Amp (mm)	1.52	(1,52)	0.22	0.028	2.66	(2.01,104.44)	0.07	0.049
ML_Amp (mm)	1.90	(1,52)	0.17	0.035	1.76	(1.56,80.61))	0.19	0.033
AP_SD (mm)	0.44	(1,52)	0.51	0.008	2.77	(2.28,118.40)	0.06	0.050
ML_SD (mm)	2.15	(1,52)	0.15	0.040	2.16	(1.68,85.15)	0.13	0.040
AP_SI (%)	4.32	(1,52)	0.04*	0.077	1.04	(3.45,179.37)	0.39	0.020
ML_SI (%)	3.88	(1,52)	0.05	0.069	1.61	(3.83,199.05)	0.18	0.030
AP_RMS (mm)	0.04	(1,52)	0.84	0.001	2.45	(2.22,115.25)	0.09	0.045
ML_RMS (mm)	2.15	(1,52)	0.15	0.040	2.16	(1.64,85.15)	0.13	0.040
Area_95 (mm^2^)	2.00	(1,52)	0.16	0.037	1.85	(1.14,59.42)	0.18	0.034
CHA (mm^2^)	1.72	(1,52)	0.20	0.032	1.58	(1.19,61.96)	0.22	0.029
LFS(1/mm)	1.19	(1,52)	0.28	0.022	1.13	(1.00,52.10)	0.30	0.021

Abbreviations: Total Path Length (TPL), Antero-posterior Path Length (AP_PL),Medio-lateral Path Length (ML_PL),Antero-posterior Displacement Amplitude (AP_Amp),Medio-lateral Displacement Amplitude (ML_Amp),Antero-posterior Sway Index (AP_SI),Medio-lateral Sway Index (ML_SI),Antero-posterior Displacement Standard Deviation (AP_SD),Medio-lateral Displacement Standard Deviation (ML_SD),Total Mean Velocity (MV),Antero-posterior Velocity Standard Deviation (AP_Vel_SD),Medio-lateral Velocity Standard Deviation (ML_Vel_SD),Antero-posterior Root-Mean-Square Displacement (AP_RMS),Medio-lateral Root-Mean-Square Displacement (ML_RMS),95% Confidence Ellipse Area (Area_95),Convex Hull Area (CHA). ***,P < 0.001; **,P < 0.01; *, P < 0.05; F-statistics, adjusted degrees of freedom (DF), p-values, and partial eta squared (η^2^_p_) were derived from repeated-measures ANOVA., Degrees of freedom were corrected using the Greenhouse-Geisser method when Mauchly’s test indicated a violation of the sphericity assumption (resulting in non-integer df values).

Targeted pairwise comparisons were conducted between the GJH and healthy control groups under specific high-difficulty environments (eyes-closed condition). The statistical results for these targeted comparisons are detailed in Table 3. During the LEC stance, multiple parameters showed significant differences: the GJH group exhibited a significantly lower ML_SI (%) (49.55 ± 5.45 vs. 53.48 ± 4.15, q = 0.039, Cohen’s d = −0.81, 95% CI [−1.36, −0.25]).Conversely, the GJH group showed significantly higher ML_Amp (mm) (68.68 ± 49.77 vs. 54.82 ± 35.20, q = 0.045, r = 0.36, Z = 2.67) and AP_SI (%) (50.45 ± 5.45 vs. 46.52 ± 4.15, q = 0.039, Cohen’s d = 0.81, 95% CI [0.25, 1.36]) than the healthy controls did. No statistically significant differences were observed in any of the other measures (q > 0.05).

### 3.3 Task- and group-dependent changes in MSE-Derived complexity

Mixed-design ANOVA revealed significant group × task interaction effects across all MSE parameters in AP and ML directions (P < 0.05). The main effects of group were significant across all parameters (P < 0.01), with large effect sizes throughout (Group η^2^_p_ = 0.16–0.26 across all 18 MSE indicators). The main effects of task were significant across nearly all parameters (P < 0.01) except ML_MSE_Scale7 (P = 0.20, η^2^_p_ = 0.03) (Table 6). During DEC, the GJH group exhibited significantly higher entropy values across all scales in both directions (P < 0.05) than the healthy controls did. During DEO, this pattern held for all AP scales, but ML_MSE_Scale8 was no longer significant (P > 0.05). During dominant-limb tasks (REO and REC), significant group differences were observed across most scales. In the AP direction, REO was significant at Scale 2–7, and REC was significant across all scales. In the ML direction, REO was significant at Scale 2–5, and REC was significant at Scale 1–6. During nondominant limb tasks, the number of scales showing significant group differences decreased from LEO to LEC. Under LEO, significant differences were observed at AP Scale 4–8 and ML Scale 3–6. Under LEC, no significant AP differences remained at any scale, including AP_MSE_Sum (P > 0.05). In the ML direction, significance was preserved only at Scale 1–3; ML_MSE_Sum was no longer significant (P > 0.05) (Supplementary Appendix 2, Simple effect analysis of MSE; Figure 3, Sum scale; Figure 4, AP direction; Figure 5, ML direction; Figure 6, MSE SUM effect sizes; Figure 7, MSE 1–8 effect sizes).

**TABLE 6:** Mixed-design ANOVA of Group and Task Condition on multiscale entropy (MSE) values across time scales.

Indicators	*F*_*Task*_	*DF*_*Task*_	*P*_*Task*_	*Task* ^*η2*^_*p*_	*F*_*GROUP*_	*DF*_*Group*_	*P*_*Group*_	*Group* ^*η2*^_*p*_	*F*_*Inter*_	*DF*_*Int*_	*P*_*Int*_	*Inter* ^*η2*^_*p*_
AP_MSE_Sum	38.39	(3.77,196.03)	<0.001***	0.425	17.03	(1, 52)	<0.001***	0.247	4.63	(3.77,196.03)	<0.01**	0.08
ML_MSE_Sum	87.22	(2.50,130.05)	<0.001***	0.626	15.80	(1, 52)	<0.001***	0.233	8.35	(2.50,130.05)	<0.001***	0.14
AP_MSE_Scale1	158.01	(3.15,163.56)	<0.001***	0.752	12.36	(1, 52)	<0.01**	0.192	8.33	(3.15,163.56)	<0.001***	0.14
AP_MSE_Scale2	159.02	(2.99,155.49)	<0.001***	0.754	14.77	(1, 52)	<0.001***	0.221	6.25	(2.99,155.49)	<0.01**	0.11
AP_MSE_Scale3	90.26	(3.51,182.46)	<0.001***	0.634	16.84	(1, 52)	<0.001***	0.245	5.35	(3.51,182.46)	<0.01**	0.09
AP_MSE_Scale4	37.82	(3.90,202.60)	<0.001***	0.421	17.50	(1, 52)	<0.001***	0.252	3.75	(3.90,202.60)	<0.01**	0.07
AP_MSE_Scale5	14.95	(3.91,203.56)	<0.001***	0.223	18.44	(1, 52)	<0.001***	0.262	2.93	(3.91,203.56)	0.01*	0.05
AP_MSE_Scale6	6.22	(3.83,199.97)	<0.001***	0.107	17.20	(1, 52)	<0.001***	0.249	3.22	(3.85,199.97)	0.02*	0.06
AP_MSE_Scale7	4.35	(3.65,189.95)	<0.01**	0.077	17.74	(1, 52)	<0.001***	0.254	2.50	(3.65,189.95)	0.049*	0.05
AP_MSE_Scale8	5.22	(3.58,186.30)	<0.01**	0.091	15.10	(1, 52)	<0.001***	0.225	3.21	(3.58,186.30)	0.02*	0.06
ML_MSE_Scale1	191.52	(2.56,132.95)	<0.001***	0.786	14.17	(1, 52)	<0.001***	0.214	7.44	(2.56,132.95)	<0.001***	0.13
ML_MSE_Scale2	257.33	(2.11,109.61)	<0.001***	0.832	16.52	(1, 52)	<0.001***	0.241	9.86	(2.11,109.61)	<0.001***	0.16
ML_MSE_Scale3	198.87	(2.34,121.77)	<0.001***	0.793	16.73	(1, 52)	<0.001***	0.243	8.04	(2.34,121.77)	<0.001***	0.13
ML_MSE_Scale4	122.01	(2.50,130.26)	<0.001***	0.701	16.25	(1, 52)	<0.001***	0.238	7.94	(2.50,130.26)	<0.001***	0.13
ML_MSE_Scale5	49.46	(2.97,154.45)	<0.001***	0.487	16.31	(1, 52)	<0.001***	0.239	6.91	(2.97,154.45)	<0.001***	0.12
ML_MSE_Scale6	12.32	(3.15,163.67)	<0.001***	0.192	15.16	(1, 52)	<0.001***	0.226	5.68	(3.15,163.67)	<0.01**	0.10
ML_MSE_Scale7	1.53	(3.53,183.56)	0.20	0.029	13.01	(1, 52)	<0.01**	0.200	6.16	(3.53,183.56)	<0.001***	0.11
ML_MSE_Scale8	8.48	(3.68,191.20)	<0.001***	0.140	10.04	(1, 52)	<0.01**	0.162	3.25	(3.68,191.20)	0.02*	0.06

Abbreviations: Double-leg stance with eyes open (DEO); Double-leg stance with eyes closed (DEC); Right-leg stance with eyes open (REO); Right-leg stance with eyes closed (REC); Left-leg stance with eyes open (LEO); Left-leg stance with eyes closed (LEC); ***, *P* < 0.001, **P < 0.01, *P < 0.05, DF_Task_, and DF_Int_, represent the degrees of freedom (effect, error). Due to the violation of sphericity, the Greenhouse-Geisser correction was applied to adjust degrees of freedom and p-values for the Task main effect and Group × Task interaction. Multiple comparison adjustments across metrics were applied separately to Group main effects, Task main effects, and Interaction effects using the Bonferroni correction.

**FIGURE 3:**
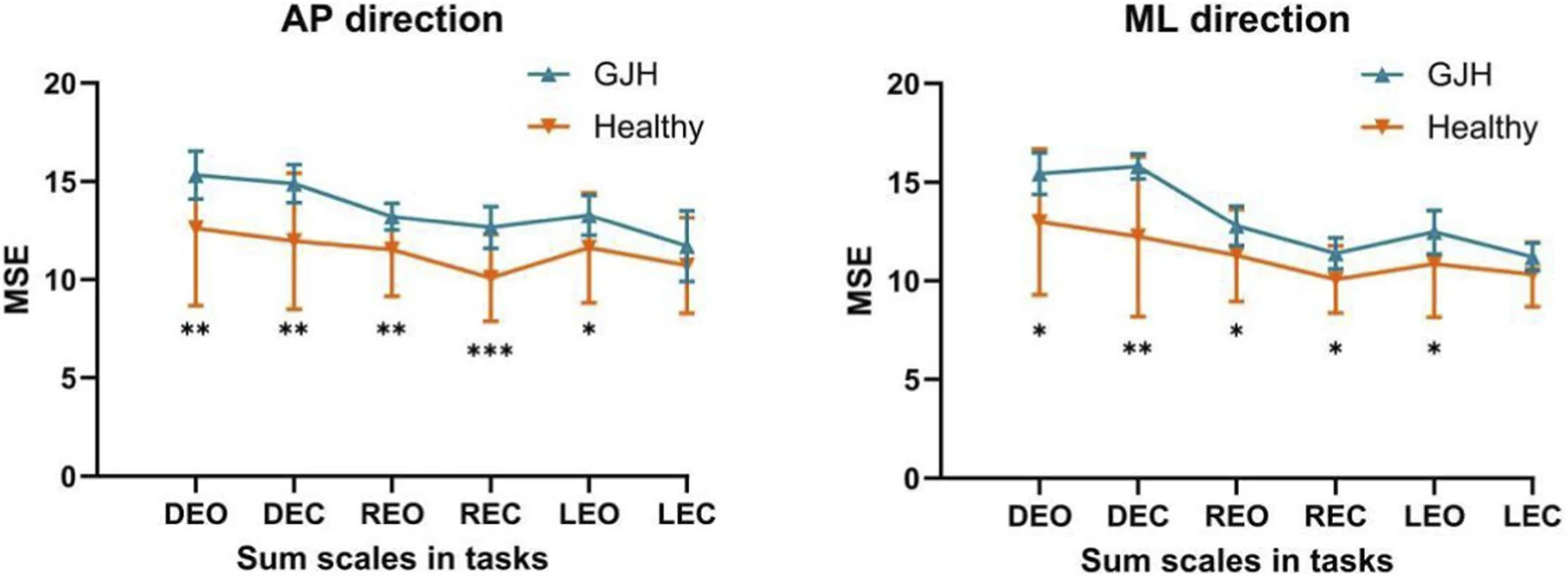
Comparison of sum of MSE scales across task conditions between GJH and healthy controls. Data are presented as mean ± SD. ***, P < 0.001; **, P < 0.01; *, P < 0.05, indicate significant between-group differences within the specified task condition; Abbreviations: GJH, generalized joint hypermobility; MSE, multiscale entropy; AP, anteroposterior; ML, mediolateral; DEO, double-leg eyes-open; DEC, double-leg eyes-closed; REO, right-leg eyes-open; REC, right-leg eyes-closed; LEO, left-leg eyes-open; LEC, left-leg eyes-closed. Statistical significance is determined based on Pcorr (Bonferroni-corrected P value).

**FIGURE 4:**
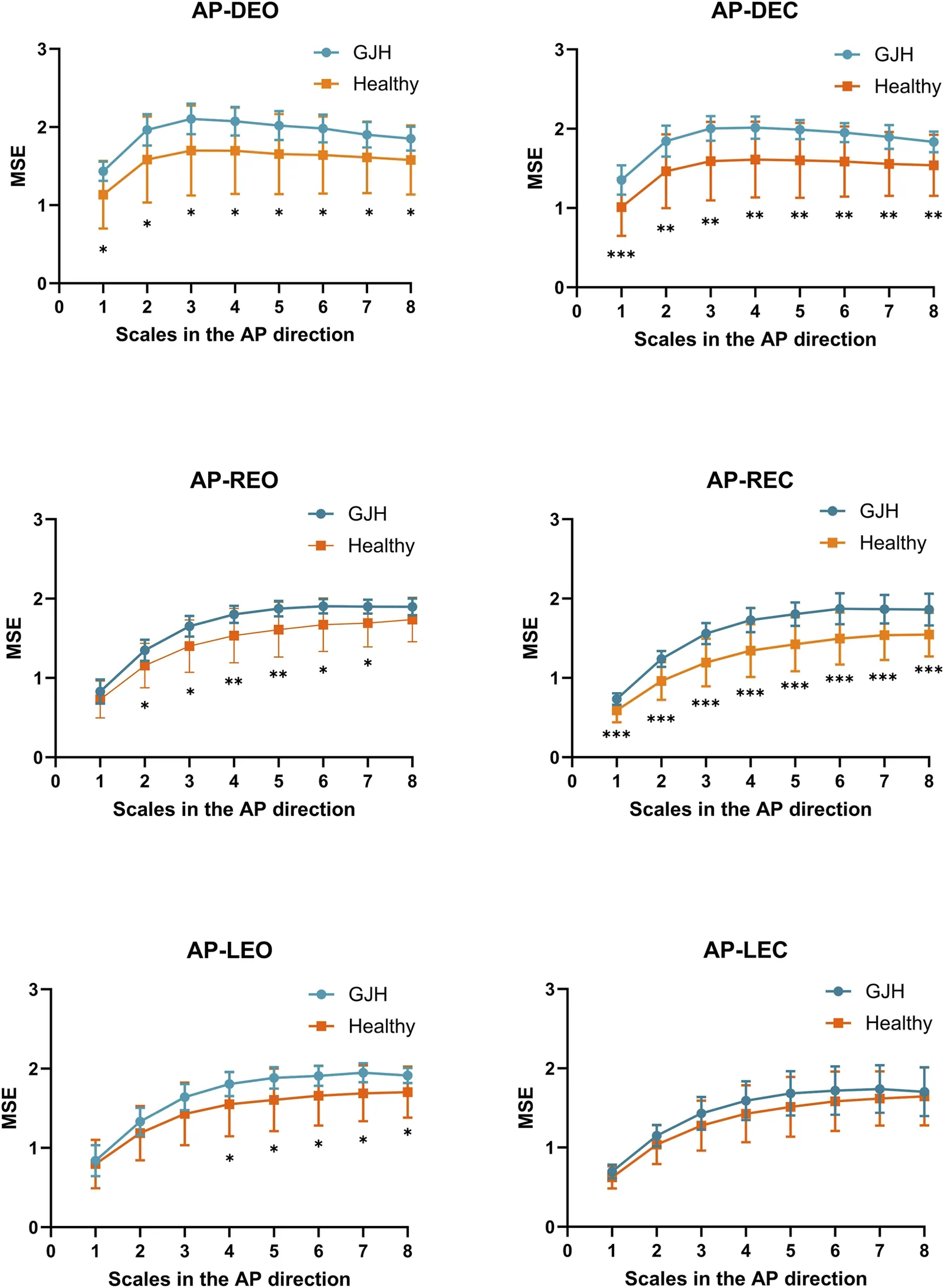
Multiscale entropy (MSE) values across scale factors in the anteroposterior (AP) direction under six task conditions between GJH and healthy controls. Data are presented as mean ± SD. ***, *P* < 0.001; **, *P* < 0.01; *, *P* < 0.05, indicate significant differences between the GJH group and healthy controls at the corresponding scale factor. Abbreviations: GJH, generalized joint hypermobility; MSE, multiscale entropy; AP, anteroposterior; DEO, double-leg eyes-open; DEC, double-leg eyes-closed; REO, right-leg eyes-open; REC, right-leg eyes-closed; LEO, left-leg eyes-open; LEC, left-leg eyes-closed. Statistical significance is determined based on Pcorr (Bonferroni-corrected P value).

**FIGURE 5:**
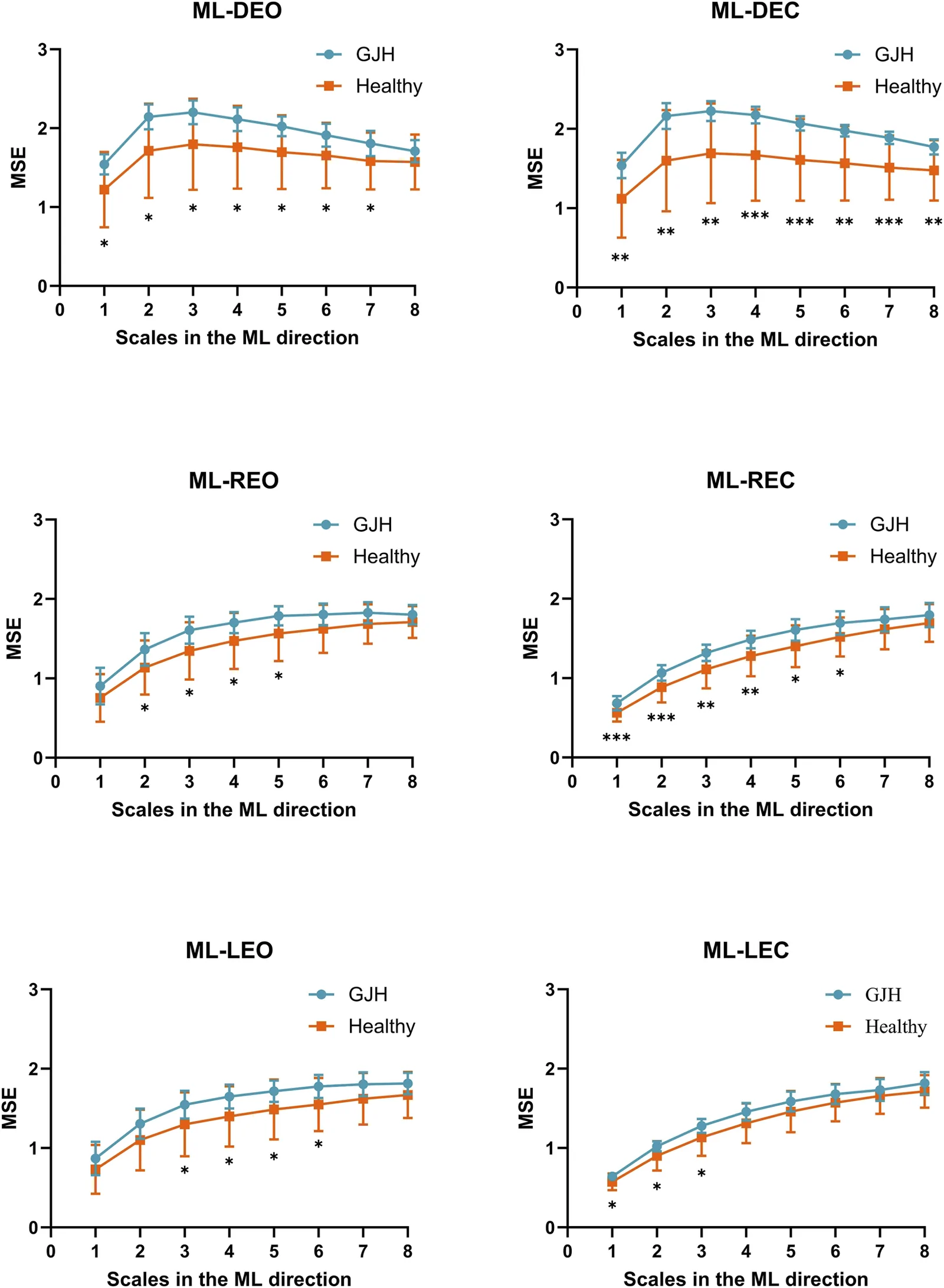
Multiscale entropy (MSE) values across scale factors in the mediolateral (ML) direction under six task conditions between GJH and healthy controls. Data are presented as mean ± SD. ***, P < 0.001; **, P < 0.01; *, P < 0.05, indicate significant differences between the GJH group and healthy controls at the corresponding scale factor. Abbreviations: GJH, generalized joint hypermobility; MSE, multiscale entropy; ML, mediolateral; DEO, double-leg eyes-open; DEC, double-leg eyes-closed; REO, right-leg eyes-open; REC, right-leg eyes-closed; LEO, left-leg eyes-open; LEC, left-leg eyes-closed. Statistical significance is determined based on Pcorr (Bonferroni-corrected P value).

**FIGURE 6:**
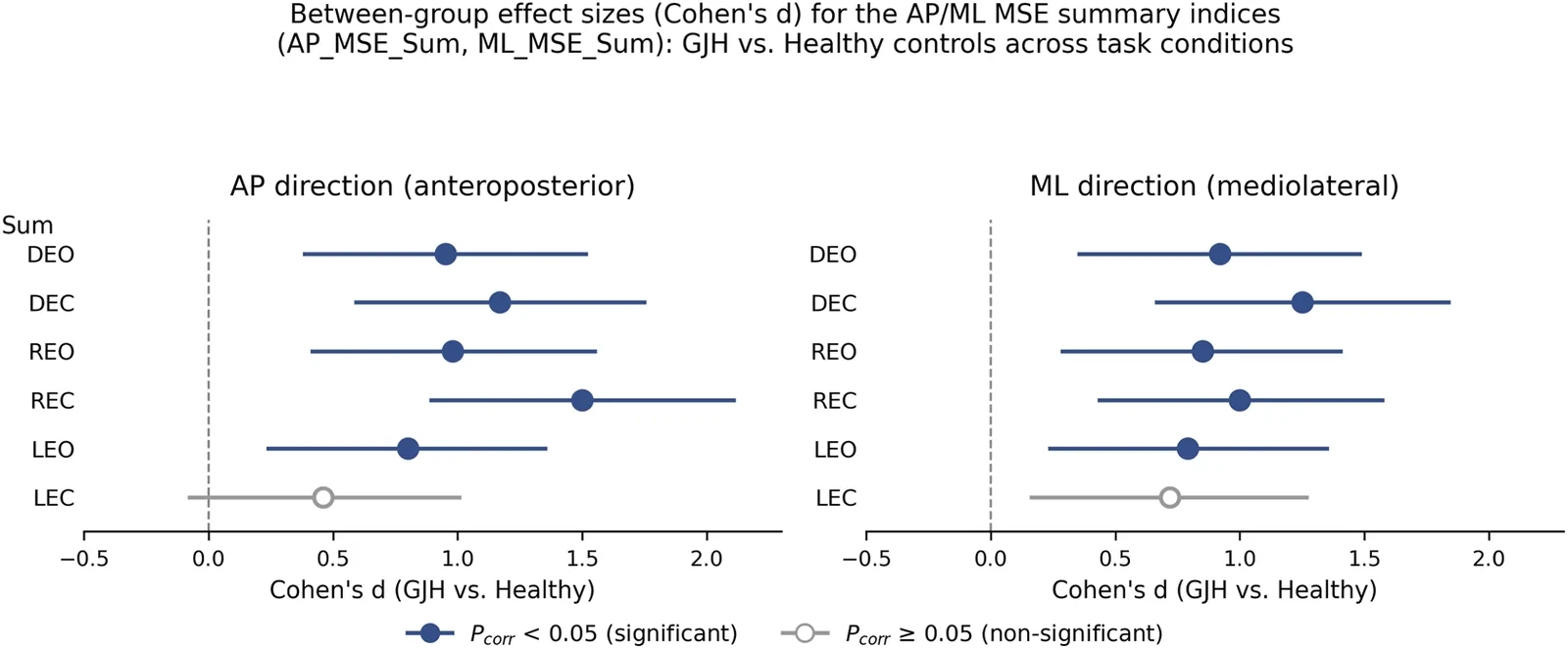
Between-group effect sizes (Cohen’s d) for the AP/ML SUM MSE: GJH vs. Healthy controls across different task conditions. Abbreviations: Data are presented as effect size (Cohen’s d) with 95% confidence interval.DEO, double-leg eyes-open; DEC, double-leg eyes-closed; REO, right-leg eyes-open; REC, right-leg eyes-closed; LEO, left-leg eyes-open; LEC, left-leg eyes-closed.

**FIGURE 7:**
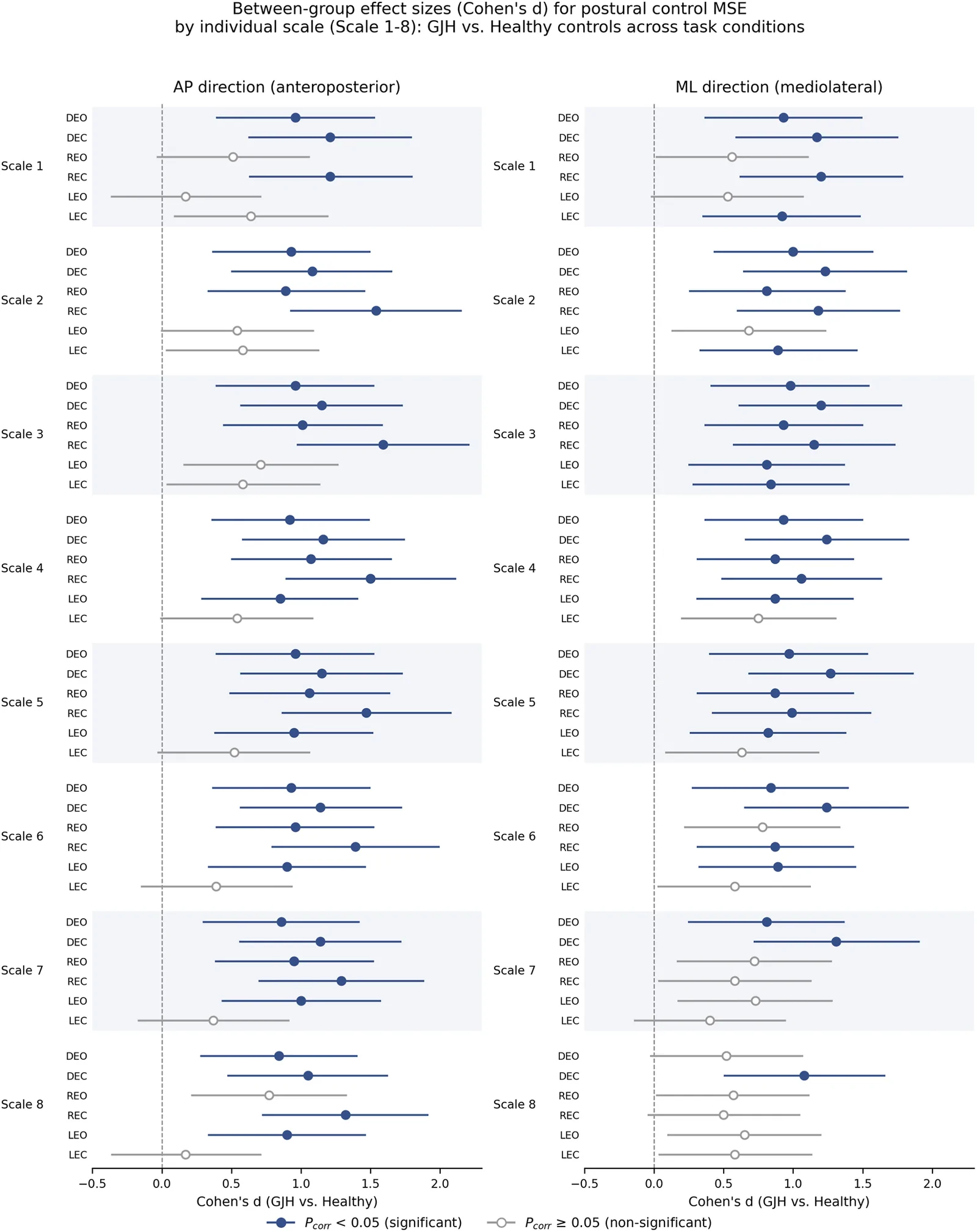
Between-group effect sizes (Cohen’s d) for the AP/ML 1–8 MSE: GJH vs. Healthy controls across different task conditions. Abbreviations: Data are presented as effect size (Cohen’s d) with 95% confidence interval. DEO, double-leg eyes-open; DEC, double-leg eyes-closed; REO, right-leg eyes-open; REC, right-leg eyes-closed; LEO, left-leg eyes-open; LEC, left-leg eyes-closed.

### 3.4 Correlation between COP and MSE metrics: associations between linear and nonlinear measures

To determine whether COP and MSE provide related or distinct information, Spearman correlations were computed between the 17 linear COP metrics and the two MSE summary indices, separately for each group and each of the six standing conditions. Within each group-condition combination (34 comparisons), P values were corrected using the Benjamini-Hochberg FDR method. In the GJH group, 29 of the 204 correlations reached FDR significance (q < 0.05); in the healthy control group, none reached significance (Supplementary Appendix 3). Between-group differences in correlation strength were tested for all 204 COP-MSE pairs (not restricted to those significant within either group) using Fisher’s z transformation with the standard error adjusted for rank correlations, and the resulting P values were corrected for multiple comparisons using the FDR method across all 204 comparisons. No correlation showed a statistically significant between-group difference after FDR correction across all 204 pairs. The three pairs closest to significance were Area_95 (GJH ρ = −0.54, healthy ρ = 0.41, Z = −3.63, q = 0.058), AP_Vel_SD (GJH ρ = −0.59, healthy ρ = 0.27, Z = −3.29, q = 0.068), and LFS (GJH ρ = 0.57, healthy ρ = −0.32, Z = 3.37, q = 0.068), all under the DEO condition and involving AP_MSE_Sum (Supplementary Appendix 4).

### 3.5 Trial-to-trial reliability of COP and MSE metrics

Trial-to-trial reliability, assessed via ICC (2,1) across all metrics (N = 41–54; Table 7; full results in Supplementary Appendixs 5, 6), ranged from poor to excellent (mean = 0.59 for COP, 25% good-to-excellent; mean = 0.65 for MSE, 41% good-to-excellent). For the three linear indicators showing significant FDR-corrected group differences under LEC, reliability was good for ML_Amp (ICC = 0.79) and moderate for AP_SI and ML_SI (ICC = 0.69 each); the AP/ML MSE summary indices likewise showed moderate reliability under LEC (ICC = 0.73 and 0.72, respectively).

**TABLE 7:** Trial-to-trial reliability (ICC (2,1)) of key linear COP and MSE indicators representative reliability results for the primary findings (N = 54 participants; N_pairs reflects those with valid paired-trial data).

Metric	ML_Amp (mm)	AP_SI (%)	ML_SI (%)	AP_MSE_Sum	ML_MSE_Sum
Condition	LEC	LEC	LEC	LEC	LEC
N_pairs	52	52	52	53	53
ICC (2,1)	0.791	0.692	0.692	0.725	0.723

Abbreviations: left-leg stance with eyes closed (LEC), medio-lateral displacement amplitude (ML_Amp), antero-posterior sway index (AP_SI), medio-lateral sway index (ML_SI), AP_MSE_Sum, anteroposterior multiscale entropy sum; ML_MSE_Sum, mediolateral multiscale entropy sum; N_pairs, number of participants with valid paired-trial data; ICC (2,1), intraclass correlation coefficient (two-way random-effects, absolute agreement).

## 4 Discussion

This study evaluated postural control in individuals with GJH by integrating linear COP metrics with nonlinear MSE analysis. Overall, our hypotheses were partially supported. Linear parameters suggested condition-specific postural alterations: Individuals with GJH exhibited significantly greater sway (ML_Amp, AP_SI) and lower ML_SI, under the most challenging condition (LEC). Although omnibus ANOVA revealed no general group × task interaction for linear metrics, MSE analysis showed significant interactions across all complexity parameters. Compared with the healthy controls, the GJH group displayed higher postural complexity in easy tasks. However, this elevation in complexity showed a descriptive pattern of attenuation with increasing task difficulty, with the between-group difference in AP entropy no longer detectable at any scale under LEC. Linear metrics appear to capture condition-specific sway boundaries, while nonlinear MSE may provide complementary information about changes in postural control dynamics.

### 4.1 Linear posturography: alterations in postural sway amplitude and directional stability

In terms of traditional linear COP metrics, this study demonstrated a highly significant main effect of task condition across nearly all evaluated parameters (P < 0.01), reflecting substantial postural perturbations induced by narrowed bases of support and visual deprivation. However, contrary to our initial hypothesis of macro-instability, no significant group × task condition omnibus interaction effects were identified for any linear metrics (P > 0.05). However, targeted pairwise comparisons under prespecified, high-difficulty conditions revealed specific between-group differences under the LEC condition, suggesting that localized postural control variations in GJH may only become detectable under the most demanding sensorimotor constraints. Furthermore, with the exception of AP_SI, no significant main effects of Group were detected across the remaining linear parameters. These statistical outcomes suggest that, apart from a modest but significant group-level elevation in AP_SI, GJH individuals tend to maintain overall postural sway within a range broadly similar to healthy controls under these tested conditions. Postural regulation typically relies on integrating visual, proprioceptive, and vestibular inputs (Peterka, 2002). Although joint hypermobility is often associated with altered joint mechanics and reduced proprioceptive acuity, individuals with GJH might utilize functional compensation strategies to regulate macrolevel sway (Simmonds and Keer, 2007). For instance, when vision is available, they may rely heavily on visual feedback. This sensory reweighting could help offset joint laxity and support spatial orientation (Peterka, 2002).

Our targeted pairwise comparisons revealed group differences under LEC. Under LEC, the GJH group showed significantly greater ML_Amp and AP_SI, and significantly lower ML_SI, than the healthy controls did. No significant differences were found under DEC and REC. Effect sizes for these LEC differences varied in magnitude: ML_SI and AP_SI showed large effect sizes (Cohen’s d = −0.81), whereas ML_Amp showed comparatively smaller effect sizes (rank-biserial r = 0.36), indicating a meaningful but condition-specific effect. This pattern suggests that group differences emerged specifically under the most demanding condition (LEC), rather than across the other five task conditions. During simple tasks, individuals with GJH can compensate for ligament laxity using vision (Peterka, 2002). The LEC condition represents an extreme compound constraint, combining visual deprivation, a narrowed base of support, and nondominant limb stance. Rather than reflecting a generalized deficit in nondominant limb control, these observed differences likely stem from the cumulative demand of this specific task combination. Under these constraints, the GJH group exhibited greater ML_Amp and AP_SI, along with reduced ML_SI, compared with the healthy controls. One possible explanation is that the postural control system shifted toward less frequent but larger corrective adjustments under this extreme condition; habitual knee posture (Section 2.3, discussed further in Section 4.5) may also have contributed. This mechanism remains inferential, as neuromuscular response latency, joint kinematics, and muscle activation were not directly measured, and the LEC findings should be read as task-specific rather than evidence of a generalized nondominant-limb limitation. We would also point out that the effect sizes themselves tell a more nuanced story than the P-values alone. Under LEC, ML_SI and AP_SI show fairly large effects (Cohen’s d = 0.81), while ML_Amp are more modest (rank-biserial r = 0.36). We think this difference in magnitude matters for how these findings should be weighted, not all of our significant results carry the same evidentiary weight, even though they all clear the same P-value threshold. These findings should be interpreted as evidence of differences in postural-control behavior rather than as direct indicators of clinical fall risk, as no fall outcomes, functional balance assessments, or symptom-severity measures were collected in this study. The lack of an overall group difference in ML parameters is likely due to statistical averaging. Given that the omnibus test combines all conditions, the similar performance between groups in easy tasks (e.g., DEO, LEO, and REO) likely diluted the specific difference seen in the LEC condition. Nonsignificant group differences under high-difficulty tasks should not be viewed as a collapse of postural control. Instead, multiple factors can explain this outcome. Extreme task constraints often increase data variance, limit statistical power, and lower measurement sensitivity. Alternatively, both groups may have shown comparable sway magnitude under this condition, without implying a shared physiological ceiling. Therefore, prespecified, targeted comparisons under challenging conditions may help highlight localized control variations that broad omnibus tests might obscure. Nevertheless, as traditional linear metrics showed limited group-level differences overall, analyzing nonlinear dynamic parameters may offer deep insights into the microstructural control adaptations in GJH.

### 4.2 Nonlinear dynamics: multiscale entropy quantifies task-dependent information complexity

The MSE findings provide complementary information regarding postural control dynamics beyond conventional COP measures. Unlike linear COP variables that primarily characterize sway magnitude and temporal variability, MSE quantifies the complexity of COP fluctuations across multiple temporal scales. However, interpretation of individual MSE scales requires caution. Because coarse-graining at scale 1 involves no averaging, entropy estimates at the finest scales may partly reflect residual characteristics of the preprocessing filter rather than purely physiological irregularity. Previous studies have demonstrated the robustness of SampEn-based approaches for COP time-series analysis across different parameter settings, supporting the methodological rationale for using SampEn in the present study(Montesinos et al., 2018). Low-pass filtering has also been shown to systematically reduce sample entropy estimates in COP data (Rhea et al., 2015), consistent with the tempered interpretation of fine-scale entropy values noted above. Moreover, the physiological correlates of specific MSE scales in COP dynamics remain incompletely characterized (Gow et al., 2015). Interpretation therefore emphasizes the overall pattern across scales (AP_MSE_Sum/ML_MSE_Sum) rather than any single fine-scale value in isolation. Different from traditional linear metrics, MSE revealed significant group × task interaction effects across all evaluated parameters in AP and ML directions (P < 0.05). The main effects for group and task were also significant across nearly all scales (P < 0.01), with a minor exception for the task effect at ML_MSE_Scale7 (P = 0.20). The group main effects were not only statistically significant but also large in magnitude (η^2^_p_ = 0.16–0.26 across all scales and both directions), suggesting the observed complexity differences reflect a substantial and consistent between-group distinction rather than a marginal statistical artifact. Throughout this discussion, between-group MSE differences are described as varying or narrowing progressively across conditions, based on the observed pattern of significant versus nonsignificant comparisons at each scale. This characterization is descriptive rather than based on a formal trend or monotonicity test, and should not be interpreted as a statistically confirmed monotonic decline.

During bipedal and dominant-limb tasks, the GJH group exhibited higher entropy values than the healthy controls did across almost all scales. Researchers noted that the high MSE of COP signals reflects great complexity and stochasticity, indicating many nonrepetitive postural adjustments (Busa and Richard, 2016). In individuals with GJH, ligamentous laxity and reduced proprioceptive feedback may diminish the reliability of afferent signals from joint mechanoreceptors (Smith et al., 2013). To maintain stability, the nervous system might continuously integrate multimodal sensory inputs, generating active corrective adjustments across multiple time scales. However, this elevated MSE does not unequivocally indicate adaptive compensatory control. This finding is interpreted using the Optimal Movement Variability: framework proposed by Stergiou et al. (Stergiou et al., 2006). This framework was originally developed for aging and pathological populations, where complexity declines steadily. Our pattern is different: complexity was higher under low-to-moderate demand and dropped only under the most demanding condition. This suggests the pattern in young GJH individuals may differ from that seen in older or degenerative populations. Deviations from this state, whether toward excessive periodicity or excessive stochasticity, are commonly interpreted as markers of altered neuromuscular control. However, high entropy can also reflect signal irregularity or measurement noise rather than adaptive regulation. Given this ambiguity, we interpret elevated MSE in the GJH group as a candidate marker of altered postural regulation, not as direct evidence of compensation. The significant group × task interaction indicates that the magnitude of the between-group MSE difference varied across conditions, with significant differences observed under some conditions but not others. During nondominant limb tasks (LEO and LEC), between-group MSE differences were smaller than under other conditions. Under the most challenging condition (LEC), AP differences were no longer significant at any scale, including AP_MSE_Sum (P > 0.05). This task-dependent shift is consistent with a narrower range of postural adjustments under extreme constraints, though the underlying regulatory mechanism was not directly tested. When facing high balance demands, individuals with GJH and healthy controls might rely on joint-stiffening strategies to maintain balance. This stiffening response limits body movement options, which likely attenuates group-level entropy differences under extreme conditions. This task-dependent pattern helps contextualize our MSE findings relative to established theoretical frameworks. Previous studies in older adults often associated reduced postural complexity with impaired adaptability and fall risk (Manor et al., 2010). By contrast, young adults with GJH display elevated postural complexity during low-to-moderate difficulty tasks, which subsequently narrows under high constraints. This distinction suggests that elevated MSE in GJH may reflect adaptive changes in postural control complexity rather than age-related complexity degradation. Therefore, MSE variations should be interpreted in light of population-specific sensorimotor characteristics and specific task constraints.

### 4.3 Biomechanical and sensory mechanisms underlying compensatory attenuation across task conditions

During bipedal and dominant-limb tasks, the GJH group exhibited higher MSE values than the healthy controls did across most scales in both directions, consistent with the predicted pattern of great complexity under low-demand conditions. As task difficulty increased, between-group differences progressively narrowed during nondominant-limb tasks and were substantially reduced under LEC, broadly consistent with the predicted attenuation. However, the group differences were not entirely absent under LEC, particularly in the ML direction, indicating that the predicted narrowing was direction-dependent rather than uniform. Overall, these patterns reveal that this elevated postural complexity was task-dependent and showed a progressive transition across different limb and visual constraints. During bipedal tasks (DEO/DEC) and dominant unipedal tasks (REO/REC), the GJH group maintained higher MSE values than the healthy controls did. This pattern is consistent with the hypothesis that postural regulation is better maintained when supported by a wide base of support or dominant-limb control. We present this as a plausible explanation, not a confirmed mechanism, since neuromuscular activity was not measured.

When testing the nondominant limb, compensatory capacity showed a gradual decline. Under LEO, significant group differences persisted at AP Scale 4–8 and ML Scale 3–6, though the pattern became more variable compared with that under the dominant-limb conditions. Under LEC, differences were substantially reduced: no AP scale remained significant, including AP_MSE_Sum. In the ML direction, significance was preserved only at Scales 1–3, and ML_MSE_Sum was no longer significant. This finding suggests that compensatory complexity was not entirely eliminated under LEO but became almost fully constrained under LEC, when visual deprivation and nondominant unipedal support were combined. This progressive decline may reflect the cumulative demands placed on the postural control system as task difficulty increases. When visual input and dominant-limb advantages are simultaneously removed, the system’s capacity to sustain active multiscale adjustments across varying time scales may be reduced (Costa et al., 2005), potentially giving way to a constrained control pattern (Ferrell et al., 2004).

### 4.4 Integration of linear and nonlinear dynamics: current status and scientific insights

Correlation analyses provided further insight into the relationship between linear and nonlinear COP measures. Spearman correlations were computed between all 17 linear COP metrics and the two MSE summary indices for each group across all six standing conditions. After FDR correction, 29 correlations reached significance in the GJH group, whereas none reached significance in healthy controls. We directly compared correlation strength between groups using Fisher’s r-to-z transformation for every COP-MSE pair, with FDR correction applied across all between-group comparisons. No correlation showed a statistically significant between-group difference after FDR correction across all 204 pairs (Gelman and Stern, 2006); the asymmetry between 29 significant within-group correlations in GJH and none in healthy controls should not itself be interpreted as evidence of a between-group difference. Three pairs involving AP_MSE_Sum under DEO (Area_95, AP_Vel_SD, LFS) approached significance (q = 0.06–0.07) and may warrant investigation in a larger sample.

These three near-significant pairs relate to the spatial extent, velocity dispersion, and trajectory tortuosity of the COP trajectory during DEO. In the GJH group, Area_95 and AP_Vel_SD showed a negative trend with AP temporal complexity, while LFS showed a positive trend; healthy controls showed the opposite direction for all three. As these differences did not reach significance after correction, this pattern should be considered preliminary rather than confirmed.This pattern occurred under the same condition (DEO) where MSE group differences were most pronounced. However, these are distinct statistical properties, mean MSE differences versus a non-significant correlation trend, and should not be assumed to share a mechanism. DEO was not among the conditions selected for *a priori* COP comparisons (DEC, REC, LEC), and the group main effect was not significant for Area_95, AP_Vel_SD, or LFS. Replication in a larger sample is needed before this pattern can be interpreted further.

Current research on GJH balance often relies on traditional and isolated analytical parameters. For instance, Juul-Kristensen et al. (2015) utilized a 6-D force platform (AMTI OR6-7–1000) to compare the static balance of 16 GJH girls with 11 non-GJH (NGJH) controls. Their protocol was limited to double-leg (eyes open/closed) and single-leg (eyes open) stances, using only traditional COP metrics. Their results showed a significantly longer COP path length in the GJH group than in the non-GJH controls during the double-leg eyes-closed task (1.64 vs. 1.37 m/min, p < 0.001), which aligns with our general conclusions. However, some discrepancies in specific COP indicators persist, likely due to their exclusively female sample. In a meta-analysis of 19 studies, Wang et al. (2025) reported greater ML and AP oscillations during single-leg eyes-closed tasks in hypermobile adults (P < 0.05), which is partially consistent with the present results. Regarding nonlinear dynamics, Galli et al. (2010) reported decreased entropy in patients with EDS-HT, reflecting a loss of automaticity and a rigid control strategy. By contrast, the present study observed an increase in MSE values among the GJH population. This discrepancy may reflect differences in disease severity, task challenge, and entropy calculation methods. Different from the patients with EDS-HT in their work, the individuals with GJH in the present study were young and community-dwelling, suggesting that their neuromuscular compensatory capacity may be relatively preserved. Future studies should compare entropy measures across GJH, EDS-HT, and healthy populations under standardized protocols.

### 4.5 Limitations and future directions

Several limitations of this study should be acknowledged. First, this study only measured COP-derived variables and MSE. Proprioception, muscle activation, joint kinematics, and neuromuscular responses were not directly assessed. Therefore, the proposed compensatory mechanisms are based on prior literature rather than direct measurement. Future studies should integrate surface electromyography and joint kinematics to provide additional direct mechanistic evidence. Second, although the participants were instructed to stand naturally without adopting knee hyperextension or locking strategies, knee flexion angle was not standardized or objectively monitored during trials. COP displacement and MSE outcomes are sensitive to knee alignment. Given the high prevalence of knee hyperextension in the GJH group, systematic differences in habitual knee posture may have contributed to between-group differences in COP characteristics and should therefore be considered when interpreting alterations in postural control. Future studies should objectively verify knee posture, for example, through motion capture or goniometry, to control this potential confound. Third, the GJH and control groups were not perfectly matched for sex. Given known sex-related differences in joint hypermobility prevalence and postural control strategies, sex may have contributed to residual between-subject variability that was not explicitly modeled in the present analyses. On a more reassuring note, a formal trial-to-trial reliability analysis (Section 2.6) confirmed moderate-to-good reliability for the primary linear and nonlinear outcome measures underlying our key findings, supporting the robustness of the observed group differences against within-participant measurement variability. Fourth, while our analysis utilized high-fidelity time-series data from successfully completed trials to characterize nonlinear postural dynamics, the exact number of failed single-leg attempts (i.e., loss of balance before completing 30 s) was not recorded; trials were repeated in the field until a completed attempt was obtained. This is separate from the trial-quality exclusions noted in Section 2.5. The unrecorded failure rate remains a potential source of selection bias, as the final dataset may preferentially represent successful attempts.

## 5 Conclusion

This study identifies condition-specific COP patterns and task-dependent MSE patterns in young adults with GJH under varying postural constraints. Under the left-leg eyes-closed condition, the GJH group showed selected differences in mediolateral sway, while the complexity advantage observed during simpler tasks showed a descriptive pattern of attenuation as postural and sensory constraints increased. These findings suggest that postural-control characteristics in GJH vary across stance and visual conditions, with linear and nonlinear measures revealing distinct, condition-dependent patterns of group difference. Combining COP parameters with MSE analysis provided complementary insights that neither measure alone could capture. These findings should be regarded as hypothesis-generating regarding underlying neuromuscular mechanisms, as EMG, joint kinematics, proprioceptive testing were not assessed in this study.

## Data Availability

The original contributions presented in the study are included in the article/Supplementary Material, further inquiries can be directed to the corresponding author.
