# Supplementary material for: Condition-specific mediolateral sway and task-dependent postural complexity in individuals with generalized joint hypermobility
Source: Front Bioeng Biotechnol. 2026 Sep 21;14:1911414. doi: 10.3389/fbioe.2026.1911414 (PMC13634963; doi:10.3389/fbioe.2026.1911414)
Supplement: Supplementary file 1 [file Table1.docx]

Supplementary Material

Appendix 1 Post-Hoc Pairwise Comparisons for the Main Effect of Task Condition on Linear COP Parameters.

| **Parameters** | **Contrast** | ***T*** | ***DF*** | ***mean-diff*** | ***P*** | ***Pcorr*** | ***Cohen's d*** | ***Effect Size_CI*** |
| --- | --- | --- | --- | --- | --- | --- | --- | --- |
| TPL (mm) | DEO-DEC | -2.85 | 53 | -33.52 | <0.01 | 0.09 | -0.39 | [-0.664, -0.111] |
|  | DEO-REO | -19.31 | 53 | -596.22 | <0.001 | <0.001*** | -2.63 | [-3.191, -2.065] |
|  | DEO-REC | -20.40 | 53 | -1734.79 | <0.001 | <0.001*** | -2.78 | [-3.364, -2.189] |
|  | DEO-LEO | -19.33 | 53 | -656.11 | <0.001 | <0.001*** | -2.63 | [-3.193, -2.067] |
|  | DEO-LEC | -15.42 | 53 | -1966.87 | <0.001 | <0.001*** | -2.10 | [-2.575, -1.621] |
|  | DEC-REO | -17.42 | 53 | -562.70 | <0.001 | <0.001*** | -2.37 | [-2.891, -1.850] |
|  | DEC-REC | -20.37 | 53 | -1701.27 | <0.001 | <0.001*** | -2.77 | [-3.358, -2.185] |
|  | DEC-LEO | -18.38 | 53 | -622.59 | <0.001 | <0.001*** | -2.50 | [-3.043, -1.959] |
|  | DEC-LEC | -15.25 | 53 | -1933.35 | <0.001 | <0.001*** | -2.07 | [-2.548, -1.601] |
|  | REO-REC | -13.81 | 53 | -1138.57 | <0.001 | <0.001*** | -1.88 | [-2.323, -1.436] |
|  | REO-LEO | -1.87 | 53 | -59.89 | 0.07 | 1.00 | -0.25 | [-0.525, 0.017] |
|  | REO-LEC | -11.12 | 53 | -1370.65 | <0.001 | <0.001*** | -1.51 | [-1.903, -1.122] |
|  | REC-LEO | 15.38 | 53 | 1078.69 | <0.001 | <0.001*** | 2.09 | [1.617, 2.569] |
|  | REC-LEC | -2.70 | 53 | -232.07 | <0.01 | 0.14 | -0.37 | [-0.643, -0.092] |
|  | LEO-LEC | -11.49 | 53 | -1310.76 | <0.001 | <0.001*** | -1.56 | [-1.961, -1.166] |
| AP_PL (mm) | DEO-DEC | -4.44 | 53 | -37.69 | <0.001 | <0.001*** | -0.60 | [-0.894, -0.314] |
|  | DEO-REO | -16.51 | 53 | -357.63 | <0.001 | <0.001*** | -2.25 | [-2.748, -1.746] |
|  | DEO-REC | -17.16 | 53 | -1049.76 | <0.001 | <0.001*** | -2.34 | [-2.850, -1.820] |
|  | DEO-LEO | -15.83 | 53 | -398.65 | <0.001 | <0.001*** | -2.15 | [-2.640, -1.668] |
|  | DEO-LEC | -11.84 | 53 | -1225.00 | <0.001 | <0.001*** | -1.61 | [-2.016, -1.207] |
|  | DEC-REO | -14.12 | 53 | -319.94 | <0.001 | <0.001*** | -1.92 | [-2.371, -1.471] |
|  | DEC-REC | -17.13 | 53 | -1012.07 | <0.001 | <0.001*** | -2.33 | [-2.845, -1.817] |
|  | DEC-LEO | -14.62 | 53 | -360.96 | <0.001 | <0.001*** | -1.99 | [-2.449, -1.529] |
|  | DEC-LEC | -11.54 | 53 | -1187.31 | <0.001 | <0.001*** | -1.57 | [-1.969, -1.172] |
|  | REO-REC | -11.79 | 53 | -692.13 | <0.001 | <0.001*** | -1.60 | [-2.008, -1.201] |
|  | REO-LEO | -1.68 | 53 | -41.02 | 0.10 | 1.00 | -0.23 | [-0.498, 0.042] |
|  | REO-LEC | -8.51 | 53 | -867.37 | <0.001 | <0.001*** | -1.16 | [-1.503, -0.813] |
|  | REC-LEO | 13.04 | 53 | 651.11 | <0.001 | <0.001*** | 1.77 | [1.346, 2.202] |
|  | REC-LEC | -2.22 | 53 | -175.24 | 0.03 | 0.46 | -0.30 | [-0.575, -0.029] |
|  | LEO-LEC | -8.67 | 53 | -826.35 | <0.001 | <0.001*** | -1.18 | [-1.528, -0.833] |
| ML_PL (mm) | DEO-DEC | -0.05 | 53 | -0.35 | 0.96 | 1.00 | -0.01 | [-0.273, 0.261] |
|  | DEO-REO | -19.45 | 53 | -406.83 | <0.001 | <0.001*** | -2.65 | [-3.213, -2.081] |
|  | DEO-REC | -22.34 | 53 | -1160.35 | <0.001 | <0.001*** | -3.04 | [-3.672, -2.408] |
|  | DEO-LEO | -21.21 | 53 | -441.35 | <0.001 | <0.001*** | -2.89 | [-3.493, -2.281] |
|  | DEO-LEC | -19.37 | 53 | -1271.46 | <0.001 | <0.001*** | -2.64 | [-3.200, -2.072] |
|  | DEC-REO | -19.29 | 53 | -406.48 | <0.001 | <0.001*** | -2.62 | [-3.187, -2.063] |
|  | DEC-REC | -22.36 | 53 | -1160.00 | <0.001 | <0.001*** | -3.04 | [-3.675, -2.409] |
|  | DEC-LEO | -20.80 | 53 | -441.00 | <0.001 | <0.001*** | -2.83 | [-3.427, -2.233] |
|  | DEC-LEC | -19.40 | 53 | -1271.11 | <0.001 | <0.001*** | -2.64 | [-3.205, -2.076] |
|  | REO-REC | -15.10 | 53 | -753.52 | <0.001 | <0.001*** | -2.05 | [-2.525, -1.584] |
|  | REO-LEO | -1.71 | 53 | -34.52 | 0.09 | 1.00 | -0.23 | [-0.503, 0.037] |
|  | REO-LEC | -13.97 | 53 | -864.63 | <0.001 | <0.001*** | -1.90 | [-2.348, -1.454] |
|  | REC-LEO | 16.16 | 53 | 719.00 | <0.001 | <0.001*** | 2.20 | [1.706, 2.692] |
|  | REC-LEC | -2.66 | 53 | -111.11 | 0.01 | 0.15 | -0.36 | [-0.638, -0.087] |
|  | LEO-LEC | -14.97 | 53 | -830.11 | <0.001 | <0.001*** | -2.04 | [-2.504, -1.569] |
| MV (mm/s) | DEO-DEC | 0.65 | 53 | 2.43 | 0.52 | 1.00 | 0.09 | [-0.179, 0.356] |
|  | DEO-REO | -4.65 | 53 | -19.40 | <0.001 | <0.001*** | -0.63 | [-0.924, -0.340] |
|  | DEO-REC | -12.05 | 53 | -64.64 | <0.001 | <0.001*** | -1.64 | [-2.049, -1.232] |
|  | DEO-LEO | -5.21 | 53 | -21.77 | <0.001 | <0.001*** | -0.71 | [-1.008, -0.411] |
|  | DEO-LEC | -11.34 | 53 | -73.27 | <0.001 | <0.001*** | -1.54 | [-1.937, -1.148] |
|  | DEC-REO | -17.90 | 53 | -21.82 | <0.001 | <0.001*** | -2.44 | [-2.967, -1.904] |
|  | DEC-REC | -20.16 | 53 | -67.07 | <0.001 | <0.001*** | -2.74 | [-3.325, -2.161] |
|  | DEC-LEO | -18.87 | 53 | -24.19 | <0.001 | <0.001*** | -2.57 | [-3.121, -2.015] |
|  | DEC-LEC | -15.57 | 53 | -75.69 | <0.001 | <0.001*** | -2.12 | [-2.599, -1.638] |
|  | REO-REC | -14.05 | 53 | -45.25 | <0.001 | <0.001*** | -1.91 | [-2.360, -1.463] |
|  | REO-LEO | -1.92 | 53 | -2.37 | 0.06 | 0.91 | -0.26 | [-0.532, 0.010] |
|  | REO-LEC | -11.49 | 53 | -53.87 | <0.001 | <0.001*** | -1.56 | [-1.962, -1.166] |
|  | REC-LEO | 15.48 | 53 | 42.88 | <0.001 | <0.001*** | 2.11 | [1.629, 2.586] |
|  | REC-LEC | -2.57 | 53 | -8.62 | 0.01 | 0.20 | -0.35 | [-0.624, -0.075] |
|  | LEO-LEC | -11.83 | 53 | -51.50 | <0.001 | <0.001*** | -1.61 | [-2.014, -1.206] |
| AP_Vel_SD (mm/s) | DEO-DEC | -4.06 | 53 | -1.39 | <0.001 | <0.001*** | -0.55 | [-0.839, -0.266] |
|  | DEO-REO | -16.00 | 53 | -11.92 | <0.001 | <0.001*** | -2.18 | [-2.666, -1.687] |
|  | DEO-REC | -10.15 | 53 | -43.90 | <0.001 | <0.001*** | -1.38 | [-1.754, -1.008] |
|  | DEO-LEO | -14.34 | 53 | -13.63 | <0.001 | <0.001*** | -1.95 | [-2.406, -1.497] |
|  | DEO-LEC | -9.25 | 53 | -50.97 | <0.001 | <0.001*** | -1.26 | [-1.616, -0.902] |
|  | DEC-REO | -13.22 | 53 | -10.53 | <0.001 | <0.001*** | -1.80 | [-2.230, -1.367] |
|  | DEC-REC | -9.83 | 53 | -42.52 | <0.001 | <0.001*** | -1.34 | [-1.705, -0.970] |
|  | DEC-LEO | -12.93 | 53 | -12.24 | <0.001 | <0.001*** | -1.76 | [-2.186, -1.334] |
|  | DEC-LEC | -8.99 | 53 | -49.58 | <0.001 | <0.001*** | -1.22 | [-1.577, -0.871] |
|  | REO-REC | -7.50 | 53 | -31.99 | <0.001 | <0.001*** | -1.02 | [-1.350, -0.692] |
|  | REO-LEO | -1.82 | 53 | -1.71 | 0.07 | 1.00 | -0.25 | [-0.519, 0.023] |
|  | REO-LEC | -7.23 | 53 | -39.05 | <0.001 | <0.001*** | -0.98 | [-1.309, -0.659] |
|  | REC-LEO | 7.43 | 53 | 30.28 | <0.001 | <0.001*** | 1.01 | [0.683, 1.339] |
|  | REC-LEC | -1.70 | 53 | -7.06 | 0.09 | 1.00 | -0.23 | [-0.502, 0.038] |
|  | LEO-LEC | -7.09 | 53 | -37.34 | <0.001 | <0.001*** | -0.96 | [-1.288, -0.642] |
| ML_Vel_SD (mm/s) | DEO-DEC | 0.48 | 53 | 0.16 | 0.64 | 1.00 | 0.06 | [-0.202, 0.332] |
|  | DEO-REO | -19.48 | 53 | -14.31 | <0.001 | <0.001*** | -2.65 | [-3.217, -2.084] |
|  | DEO-REC | -9.41 | 53 | -47.68 | <0.001 | <0.001*** | -1.28 | [-1.640, -0.920] |
|  | DEO-LEO | -20.36 | 53 | -15.96 | <0.001 | <0.001*** | -2.77 | [-3.358, -2.184] |
|  | DEO-LEC | -12.06 | 53 | -51.02 | <0.001 | <0.001*** | -1.64 | [-2.050, -1.233] |
|  | DEC-REO | -20.20 | 53 | -14.48 | <0.001 | <0.001*** | -2.75 | [-3.332, -2.166] |
|  | DEC-REC | -9.39 | 53 | -47.85 | <0.001 | <0.001*** | -1.28 | [-1.638, -0.919] |
|  | DEC-LEO | -20.84 | 53 | -16.12 | <0.001 | <0.001*** | -2.84 | [-3.433, -2.238] |
|  | DEC-LEC | -12.16 | 53 | -51.18 | <0.001 | <0.001*** | -1.65 | [-2.065, -1.244] |
|  | REO-REC | -6.56 | 53 | -33.37 | <0.001 | <0.001*** | -0.89 | [-1.209, -0.578] |
|  | REO-LEO | -2.37 | 53 | -1.64 | 0.02 | 0.33 | -0.32 | [-0.595, -0.048] |
|  | REO-LEC | -8.95 | 53 | -36.71 | <0.001 | <0.001*** | -1.22 | [-1.570, -0.866] |
|  | REC-LEO | 6.37 | 53 | 31.73 | <0.001 | <0.001*** | 0.87 | [0.553, 1.179] |
|  | REC-LEC | -0.72 | 53 | -3.34 | 0.47 | 1.00 | -0.10 | [-0.365, 0.169] |
|  | LEO-LEC | -9.01 | 53 | -35.06 | <0.001 | <0.001*** | -1.23 | [-1.579, -0.873] |
| AP_Amp (mm) | DEO-DEC | -1.49 | 53 | -2.46 | 0.14 | 1.00 | -0.20 | [-0.473, 0.066] |
|  | DEO-REO | -8.58 | 53 | -15.56 | <0.001 | <0.001*** | -1.17 | [-1.513, -0.822] |
|  | DEO-REC | -10.48 | 53 | -50.81 | <0.001 | <0.001*** | -1.43 | [-1.805, -1.048] |
|  | DEO-LEO | -8.43 | 53 | -17.04 | <0.001 | <0.001*** | -1.15 | [-1.490, -0.803] |
|  | DEO-LEC | -10.39 | 53 | -57.56 | <0.001 | <0.001*** | -1.41 | [-1.791, -1.037] |
|  | DEC-REO | -7.78 | 53 | -13.10 | <0.001 | <0.001*** | -1.06 | [-1.392, -0.726] |
|  | DEC-REC | -10.77 | 53 | -48.34 | <0.001 | <0.001*** | -1.47 | [-1.849, -1.081] |
|  | DEC-LEO | -8.70 | 53 | -14.58 | <0.001 | <0.001*** | -1.18 | [-1.531, -0.836] |
|  | DEC-LEC | -10.20 | 53 | -55.10 | <0.001 | <0.001*** | -1.39 | [-1.762, -1.014] |
|  | REO-REC | -7.53 | 53 | -35.24 | <0.001 | <0.001*** | -1.02 | [-1.354, -0.695] |
|  | REO-LEO | -0.91 | 53 | -1.48 | 0.36 | 1.00 | -0.12 | [-0.392, 0.143] |
|  | REO-LEC | -8.06 | 53 | -42.00 | <0.001 | <0.001*** | -1.10 | [-1.434, -0.759] |
|  | REC-LEO | 7.80 | 53 | 33.77 | <0.001 | <0.001*** | 1.06 | [0.728, 1.395] |
|  | REC-LEC | -1.50 | 53 | -6.76 | 0.14 | 1.00 | -0.20 | [-0.473, 0.065] |
|  | LEO-LEC | -8.39 | 53 | -40.52 | <0.001 | <0.001*** | -1.14 | [-1.485, -0.799] |
| ML_Amp (mm) | DEO-DEC | 1.09 | 53 | 1.28 | 0.28 | 1.00 | 0.15 | [-0.120, 0.416] |
|  | DEO-REO | -11.43 | 53 | -14.92 | <0.001 | <0.001*** | -1.56 | [-1.952, -1.159] |
|  | DEO-REC | -7.25 | 53 | -46.59 | <0.001 | <0.001*** | -0.99 | [-1.311, -0.661] |
|  | DEO-LEO | -10.30 | 53 | -15.40 | <0.001 | <0.001*** | -1.40 | [-1.777, -1.026] |
|  | DEO-LEC | -7.98 | 53 | -49.14 | <0.001 | <0.001*** | -1.09 | [-1.422, -0.749] |
|  | DEC-REO | -16.83 | 53 | -16.19 | <0.001 | <0.001*** | -2.29 | [-2.797, -1.782] |
|  | DEC-REC | -7.49 | 53 | -47.87 | <0.001 | <0.001*** | -1.02 | [-1.348, -0.691] |
|  | DEC-LEO | -15.17 | 53 | -16.68 | <0.001 | <0.001*** | -2.06 | [-2.536, -1.593] |
|  | DEC-LEC | -8.39 | 53 | -50.42 | <0.001 | <0.001*** | -1.14 | [-1.485, -0.799] |
|  | REO-REC | -4.96 | 53 | -31.67 | <0.001 | <0.001*** | -0.68 | [-0.971, -0.380] |
|  | REO-LEO | -0.63 | 53 | -0.49 | 0.53 | 1.00 | -0.09 | [-0.353, 0.181] |
|  | REO-LEC | -5.83 | 53 | -34.23 | <0.001 | <0.001*** | -0.79 | [-1.099, -0.487] |
|  | REC-LEO | 5.01 | 53 | 31.19 | <0.001 | <0.001*** | 0.68 | [0.385, 0.977] |
|  | REC-LEC | -0.57 | 53 | -2.56 | 0.57 | 1.00 | -0.08 | [-0.345, 0.189] |
|  | LEO-LEC | -5.75 | 53 | -33.74 | <0.001 | <0.001*** | -0.78 | [-1.087, -0.478] |
| AP_SD (mm) | DEO-DEC | -0.37 | 53 | -0.16 | 0.71 | 1.00 | -0.05 | [-0.317, 0.217] |
|  | DEO-REO | -4.90 | 53 | -2.36 | <0.001 | <0.001*** | -0.67 | [-0.962, -0.372] |
|  | DEO-REC | -9.57 | 53 | -6.41 | <0.001 | <0.001*** | -1.30 | [-1.664, -0.939] |
|  | DEO-LEO | -4.82 | 53 | -2.48 | <0.001 | <0.001*** | -0.66 | [-0.949, -0.361] |
|  | DEO-LEC | -8.03 | 53 | -7.61 | <0.001 | <0.001*** | -1.09 | [-1.430, -0.755] |
|  | DEC-REO | -5.82 | 53 | -2.20 | <0.001 | <0.001*** | -0.79 | [-1.097, -0.486] |
|  | DEC-REC | -12.00 | 53 | -6.25 | <0.001 | <0.001*** | -1.63 | [-2.040, -1.226] |
|  | DEC-LEO | -6.18 | 53 | -2.32 | <0.001 | <0.001*** | -0.84 | [-1.152, -0.531] |
|  | DEC-LEC | -8.63 | 53 | -7.45 | <0.001 | <0.001*** | -1.17 | [-1.521, -0.828] |
|  | REO-REC | -8.35 | 53 | -4.05 | <0.001 | <0.001*** | -1.14 | [-1.479, -0.795] |
|  | REO-LEO | -0.39 | 53 | -0.12 | 0.70 | 1.00 | -0.05 | [-0.320, 0.214] |
|  | REO-LEC | -6.53 | 53 | -5.24 | <0.001 | <0.001*** | -0.89 | [-1.204, -0.574] |
|  | REC-LEO | 9.18 | 53 | 3.93 | <0.001 | <0.001*** | 1.25 | [0.893, 1.605] |
|  | REC-LEC | -2.12 | 53 | -1.20 | 0.04 | 0.58 | -0.29 | [-0.560, -0.016] |
|  | LEO-LEC | -7.00 | 53 | -5.12 | <0.001 | <0.001*** | -0.95 | [-1.274, -0.631] |
| ML_SD (mm) | DEO-DEC | 1.11 | 53 | 0.26 | 0.27 | 1.00 | 0.15 | [-0.117, 0.419] |
|  | DEO-REO | -10.90 | 53 | -2.84 | <0.001 | <0.001*** | -1.48 | [-1.870, -1.097] |
|  | DEO-REC | -10.76 | 53 | -9.38 | <0.001 | <0.001*** | -1.46 | [-1.849, -1.081] |
|  | DEO-LEO | -10.55 | 53 | -2.94 | <0.001 | <0.001*** | -1.44 | [-1.815, -1.055] |
|  | DEO-LEC | -8.64 | 53 | -10.40 | <0.001 | <0.001*** | -1.18 | [-1.523, -0.829] |
|  | DEC-REO | -16.02 | 53 | -3.10 | <0.001 | <0.001*** | -2.18 | [-2.670, -1.690] |
|  | DEC-REC | -10.99 | 53 | -9.64 | <0.001 | <0.001*** | -1.49 | [-1.883, -1.107] |
|  | DEC-LEO | -16.14 | 53 | -3.19 | <0.001 | <0.001*** | -2.20 | [-2.689, -1.704] |
|  | DEC-LEC | -8.96 | 53 | -10.66 | <0.001 | <0.001*** | -1.22 | [-1.572, -0.867] |
|  | REO-REC | -7.68 | 53 | -6.54 | <0.001 | <0.001*** | -1.05 | [-1.377, -0.714] |
|  | REO-LEO | -0.65 | 53 | -0.10 | 0.52 | 1.00 | -0.09 | [-0.355, 0.179] |
|  | REO-LEC | -6.56 | 53 | -7.56 | <0.001 | <0.001*** | -0.89 | [-1.208, -0.578] |
|  | REC-LEO | 7.81 | 53 | 6.44 | <0.001 | <0.001*** | 1.06 | [0.729, 1.397] |
|  | REC-LEC | -1.16 | 53 | -1.02 | 0.25 | 1.00 | -0.16 | [-0.426, 0.111] |
|  | LEO-LEC | -6.42 | 53 | -7.46 | <0.001 | <0.001*** | -0.87 | [-1.187, -0.560] |
| AP_SI (%) | DEO-DEC | -4.75 | 53 | -5.45 | <0.001 | <0.001*** | -0.65 | [-0.940, -0.354] |
|  | DEO-REO | 3.45 | 53 | 4.33 | <0.01 | 0.02* | 0.47 | [0.189, 0.751] |
|  | DEO-REC | 4.60 | 53 | 5.26 | <0.001 | <0.001*** | 0.63 | [0.334, 0.918] |
|  | DEO-LEO | 3.62 | 53 | 4.17 | <0.01 | 0.01* | 0.49 | [0.210, 0.775] |
|  | DEO-LEC | 3.85 | 53 | 4.47 | <0.001 | <0.001*** | 0.52 | [0.239, 0.808] |
|  | DEC-REO | 10.30 | 53 | 9.78 | <0.001 | <0.001*** | 1.40 | [1.026, 1.777] |
|  | DEC-REC | 11.71 | 53 | 10.71 | <0.001 | <0.001*** | 1.59 | [1.192, 1.996] |
|  | DEC-LEO | 10.16 | 53 | 9.62 | <0.001 | <0.001*** | 1.38 | [1.009, 1.755] |
|  | DEC-LEC | 9.56 | 53 | 9.92 | <0.001 | <0.001*** | 1.30 | [0.939, 1.663] |
|  | REO-REC | 1.51 | 53 | 0.93 | 0.14 | 1.00 | 0.20 | [-0.065, 0.474] |
|  | REO-LEO | -0.22 | 53 | -0.16 | 0.83 | 1.00 | -0.03 | [-0.296, 0.237] |
|  | REO-LEC | 0.16 | 53 | 0.14 | 0.88 | 1.00 | 0.02 | [-0.245, 0.288] |
|  | REC-LEO | -1.57 | 53 | -1.09 | 0.12 | 1.00 | -0.21 | [-0.483, 0.057] |
|  | REC-LEC | -1.02 | 53 | -0.79 | 0.31 | 1.00 | -0.14 | [-0.407, 0.129] |
|  | LEO-LEC | 0.49 | 53 | 0.30 | 0.63 | 1.00 | 0.07 | [-0.201, 0.333] |
| ML_SI (%) | DEO-DEC | 4.75 | 53 | 5.45 | <0.001 | <0.001*** | 0.78 | [0.474, 1.083] |
|  | DEO-REO | -6.07 | 53 | -5.18 | <0.001 | <0.001*** | -0.83 | [-1.135, -0.518] |
|  | DEO-REC | -7.36 | 53 | -6.11 | <0.001 | <0.001*** | -1.00 | [-1.328, -0.675] |
|  | DEO-LEO | -6.94 | 53 | -5.02 | <0.001 | <0.001*** | -0.94 | [-1.266, -0.624] |
|  | DEO-LEC | -6.59 | 53 | -5.32 | <0.001 | <0.001*** | -0.90 | [-1.212, -0.581] |
|  | DEC-REO | -10.30 | 53 | -9.78 | <0.001 | <0.001*** | -1.40 | [-1.777, -1.026] |
|  | DEC-REC | -11.71 | 53 | -10.71 | <0.001 | <0.001*** | -1.59 | [-1.996, -1.192] |
|  | DEC-LEO | -10.16 | 53 | -9.62 | <0.001 | <0.001*** | -1.38 | [-1.755, -1.009] |
|  | DEC-LEC | -9.56 | 53 | -9.92 | <0.001 | <0.001*** | -1.30 | [-1.663, -0.939] |
|  | REO-REC | -1.51 | 53 | -0.93 | 0.14 | 1.00 | -0.20 | [-0.474, 0.065] |
|  | REO-LEO | 0.22 | 53 | 0.16 | 0.83 | 1.00 | 0.03 | [-0.237, 0.296] |
|  | REO-LEC | -0.16 | 53 | -0.14 | 0.88 | 1.00 | -0.02 | [-0.288, 0.245] |
|  | REC-LEO | 1.57 | 53 | 1.09 | 0.12 | 1.00 | 0.21 | [-0.057, 0.483] |
|  | REC-LEC | 1.02 | 53 | 0.79 | 0.31 | 1.00 | 0.14 | [-0.129, 0.407] |
|  | LEO-LEC | -0.49 | 53 | -0.30 | 0.63 | 1.00 | -0.07 | [-0.333, 0.201] |
| AP_RMS (mm) | DEO-DEC | 0.50 | 53 | 0.41 | 0.62 | 1.00 | 0.07 | [-0.199, 0.335] |
|  | DEO-REO | -2.01 | 53 | -1.80 | 0.05 | 0.74 | -0.27 | [-0.545, -0.002] |
|  | DEO-REC | -5.66 | 53 | -5.84 | <0.001 | <0.001*** | -0.77 | [-1.075, -0.467] |
|  | DEO-LEO | -2.04 | 53 | -1.92 | 0.05 | 0.70 | -0.28 | [-0.549, -0.005] |
|  | DEO-LEC | -5.64 | 53 | -7.04 | <0.001 | <0.001*** | -0.77 | [-1.071, -0.464] |
|  | DEC-REO | -5.82 | 53 | -2.20 | <0.001 | <0.001*** | -0.79 | [-1.097, -0.486] |
|  | DEC-REC | -12.00 | 53 | -6.25 | <0.001 | <0.001*** | -1.63 | [-2.040, -1.226] |
|  | DEC-LEO | -6.18 | 53 | -2.32 | <0.001 | <0.001*** | -0.84 | [-1.152, -0.531] |
|  | DEC-LEC | -8.63 | 53 | -7.45 | <0.001 | <0.001*** | -1.17 | [-1.521, -0.828] |
|  | REO-REC | -8.35 | 53 | -4.05 | <0.001 | <0.001*** | -1.14 | [-1.479, -0.795] |
|  | REO-LEO | -0.39 | 53 | -0.12 | 0.70 | 1.00 | -0.05 | [-0.320, 0.214] |
|  | REO-LEC | -6.53 | 53 | -5.24 | <0.001 | <0.001*** | -0.89 | [-1.204, -0.574] |
|  | REC-LEO | 9.18 | 53 | 3.93 | <0.001 | <0.001*** | 1.25 | [0.893, 1.605] |
|  | REC-LEC | -2.12 | 53 | -1.20 | 0.04 | 0.58 | -0.29 | [-0.560, -0.016] |
|  | LEO-LEC | -7.00 | 53 | -5.12 | <0.001 | <0.001*** | -0.95 | [-1.274, -0.631] |
| ML_RMS (mm) | DEO-DEC | 1.11 | 53 | 0.26 | 0.27 | 1.00 | 0.15 | [-0.117, 0.419] |
|  | DEO-REO | -10.90 | 53 | -2.84 | <0.001 | <0.001*** | -1.48 | [-1.870, -1.097] |
|  | DEO-REC | -10.76 | 53 | -9.38 | <0.001 | <0.001*** | -1.46 | [-1.849, -1.081] |
|  | DEO-LEO | -10.55 | 53 | -2.94 | <0.001 | <0.001*** | -1.44 | [-1.815, -1.055] |
|  | DEO-LEC | -8.64 | 53 | -10.40 | <0.001 | <0.001*** | -1.18 | [-1.523, -0.829] |
|  | DEC-REO | -16.02 | 53 | -3.10 | <0.001 | <0.001*** | -2.18 | [-2.670, -1.690] |
|  | DEC-REC | -10.99 | 53 | -9.64 | <0.001 | <0.001*** | -1.49 | [-1.883, -1.107] |
|  | DEC-LEO | -16.14 | 53 | -3.19 | <0.001 | <0.001*** | -2.20 | [-2.689, -1.704] |
|  | DEC-LEC | -8.96 | 53 | -10.66 | <0.001 | <0.001*** | -1.22 | [-1.572, -0.867] |
|  | REO-REC | -7.68 | 53 | -6.54 | <0.001 | <0.001*** | -1.05 | [-1.377, -0.714] |
|  | REO-LEO | -0.65 | 53 | -0.10 | 0.52 | 1.00 | -0.09 | [-0.355, 0.179] |
|  | REO-LEC | -6.56 | 53 | -7.56 | <0.001 | <0.001*** | -0.89 | [-1.208, -0.578] |
|  | REC-LEO | 7.81 | 53 | 6.44 | <0.001 | <0.001*** | 1.06 | [0.729, 1.397] |
|  | REC-LEC | -1.16 | 53 | -1.02 | 0.25 | 1.00 | -0.16 | [-0.426, 0.111] |
|  | LEO-LEC | -6.42 | 53 | -7.46 | <0.001 | <0.001*** | -0.87 | [-1.187, -0.560] |
| Area_95 (mm²) | DEO-DEC | 0.68 | 53 | 43.50 | 0.50 | 1.00 | 0.09 | [-0.175, 0.359] |
|  | DEO-REO | -7.12 | 53 | -470.79 | <0.001 | <0.001*** | -0.97 | [-1.292, -0.645] |
|  | DEO-REC | -6.51 | 53 | -2571.59 | <0.001 | <0.001*** | -0.89 | [-1.201, -0.571] |
|  | DEO-LEO | -6.73 | 53 | -500.83 | <0.001 | <0.001*** | -0.92 | [-1.233, -0.598] |
|  | DEO-LEC | -3.87 | 53 | -3626.27 | <0.001 | <0.001*** | -0.53 | [-0.811, -0.242] |
|  | DEC-REO | -13.12 | 53 | -514.29 | <0.001 | <0.001*** | -1.79 | [-2.216, -1.356] |
|  | DEC-REC | -6.66 | 53 | -2615.09 | <0.001 | <0.001*** | -0.91 | [-1.223, -0.589] |
|  | DEC-LEO | -13.67 | 53 | -544.33 | <0.001 | <0.001*** | -1.86 | [-2.302, -1.420] |
|  | DEC-LEC | -3.93 | 53 | -3669.77 | <0.001 | <0.001*** | -0.53 | [-0.820, -0.249] |
|  | REO-REC | -5.34 | 53 | -2100.80 | <0.001 | <0.001*** | -0.73 | [-1.027, -0.427] |
|  | REO-LEO | -0.78 | 53 | -30.04 | 0.44 | 1.00 | -0.11 | [-0.374, 0.161] |
|  | REO-LEC | -3.39 | 53 | -3155.48 | <0.01 | 0.02* | -0.46 | [-0.741, -0.180] |
|  | REC-LEO | 5.40 | 53 | 2070.76 | <0.001 | <0.001*** | 0.74 | [0.435, 1.036] |
|  | REC-LEC | -1.59 | 53 | -1054.69 | 0.12 | 1.00 | -0.22 | [-0.486, 0.053] |
|  | LEO-LEC | -3.36 | 53 | -3125.44 | <0.01 | 0.02* | -0.46 | [-0.738, -0.177] |
| CHA (mm²) | DEO-DEC | 0.78 | 53 | 40.43 | 0.44 | 1.00 | 0.11 | [-0.161, 0.374] |
|  | DEO-REO | -8.70 | 53 | -500.68 | <0.001 | <0.001*** | -1.18 | [-1.531, -0.836] |
|  | DEO-REC | -3.54 | 53 | -3684.70 | 0.00 | 0.01* | -0.48 | [-0.763, -0.200] |
|  | DEO-LEO | -8.02 | 53 | -563.63 | <0.001 | <0.001*** | -1.09 | [-1.428, -0.755] |
|  | DEO-LEC | -4.26 | 53 | -4221.78 | <0.001 | <0.001*** | -0.58 | [-0.868, -0.291] |
|  | DEC-REO | -14.46 | 53 | -541.11 | <0.001 | <0.001*** | -1.97 | [-2.425, -1.511] |
|  | DEC-REC | -3.58 | 53 | -3725.13 | <0.01 | 0.01* | -0.49 | [-0.769, -0.205] |
|  | DEC-LEO | -13.23 | 53 | -604.06 | <0.001 | <0.001*** | -1.80 | [-2.232, -1.368] |
|  | DEC-LEC | -4.32 | 53 | -4262.20 | <0.001 | <0.001*** | -0.59 | [-0.876, -0.298] |
|  | REO-REC | -3.05 | 53 | -3184.02 | <0.01 | 0.05 | -0.42 | [-0.693, -0.137] |
|  | REO-LEO | -1.46 | 53 | -62.94 | 0.15 | 1.00 | -0.20 | [-0.469, 0.070] |
|  | REO-LEC | -3.78 | 53 | -3721.09 | <0.001 | <0.01** | -0.51 | [-0.798, -0.230] |
|  | REC-LEO | 3.01 | 53 | 3121.07 | <0.01 | 0.06 | 0.41 | [0.132, 0.688] |
|  | REC-LEC | -1.10 | 53 | -537.07 | 0.27 | 1.00 | -0.15 | [-0.419, 0.118] |
|  | LEO-LEC | -3.73 | 53 | -3658.15 | <0.001 | <0.01** | -0.51 | [-0.791, -0.224] |

Data represent pairwise comparisons for the main effect of task condition across all subjects. (N = 54), Pcorr was adjusted using the Bonferroni correction for multiple comparisons. Abbreviations: Total Path Length (TPL), Antero-posterior Path Length (AP_PL), Medio-lateral Path Length (ML_PL), Antero-posterior Displacement Amplitude (AP_Amp), Medio-lateral Displacement Amplitude (ML_Amp), Antero-posterior Sway Index (AP_SI), Medio-lateral Sway Index (ML_SI), Antero-posterior Displacement Standard Deviation (AP_SD), Medio-lateral Displacement Standard Deviation (ML_SD), Total Mean Velocity (MV), Antero-posterior Velocity Standard Deviation (AP_Vel_SD), Medio-lateral Velocity Standard Deviation (ML_Vel_SD), Antero-posterior Root-Mean-Square Displacement (AP_RMS), Medio-lateral Root-Mean-Square Displacement (ML_RMS), 95% Confidence Ellipse Area (Area_95), Convex Hull Area (CHA);***, P < 0.001; **, P < 0.01;*, P < 0.05.P values are uncorrected for multiple comparisons. Pcorr values represent p-values adjusted for multiple comparisons using the Bonferroni correction. T: t-statistic; df: degrees of freedom (N - 1); Mean diff: mean difference between conditions; CI: 95% confidence interval of the mean difference.Statistical significance is determined based on Pcorr.

Appendix 2 Simple Effect Analysis of Multiscale Entropy (MSE) Between GJH and Healthy Controls Across Tasks and Scales.

| **Indicators** | **Condition** | **GJH** | **Healthy** | ***P*** | ***Pcorr*** | ***Cohen’s d*** | ***CI*** |
| --- | --- | --- | --- | --- | --- | --- | --- |
| AP_MSE_Sum | DEO | 15.32±1.22 | 12.60±3.93 | <0.01 | 0.01** | 0.95 | [0.386, 1.514] |
|  | DEC | 14.89±0.97 | 11.96±3.47 | <0.001 | <0.01** | 1.17 | [0.591, 1.749] |
|  | REO | 13.21±0.68 | 11.53±2.36 | <0.01 | <0.01** | 0.98 | [0.416, 1.549] |
|  | REC | 12.66±1.06 | 10.10±2.20 | <0.001 | <0.001*** | 1.50 | [0.894, 2.107] |
|  | LEO | 13.28±1.02 | 11.62±2.81 | <0.01 | 0.048* | 0.80 | [0.240, 1.351] |
|  | LEC | 11.71±1.81 | 10.72±2.43 | 0.10 | 0.59 | 0.46 | [-0.076, 1.006] |
| ML_MSE_Sum | DEO | 15.45±1.05 | 13.00±3.70 | <0.01 | 0.02* | 0.92 | [0.356, 1.480] |
|  | DEC | 15.80±0.64 | 12.24±4.05 | <0.001 | <0.01** | 1.25 | [0.666, 1.837] |
|  | REO | 12.79±1.01 | 11.29±2.34 | <0.01 | 0.03* | 0.85 | [0.289, 1.405] |
|  | REC | 11.39±0.79 | 10.07±1.71 | <0.01 | 0.01* | 1.00 | [0.436, 1.571] |
|  | LEO | 12.48±1.12 | 10.86±2.70 | <0.01 | 0.047* | 0.79 | [0.238, 1.348] |
|  | LEC | 11.21±0.69 | 10.32±1.63 | 0.02 | 0.09 | 0.72 | [0.164, 1.267] |
| AP_MSE_Scale1 | DEO | 1.43±0.12 | 1.13±0.43 | <0.01 | 0.01* | 0.96 | [0.395, 1.524] |
|  | DEC | 1.35±0.19 | 1.01±0.36 | <0.001 | <0.001*** | 1.21 | [0.626, 1.790] |
|  | REO | 0.83±0.15 | 0.73±0.23 | 0.07 | 0.42 | 0.51 | [-0.031, 1.055] |
|  | REC | 0.73±0.07 | 0.59±0.15 | <0.001 | <0.001*** | 1.21 | [0.631, 1.796] |
|  | LEO | 0.84±0.20 | 0.80±0.31 | 0.54 | 1.00 | 0.17 | [-0.362, 0.707] |
|  | LEC | 0.70±0.09 | 0.62±0.14 | 0.03 | 0.15 | 0.64 | [0.093, 1.189] |
| AP_MSE_Scale2 | DEO | 1.96±0.20 | 1.58±0.55 | <0.01 | 0.01* | 0.93 | [0.367, 1.493] |
|  | DEC | 1.84±0.19 | 1.46±0.47 | <0.01 | <0.01** | 1.08 | [0.504, 1.649] |
|  | REO | 1.35±0.13 | 1.16±0.28 | <0.01 | 0.02* | 0.89 | [0.333, 1.454] |
|  | REC | 1.24±0.10 | 0.96±0.24 | <0.001 | <0.001*** | 1.54 | [0.927, 2.147] |
|  | LEO | 1.33±0.18 | 1.19±0.34 | 0.06 | 0.36 | 0.54 | [-0.002, 1.086] |
|  | LEC | 1.15±0.14 | 1.04±0.24 | 0.04 | 0.27 | 0.58 | [0.033, 1.123] |
| AP_MSE_Scale3 | DEO | 2.10±0.20 | 1.70±0.58 | <0.01 | 0.01* | 0.96 | [0.392, 1.521] |
|  | DEC | 2.00±0.16 | 1.59±0.50 | <0.001 | <0.01** | 1.15 | [0.568, 1.723] |
|  | REO | 1.65±0.13 | 1.40±0.33 | <0.01 | 0.01* | 1.01 | [0.445, 1.581] |
|  | REC | 1.56±0.13 | 1.19±0.30 | <0.001 | <0.001*** | 1.59 | [0.974, 2.204] |
|  | LEO | 1.64±0.17 | 1.43±0.40 | 0.02 | 0.10 | 0.71 | [0.160, 1.262] |
|  | LEC | 1.43±0.21 | 1.28±0.31 | 0.04 | 0.24 | 0.58 | [0.039, 1.130] |
| AP_MSE_Scale4 | DEO | 2.07±0.18 | 1.70±0.55 | <0.01 | 0.02* | 0.92 | [0.361, 1.486] |
|  | DEC | 2.01±0.14 | 1.61±0.48 | <0.001 | <0.01** | 1.16 | [0.582, 1.740] |
|  | REO | 1.80±0.11 | 1.53±0.34 | <0.01 | <0.01** | 1.07 | [0.502, 1.647] |
|  | REC | 1.73±0.15 | 1.34±0.33 | <0.001 | <0.001*** | 1.50 | [0.893, 2.107] |
|  | LEO | 1.81±0.15 | 1.55±0.41 | <0.01 | 0.03* | 0.85 | [0.288, 1.404] |
|  | LEC | 1.59±0.25 | 1.43±0.36 | 0.06 | 0.35 | 0.54 | [-0.007, 1.081] |
| AP_MSE_Scale5 | DEO | 2.02±0.19 | 1.65±0.51 | <0.01 | 0.01* | 0.96 | [0.391, 1.520] |
|  | DEC | 1.99±0.12 | 1.60±0.47 | <0.001 | <0.01** | 1.15 | [0.569, 1.724] |
|  | REO | 1.87±0.10 | 1.61±0.35 | <0.01 | <0.01** | 1.06 | [0.490, 1.633] |
|  | REC | 1.80±0.15 | 1.42±0.34 | <0.001 | <0.001*** | 1.47 | [0.867, 2.075] |
|  | LEO | 1.88±0.14 | 1.61±0.40 | <0.01 | 0.01* | 0.95 | [0.383, 1.511] |
|  | LEC | 1.68±0.28 | 1.51±0.38 | 0.07 | 0.41 | 0.52 | [-0.028, 1.058] |
| AP_MSE_Scale6 | DEO | 1.98±0.18 | 1.64±0.49 | <0.01 | 0.01* | 0.93 | [0.367, 1.493] |
|  | DEC | 1.95±0.12 | 1.59±0.44 | <0.001 | <0.01** | 1.14 | [0.566, 1.720] |
|  | REO | 1.90±0.09 | 1.67±0.34 | <0.01 | 0.01* | 0.96 | [0.392, 1.521] |
|  | REC | 1.87±0.20 | 1.50±0.33 | <0.001 | <0.001*** | 1.39 | [0.794, 1.988] |
|  | LEO | 1.91±0.12 | 1.66±0.38 | <0.01 | 0.02* | 0.90 | [0.337, 1.458] |
|  | LEC | 1.72±0.30 | 1.58±0.38 | 0.16 | 0.95 | 0.39 | [-0.146, 0.932] |
| AP_MSE_Scale7 | DEO | 1.90±0.16 | 1.61±0.46 | <0.01 | 0.03* | 0.86 | [0.298, 1.415] |
|  | DEC | 1.90±0.15 | 1.56±0.40 | <0.001 | <0.01** | 1.14 | [0.560, 1.714] |
|  | REO | 1.90±0.09 | 1.69±0.30 | <0.01 | 0.01* | 0.95 | [0.388, 1.517] |
|  | REC | 1.87±0.18 | 1.54±0.31 | <0.001 | <0.001*** | 1.29 | [0.701, 1.878] |
|  | LEO | 1.95±0.12 | 1.69±0.35 | <0.01 | 0.01* | 1.00 | [0.434, 1.569] |
|  | LEC | 1.74±0.30 | 1.62±0.34 | 0.18 | 1.00 | 0.37 | [-0.168, 0.910] |
| AP_MSE_Scale8 | DEO | 1.85±0.15 | 1.58±0.44 | <0.01 | 0.03* | 0.84 | [0.282, 1.398] |
|  | DEC | 1.83±0.13 | 1.54±0.39 | <0.01 | <0.01** | 1.05 | [0.476, 1.617] |
|  | REO | 1.90±0.10 | 1.74±0.28 | 0.01 | 0.06 | 0.77 | [0.216, 1.324] |
|  | REC | 1.86±0.20 | 1.55±0.27 | <0.001 | <0.001*** | 1.32 | [0.726, 1.908] |
|  | LEO | 1.91±0.10 | 1.70±0.32 | <0.01 | 0.02* | 0.90 | [0.336, 1.458] |
|  | LEC | 1.70±0.31 | 1.65±0.37 | 0.53 | 1.00 | 0.17 | [-0.361, 0.708] |
| ML_MSE_Scale1 | DEO | 1.54±0.13 | 1.22±0.48 | <0.01 | 0.02* | 0.93 | [0.367, 1.492] |
|  | DEC | 1.54±0.16 | 1.12±0.49 | <0.001 | <0.01** | 1.17 | [0.591, 1.749] |
|  | REO | 0.90±0.23 | 0.75±0.30 | 0.047 | 0.28 | 0.56 | [0.017, 1.106] |
|  | REC | 0.68±0.09 | 0.56±0.11 | <0.001 | <0.001*** | 1.20 | [0.621, 1.784] |
|  | LEO | 0.87±0.21 | 0.73±0.31 | 0.06 | 0.38 | 0.53 | [-0.017, 1.069] |
|  | LEC | 0.64±0.02 | 0.57±0.10 | <0.01 | 0.02* | 0.92 | [0.354, 1.478] |
| ML_MSE_Scale2 | DEO | 2.14±0.16 | 1.71±0.60 | <0.01 | 0.01* | 1.00 | [0.433, 1.568] |
|  | DEC | 2.16±0.16 | 1.60±0.64 | <0.001 | <0.01** | 1.23 | [0.645, 1.812] |
|  | REO | 1.36±0.20 | 1.14±0.34 | <0.01 | 0.03* | 0.81 | [0.256, 1.369] |
|  | REC | 1.07±0.10 | 0.89±0.19 | <0.001 | <0.001*** | 1.18 | [0.600, 1.760] |
|  | LEO | 1.31±0.19 | 1.10±0.38 | 0.02 | 0.12 | 0.68 | [0.130, 1.230] |
|  | LEC | 1.02±0.06 | 0.90±0.18 | <0.01 | 0.02* | 0.89 | [0.333, 1.455] |
| ML_MSE_Scale3 | DEO | 2.20±0.15 | 1.80±0.58 | <0.01 | 0.01* | 0.98 | [0.410, 1.541] |
|  | DEC | 2.22±0.13 | 1.69±0.63 | <0.001 | <0.01** | 1.20 | [0.614, 1.777] |
|  | REO | 1.61±0.17 | 1.35±0.36 | <0.01 | 0.01* | 0.93 | [0.369, 1.495] |
|  | REC | 1.32±0.10 | 1.11±0.24 | <0.001 | <0.01** | 1.15 | [0.573, 1.728] |
|  | LEO | 1.55±0.17 | 1.30±0.40 | <0.01 | 0.04* | 0.81 | [0.252, 1.364] |
|  | LEC | 1.28±0.09 | 1.13±0.23 | <0.01 | 0.03* | 0.84 | [0.282, 1.397] |
| ML_MSE_Scale4 | DEO | 2.11±0.15 | 1.76±0.53 | <0.01 | 0.01* | 0.93 | [0.369, 1.495] |
|  | DEC | 2.17±0.11 | 1.67±0.58 | <0.001 | <0.001*** | 1.24 | [0.657, 1.827] |
|  | REO | 1.70±0.13 | 1.47±0.35 | <0.01 | 0.02* | 0.87 | [0.312, 1.431] |
|  | REC | 1.49±0.11 | 1.28±0.26 | <0.01 | <0.01** | 1.06 | [0.488, 1.631] |
|  | LEO | 1.65±0.15 | 1.40±0.38 | <0.01 | 0.02* | 0.87 | [0.309, 1.427] |
|  | LEC | 1.46±0.11 | 1.31±0.25 | 0.01 | 0.07 | 0.75 | [0.199, 1.305] |
| ML_MSE_Scale5 | DEO | 2.02±0.13 | 1.70±0.47 | <0.01 | 0.01* | 0.97 | [0.401, 1.531] |
|  | DEC | 2.07±0.09 | 1.61±0.52 | <0.001 | <0.001*** | 1.27 | [0.684, 1.858] |
|  | REO | 1.79±0.12 | 1.56±0.35 | <0.01 | 0.02* | 0.87 | [0.312, 1.430] |
|  | REC | 1.61±0.14 | 1.40±0.26 | <0.01 | 0.01* | 0.99 | [0.420, 1.553] |
|  | LEO | 1.72±0.13 | 1.49±0.38 | <0.01 | 0.04* | 0.82 | [0.262, 1.375] |
|  | LEC | 1.59±0.13 | 1.46±0.26 | 0.03 | 0.18 | 0.63 | [0.085, 1.180] |
| ML_MSE_Scale6 | DEO | 1.91±0.14 | 1.65±0.42 | <0.01 | 0.03* | 0.84 | [0.278, 1.392] |
|  | DEC | 1.98±0.07 | 1.57±0.47 | <0.001 | <0.01** | 1.24 | [0.654, 1.823] |
|  | REO | 1.80±0.14 | 1.62±0.30 | <0.01 | 0.05 | 0.78 | [0.222, 1.331] |
|  | REC | 1.69±0.15 | 1.52±0.25 | <0.01 | 0.02* | 0.87 | [0.312, 1.431] |
|  | LEO | 1.78±0.15 | 1.55±0.34 | <0.01 | 0.02* | 0.89 | [0.325, 1.445] |
|  | LEC | 1.68±0.12 | 1.57±0.24 | 0.046 | 0.27 | 0.58 | [0.030, 1.121] |
| ML_MSE_Scale7 | DEO | 1.81±0.16 | 1.58±0.36 | <0.01 | 0.04* | 0.81 | [0.250, 1.361] |
|  | DEC | 1.89±0.08 | 1.51±0.40 | <0.001 | <0.001*** | 1.31 | [0.722, 1.903] |
|  | REO | 1.83±0.13 | 1.68±0.25 | 0.01 | 0.08 | 0.72 | [0.169, 1.272] |
|  | REC | 1.74±0.15 | 1.62±0.25 | 0.04 | 0.25 | 0.58 | [0.035, 1.126] |
|  | LEO | 1.80±0.15 | 1.62±0.33 | 0.01 | 0.08 | 0.73 | [0.173, 1.277] |
|  | LEC | 1.73±0.14 | 1.66±0.23 | 0.16 | 0.93 | 0.4 | [-0.138, 0.940] |
| ML_MSE_Scale8 | DEO | 1.71±0.14 | 1.57±0.35 | 0.07 | 0.43 | 0.52 | [-0.022, 1.065] |
|  | DEC | 1.77±0.10 | 1.48±0.38 | <0.01 | <0.01** | 1.08 | [0.508, 1.654] |
|  | REO | 1.80±0.12 | 1.71±0.20 | 0.048 | 0.29 | 0.57 | [0.021, 1.111] |
|  | REC | 1.79±0.15 | 1.69±0.24 | 0.08 | 0.46 | 0.50 | [-0.040, 1.045] |
|  | LEO | 1.81±0.14 | 1.67±0.29 | 0.03 | 0.16 | 0.65 | [0.100, 1.196] |
|  | LEC | 1.82±0.14 | 1.71±0.21 | 0.04 | 0.24 | 0.58 | [0.038, 1.129] |

Abbreviations: Double-leg stance with eyes open (DEO); Double-leg stance with eyes closed (DEC); Right-leg stance with eyes open (REO); Right-leg stance with eyes closed (REC); Left-leg stance with eyes open (LEO); Left-leg stance with eyes closed (LEC);***P < 0.001, **< 0.01, * P < 0.05, Data are presented as Mean± SD. P values are uncorrected for multiple comparisons. Pcorr values represent p-values for simple effects adjusted for multiple comparisons using the Bonferroni correction . Where homogeneity of variance was violated, Welch’s t-test was applied for between-group comparisons. Cohen's d represents the effect size, and CI represents the 95% confidence interval of the mean difference.Statistical significance is determined based on Pcorr.

Appendix 3:Spearman correlation coefficients for COP and MSE Sum parameters.

| **Group** | **Conditions** | **COP** | **MSE** | **Sample(n)** | **Spearman_r** | **p** | **FDR_p** |
| --- | --- | --- | --- | --- | --- | --- | --- |
| GJH | DEO | AP_Vel_SD (mm/s) | AP_MSE_Sum | 28 | -0.590 | 0.001 | 0.024^*^ |
|  |  | LFS (1/mm) | AP_MSE_Sum | 28 | 0.567 | 0.002 | 0.024^*^ |
|  |  | CHA (mm²) | AP_MSE_Sum | 28 | -0.555 | 0.002 | 0.024^*^ |
|  |  | Area_95 (mm²) | AP_MSE_Sum | 28 | -0.543 | 0.003 | 0.024^*^ |
|  |  | AP_Amp (mm) | AP_MSE_Sum | 28 | -0.473 | 0.011 | 0.051 |
|  |  | AP_SD (mm) | AP_MSE_Sum | 28 | -0.471 | 0.011 | 0.051 |
|  |  | AP_RMS (mm) | AP_MSE_Sum | 28 | -0.471 | 0.011 | 0.051 |
|  |  | ML_SD (mm) | AP_MSE_Sum | 28 | -0.461 | 0.013 | 0.051 |
|  |  | ML_RMS (mm) | AP_MSE_Sum | 28 | -0.461 | 0.013 | 0.051 |
|  |  | MV (mm/s) | AP_MSE_Sum | 28 | -0.434 | 0.021 | 0.072 |
|  |  | AP_PL (mm) | AP_MSE_Sum | 28 | -0.421 | 0.026 | 0.079 |
|  |  | ML_Amp (mm) | AP_MSE_Sum | 28 | -0.389 | 0.041 | 0.115 |
|  |  | ML_Vel_SD (mm/s) | AP_MSE_Sum | 28 | -0.382 | 0.045 | 0.118 |
|  |  | AP_SI (%) | AP_MSE_Sum | 28 | -0.365 | 0.056 | 0.127 |
|  |  | ML_SI (%) | AP_MSE_Sum | 28 | 0.365 | 0.056 | 0.127 |
|  |  | TPL (mm) | AP_MSE_Sum | 28 | -0.332 | 0.085 | 0.154 |
|  |  | ML_PL (mm) | AP_MSE_Sum | 28 | -0.097 | 0.624 | 0.703 |
|  |  | CHA (mm²) | ML_MSE_Sum | 28 | -0.344 | 0.073 | 0.154 |
|  |  | AP_SD (mm) | ML_MSE_Sum | 28 | -0.330 | 0.086 | 0.154 |
|  |  | AP_RMS (mm) | ML_MSE_Sum | 28 | -0.330 | 0.086 | 0.154 |
|  |  | Area_95 (mm²) | ML_MSE_Sum | 28 | -0.319 | 0.098 | 0.167 |
|  |  | ML_Vel_SD (mm/s) | ML_MSE_Sum | 28 | -0.293 | 0.131 | 0.211 |
|  |  | AP_Amp (mm) | ML_MSE_Sum | 28 | -0.282 | 0.145 | 0.224 |
|  |  | LFS (1/mm) | ML_MSE_Sum | 28 | 0.244 | 0.210 | 0.311 |
|  |  | AP_Vel_SD (mm/s) | ML_MSE_Sum | 28 | -0.235 | 0.229 | 0.325 |
|  |  | ML_Amp (mm) | ML_MSE_Sum | 28 | -0.189 | 0.334 | 0.455 |
|  |  | ML_SD (mm) | ML_MSE_Sum | 28 | -0.179 | 0.363 | 0.457 |
|  |  | ML_RMS (mm) | ML_MSE_Sum | 28 | -0.179 | 0.363 | 0.457 |
|  |  | ML_PL (mm) | ML_MSE_Sum | 28 | 0.172 | 0.382 | 0.464 |
|  |  | TPL (mm) | ML_MSE_Sum | 28 | 0.156 | 0.428 | 0.502 |
|  |  | MV (mm/s) | ML_MSE_Sum | 28 | -0.092 | 0.641 | 0.703 |
|  |  | AP_PL (mm) | ML_MSE_Sum | 28 | 0.073 | 0.713 | 0.758 |
|  |  | AP_SI (%) | ML_MSE_Sum | 28 | 0.014 | 0.943 | 0.943 |
|  |  | ML_SI (%) | ML_MSE_Sum | 28 | -0.014 | 0.943 | 0.943 |
|  | DEC | AP_Vel_SD (mm/s) | AP_MSE_Sum | 28 | -0.647 | 0.000 | 0.007 |
|  |  | MV (mm/s) | AP_MSE_Sum | 28 | -0.407 | 0.031 | 0.205 |
|  |  | CHA (mm²) | AP_MSE_Sum | 28 | -0.401 | 0.034 | 0.205 |
|  |  | AP_SD (mm) | AP_MSE_Sum | 28 | -0.401 | 0.034 | 0.205 |
|  |  | AP_RMS (mm) | AP_MSE_Sum | 28 | -0.401 | 0.034 | 0.205 |
|  |  | LFS (1/mm) | AP_MSE_Sum | 28 | 0.376 | 0.048 | 0.205 |
|  |  | AP_SI (%) | AP_MSE_Sum | 28 | -0.359 | 0.060 | 0.205 |
|  |  | ML_SI (%) | AP_MSE_Sum | 28 | 0.359 | 0.060 | 0.205 |
|  |  | Area_95 (mm²) | AP_MSE_Sum | 28 | -0.351 | 0.067 | 0.207 |
|  |  | AP_PL (mm) | AP_MSE_Sum | 28 | -0.338 | 0.079 | 0.223 |
|  |  | AP_Amp (mm) | AP_MSE_Sum | 28 | -0.331 | 0.085 | 0.223 |
|  |  | ML_Amp (mm) | AP_MSE_Sum | 28 | -0.304 | 0.116 | 0.254 |
|  |  | ML_SD (mm) | AP_MSE_Sum | 28 | -0.296 | 0.127 | 0.254 |
|  |  | ML_RMS (mm) | AP_MSE_Sum | 28 | -0.296 | 0.127 | 0.254 |
|  |  | ML_Vel_SD (mm/s) | AP_MSE_Sum | 28 | -0.266 | 0.172 | 0.324 |
|  |  | TPL (mm) | AP_MSE_Sum | 28 | -0.254 | 0.191 | 0.342 |
|  |  | ML_PL (mm) | AP_MSE_Sum | 28 | 0.008 | 0.969 | 0.969 |
|  |  | AP_SI (%) | ML_MSE_Sum | 28 | 0.366 | 0.056 | 0.205 |
|  |  | ML_SI (%) | ML_MSE_Sum | 28 | -0.366 | 0.056 | 0.205 |
|  |  | ML_Vel_SD (mm/s) | ML_MSE_Sum | 28 | -0.304 | 0.116 | 0.254 |
|  |  | ML_Amp (mm) | ML_MSE_Sum | 28 | -0.196 | 0.318 | 0.541 |
|  |  | LFS (1/mm) | ML_MSE_Sum | 28 | 0.185 | 0.347 | 0.562 |
|  |  | ML_PL (mm) | ML_MSE_Sum | 28 | -0.174 | 0.376 | 0.567 |
|  |  | CHA (mm²) | ML_MSE_Sum | 28 | -0.170 | 0.387 | 0.567 |
|  |  | AP_PL (mm) | ML_MSE_Sum | 28 | 0.165 | 0.400 | 0.567 |
|  |  | AP_SD (mm) | ML_MSE_Sum | 28 | -0.146 | 0.458 | 0.587 |
|  |  | AP_RMS (mm) | ML_MSE_Sum | 28 | -0.146 | 0.458 | 0.587 |
|  |  | Area_95 (mm²) | ML_MSE_Sum | 28 | -0.143 | 0.466 | 0.587 |
|  |  | AP_Vel_SD (mm/s) | ML_MSE_Sum | 28 | 0.090 | 0.650 | 0.784 |
|  |  | TPL (mm) | ML_MSE_Sum | 28 | 0.084 | 0.670 | 0.784 |
|  |  | ML_SD (mm) | ML_MSE_Sum | 28 | -0.069 | 0.728 | 0.784 |
|  |  | ML_RMS (mm) | ML_MSE_Sum | 28 | -0.069 | 0.728 | 0.784 |
|  |  | AP_Amp (mm) | ML_MSE_Sum | 28 | -0.066 | 0.738 | 0.784 |
|  |  | MV (mm/s) | ML_MSE_Sum | 28 | 0.034 | 0.864 | 0.890 |
|  | REO | AP_Vel_SD (mm/s) | AP_MSE_Sum | 28 | -0.493 | 0.008 | 0.113 |
|  |  | MV (mm/s) | AP_MSE_Sum | 28 | -0.473 | 0.011 | 0.113 |
|  |  | ML_Vel_SD (mm/s) | AP_MSE_Sum | 28 | -0.462 | 0.013 | 0.113 |
|  |  | ML_PL (mm) | AP_MSE_Sum | 28 | -0.436 | 0.020 | 0.127 |
|  |  | TPL (mm) | AP_MSE_Sum | 28 | -0.426 | 0.024 | 0.127 |
|  |  | AP_PL (mm) | AP_MSE_Sum | 28 | -0.420 | 0.026 | 0.127 |
|  |  | ML_Amp (mm) | AP_MSE_Sum | 28 | -0.362 | 0.058 | 0.192 |
|  |  | CHA (mm²) | AP_MSE_Sum | 28 | -0.240 | 0.219 | 0.510 |
|  |  | ML_SD (mm) | AP_MSE_Sum | 28 | -0.204 | 0.299 | 0.564 |
|  |  | ML_RMS (mm) | AP_MSE_Sum | 28 | -0.204 | 0.299 | 0.564 |
|  |  | AP_SI (%) | AP_MSE_Sum | 28 | -0.117 | 0.554 | 0.763 |
|  |  | ML_SI (%) | AP_MSE_Sum | 28 | 0.117 | 0.554 | 0.763 |
|  |  | AP_Amp (mm) | AP_MSE_Sum | 28 | -0.116 | 0.558 | 0.763 |
|  |  | Area_95 (mm²) | AP_MSE_Sum | 28 | -0.115 | 0.561 | 0.763 |
|  |  | LFS (1/mm) | AP_MSE_Sum | 28 | -0.026 | 0.894 | 0.991 |
|  |  | AP_SD (mm) | AP_MSE_Sum | 28 | -0.014 | 0.944 | 0.991 |
|  |  | AP_RMS (mm) | AP_MSE_Sum | 28 | -0.014 | 0.944 | 0.991 |
|  |  | ML_Vel_SD (mm/s) | ML_MSE_Sum | 28 | -0.505 | 0.006 | 0.113 |
|  |  | ML_PL (mm) | ML_MSE_Sum | 28 | -0.365 | 0.056 | 0.192 |
|  |  | MV (mm/s) | ML_MSE_Sum | 28 | -0.364 | 0.057 | 0.192 |
|  |  | TPL (mm) | ML_MSE_Sum | 28 | -0.357 | 0.062 | 0.192 |
|  |  | AP_PL (mm) | ML_MSE_Sum | 28 | -0.349 | 0.068 | 0.194 |
|  |  | AP_Vel_SD (mm/s) | ML_MSE_Sum | 28 | -0.311 | 0.107 | 0.279 |
|  |  | ML_Amp (mm) | ML_MSE_Sum | 28 | -0.237 | 0.225 | 0.510 |
|  |  | CHA (mm²) | ML_MSE_Sum | 28 | -0.213 | 0.276 | 0.564 |
|  |  | ML_SD (mm) | ML_MSE_Sum | 28 | -0.146 | 0.457 | 0.763 |
|  |  | ML_RMS (mm) | ML_MSE_Sum | 28 | -0.146 | 0.457 | 0.763 |
|  |  | LFS (1/mm) | ML_MSE_Sum | 28 | -0.136 | 0.489 | 0.763 |
|  |  | Area_95 (mm²) | ML_MSE_Sum | 28 | -0.074 | 0.706 | 0.924 |
|  |  | AP_SI (%) | ML_MSE_Sum | 28 | 0.046 | 0.816 | 0.991 |
|  |  | ML_SI (%) | ML_MSE_Sum | 28 | -0.046 | 0.816 | 0.991 |
|  |  | AP_Amp (mm) | ML_MSE_Sum | 28 | -0.019 | 0.922 | 0.991 |
|  |  | AP_SD (mm) | ML_MSE_Sum | 28 | -0.002 | 0.991 | 0.991 |
|  |  | AP_RMS (mm) | ML_MSE_Sum | 28 | -0.002 | 0.991 | 0.991 |
|  | REC | ML_Vel_SD (mm/s) | AP_MSE_Sum | 28 | -0.407 | 0.032 | 0.108 |
|  |  | ML_Amp (mm) | AP_MSE_Sum | 28 | -0.396 | 0.037 | 0.108 |
|  |  | CHA (mm²) | AP_MSE_Sum | 28 | -0.394 | 0.038 | 0.108 |
|  |  | AP_Vel_SD (mm/s) | AP_MSE_Sum | 28 | -0.388 | 0.041 | 0.108 |
|  |  | MV (mm/s) | AP_MSE_Sum | 28 | -0.364 | 0.057 | 0.108 |
|  |  | TPL (mm) | AP_MSE_Sum | 28 | -0.358 | 0.061 | 0.108 |
|  |  | AP_Amp (mm) | AP_MSE_Sum | 28 | -0.358 | 0.062 | 0.108 |
|  |  | LFS (1/mm) | AP_MSE_Sum | 28 | 0.355 | 0.064 | 0.108 |
|  |  | AP_SD (mm) | AP_MSE_Sum | 28 | -0.351 | 0.067 | 0.108 |
|  |  | AP_RMS (mm) | AP_MSE_Sum | 28 | -0.351 | 0.067 | 0.108 |
|  |  | ML_PL (mm) | AP_MSE_Sum | 28 | -0.347 | 0.071 | 0.108 |
|  |  | ML_SD (mm) | AP_MSE_Sum | 28 | -0.346 | 0.071 | 0.108 |
|  |  | ML_RMS (mm) | AP_MSE_Sum | 28 | -0.346 | 0.071 | 0.108 |
|  |  | Area_95 (mm²) | AP_MSE_Sum | 28 | -0.345 | 0.072 | 0.108 |
|  |  | AP_PL (mm) | AP_MSE_Sum | 28 | -0.335 | 0.081 | 0.115 |
|  |  | AP_SI (%) | AP_MSE_Sum | 28 | -0.150 | 0.447 | 0.447 |
|  |  | ML_SI (%) | AP_MSE_Sum | 28 | 0.150 | 0.447 | 0.447 |
|  |  | ML_Vel_SD (mm/s) | ML_MSE_Sum | 28 | -0.627 | 0.000 | 0.012^*^ |
|  |  | ML_SD (mm) | ML_MSE_Sum | 28 | -0.491 | 0.008 | 0.090 |
|  |  | ML_RMS (mm) | ML_MSE_Sum | 28 | -0.491 | 0.008 | 0.090 |
|  |  | ML_Amp (mm) | ML_MSE_Sum | 28 | -0.437 | 0.020 | 0.108 |
|  |  | ML_PL (mm) | ML_MSE_Sum | 28 | -0.433 | 0.021 | 0.108 |
|  |  | MV (mm/s) | ML_MSE_Sum | 28 | -0.431 | 0.022 | 0.108 |
|  |  | CHA (mm²) | ML_MSE_Sum | 28 | -0.414 | 0.029 | 0.108 |
|  |  | Area_95 (mm²) | ML_MSE_Sum | 28 | -0.365 | 0.056 | 0.108 |
|  |  | LFS (1/mm) | ML_MSE_Sum | 28 | 0.344 | 0.073 | 0.108 |
|  |  | AP_Vel_SD (mm/s) | ML_MSE_Sum | 28 | -0.325 | 0.091 | 0.124 |
|  |  | TPL (mm) | ML_MSE_Sum | 28 | -0.322 | 0.095 | 0.124 |
|  |  | AP_SI (%) | ML_MSE_Sum | 28 | 0.245 | 0.209 | 0.254 |
|  |  | ML_SI (%) | ML_MSE_Sum | 28 | -0.245 | 0.209 | 0.254 |
|  |  | AP_PL (mm) | ML_MSE_Sum | 28 | -0.216 | 0.270 | 0.297 |
|  |  | AP_SD (mm) | ML_MSE_Sum | 28 | -0.216 | 0.271 | 0.297 |
|  |  | AP_RMS (mm) | ML_MSE_Sum | 28 | -0.216 | 0.271 | 0.297 |
|  |  | AP_Amp (mm) | ML_MSE_Sum | 28 | -0.194 | 0.323 | 0.344 |
|  | LEO | AP_SI (%) | AP_MSE_Sum | 28 | -0.569 | 0.002 | 0.016^*^ |
|  |  | ML_SI (%) | AP_MSE_Sum | 28 | 0.569 | 0.002 | 0.016^*^ |
|  |  | AP_SD (mm) | AP_MSE_Sum | 28 | -0.501 | 0.007 | 0.019^*^ |
|  |  | AP_RMS (mm) | AP_MSE_Sum | 28 | -0.501 | 0.007 | 0.019^*^ |
|  |  | AP_Vel_SD (mm/s) | AP_MSE_Sum | 28 | -0.418 | 0.027 | 0.065 |
|  |  | Area_95 (mm²) | AP_MSE_Sum | 28 | -0.375 | 0.049 | 0.094 |
|  |  | AP_Amp (mm) | AP_MSE_Sum | 28 | -0.375 | 0.050 | 0.094 |
|  |  | CHA (mm²) | AP_MSE_Sum | 28 | -0.370 | 0.053 | 0.095 |
|  |  | MV (mm/s) | AP_MSE_Sum | 28 | -0.263 | 0.177 | 0.262 |
|  |  | LFS (1/mm) | AP_MSE_Sum | 28 | 0.249 | 0.202 | 0.286 |
|  |  | ML_Vel_SD (mm/s) | AP_MSE_Sum | 28 | -0.202 | 0.304 | 0.383 |
|  |  | AP_PL (mm) | AP_MSE_Sum | 28 | -0.185 | 0.347 | 0.422 |
|  |  | TPL (mm) | AP_MSE_Sum | 28 | -0.151 | 0.443 | 0.520 |
|  |  | ML_Amp (mm) | AP_MSE_Sum | 28 | -0.146 | 0.460 | 0.521 |
|  |  | ML_SD (mm) | AP_MSE_Sum | 28 | -0.103 | 0.600 | 0.618 |
|  |  | ML_RMS (mm) | AP_MSE_Sum | 28 | -0.103 | 0.600 | 0.618 |
|  |  | ML_PL (mm) | AP_MSE_Sum | 28 | -0.085 | 0.667 | 0.667 |
|  |  | ML_Vel_SD (mm/s) | ML_MSE_Sum | 28 | -0.595 | 0.001 | 0.016^*^ |
|  |  | CHA (mm²) | ML_MSE_Sum | 28 | -0.554 | 0.002 | 0.016^*^ |
|  |  | AP_Vel_SD (mm/s) | ML_MSE_Sum | 28 | -0.550 | 0.002 | 0.016^*^ |
|  |  | MV (mm/s) | ML_MSE_Sum | 28 | -0.537 | 0.003 | 0.016^*^ |
|  |  | AP_Amp (mm) | ML_MSE_Sum | 28 | -0.530 | 0.004 | 0.016^*^ |
|  |  | Area_95 (mm²) | ML_MSE_Sum | 28 | -0.527 | 0.004 | 0.016^*^ |
|  |  | TPL (mm) | ML_MSE_Sum | 28 | -0.524 | 0.004 | 0.016^*^ |
|  |  | AP_PL (mm) | ML_MSE_Sum | 28 | -0.502 | 0.006 | 0.019^*^ |
|  |  | ML_PL (mm) | ML_MSE_Sum | 28 | -0.493 | 0.008 | 0.020^*^ |
|  |  | AP_SD (mm) | ML_MSE_Sum | 28 | -0.375 | 0.049 | 0.094 |
|  |  | AP_RMS (mm) | ML_MSE_Sum | 28 | -0.375 | 0.049 | 0.094 |
|  |  | ML_SD (mm) | ML_MSE_Sum | 28 | -0.318 | 0.099 | 0.160 |
|  |  | ML_RMS (mm) | ML_MSE_Sum | 28 | -0.318 | 0.099 | 0.160 |
|  |  | ML_Amp (mm) | ML_MSE_Sum | 28 | -0.307 | 0.112 | 0.173 |
|  |  | AP_SI (%) | ML_MSE_Sum | 28 | -0.205 | 0.296 | 0.383 |
|  |  | ML_SI (%) | ML_MSE_Sum | 28 | 0.205 | 0.296 | 0.383 |
|  |  | LFS (1/mm) | ML_MSE_Sum | 28 | 0.104 | 0.599 | 0.618 |
|  | LEC | AP_Vel_SD (mm/s) | AP_MSE_Sum | 28 | -0.567 | 0.002 | 0.034^*^ |
|  |  | CHA (mm²) | AP_MSE_Sum | 28 | -0.559 | 0.002 | 0.034^*^ |
|  |  | AP_Amp (mm) | AP_MSE_Sum | 28 | -0.519 | 0.005 | 0.039^*^ |
|  |  | ML_Amp (mm) | AP_MSE_Sum | 28 | -0.507 | 0.006 | 0.040^*^ |
|  |  | Area_95 (mm²) | AP_MSE_Sum | 28 | -0.470 | 0.012 | 0.047^*^ |
|  |  | AP_SD (mm) | AP_MSE_Sum | 28 | -0.459 | 0.014 | 0.047^*^ |
|  |  | AP_RMS (mm) | AP_MSE_Sum | 28 | -0.459 | 0.014 | 0.047^*^ |
|  |  | LFS (1/mm) | AP_MSE_Sum | 28 | 0.450 | 0.016 | 0.050 |
|  |  | ML_SD (mm) | AP_MSE_Sum | 28 | -0.436 | 0.020 | 0.053 |
|  |  | ML_RMS (mm) | AP_MSE_Sum | 28 | -0.436 | 0.020 | 0.053 |
|  |  | AP_SI (%) | AP_MSE_Sum | 28 | -0.416 | 0.028 | 0.063 |
|  |  | ML_SI (%) | AP_MSE_Sum | 28 | 0.416 | 0.028 | 0.063 |
|  |  | AP_PL (mm) | AP_MSE_Sum | 28 | -0.331 | 0.085 | 0.138 |
|  |  | TPL (mm) | AP_MSE_Sum | 28 | -0.317 | 0.100 | 0.152 |
|  |  | MV (mm/s) | AP_MSE_Sum | 28 | -0.279 | 0.150 | 0.192 |
|  |  | ML_Vel_SD (mm/s) | AP_MSE_Sum | 28 | -0.191 | 0.331 | 0.352 |
|  |  | ML_PL (mm) | AP_MSE_Sum | 28 | -0.149 | 0.449 | 0.463 |
|  |  | LFS (1/mm) | ML_MSE_Sum | 28 | 0.529 | 0.004 | 0.039^*^ |
|  |  | AP_SD (mm) | ML_MSE_Sum | 28 | -0.465 | 0.013 | 0.047^*^ |
|  |  | AP_RMS (mm) | ML_MSE_Sum | 28 | -0.465 | 0.013 | 0.047^*^ |
|  |  | Area_95 (mm²) | ML_MSE_Sum | 28 | -0.408 | 0.031 | 0.066 |
|  |  | ML_Amp (mm) | ML_MSE_Sum | 28 | -0.362 | 0.059 | 0.114 |
|  |  | CHA (mm²) | ML_MSE_Sum | 28 | -0.359 | 0.060 | 0.114 |
|  |  | ML_SD (mm) | ML_MSE_Sum | 28 | -0.343 | 0.074 | 0.126 |
|  |  | ML_RMS (mm) | ML_MSE_Sum | 28 | -0.343 | 0.074 | 0.126 |
|  |  | AP_Amp (mm) | ML_MSE_Sum | 28 | -0.314 | 0.104 | 0.152 |
|  |  | AP_SI (%) | ML_MSE_Sum | 28 | -0.307 | 0.112 | 0.152 |
|  |  | ML_SI (%) | ML_MSE_Sum | 28 | 0.307 | 0.112 | 0.152 |
|  |  | MV (mm/s) | ML_MSE_Sum | 28 | -0.278 | 0.152 | 0.192 |
|  |  | TPL (mm) | ML_MSE_Sum | 28 | -0.243 | 0.213 | 0.259 |
|  |  | AP_Vel_SD (mm/s) | ML_MSE_Sum | 28 | -0.238 | 0.222 | 0.261 |
|  |  | ML_Vel_SD (mm/s) | ML_MSE_Sum | 28 | -0.217 | 0.267 | 0.302 |
|  |  | AP_PL (mm) | ML_MSE_Sum | 28 | -0.200 | 0.307 | 0.336 |
|  |  | ML_PL (mm) | ML_MSE_Sum | 28 | -0.143 | 0.469 | 0.469 |
| Healthy | DEO | Area_95 (mm²) | AP_MSE_Sum | 26 | 0.414 | 0.036 | 0.285 |
|  |  | ML_SD (mm) | AP_MSE_Sum | 26 | 0.388 | 0.050 | 0.285 |
|  |  | ML_RMS (mm) | AP_MSE_Sum | 26 | 0.388 | 0.050 | 0.285 |
|  |  | AP_PL (mm) | AP_MSE_Sum | 26 | 0.344 | 0.085 | 0.294 |
|  |  | LFS (1/mm) | AP_MSE_Sum | 26 | -0.318 | 0.114 | 0.322 |
|  |  | ML_Amp (mm) | AP_MSE_Sum | 26 | 0.283 | 0.161 | 0.410 |
|  |  | TPL (mm) | AP_MSE_Sum | 26 | 0.276 | 0.173 | 0.410 |
|  |  | AP_Vel_SD (mm/s) | AP_MSE_Sum | 26 | 0.267 | 0.188 | 0.410 |
|  |  | MV (mm/s) | AP_MSE_Sum | 26 | 0.248 | 0.222 | 0.419 |
|  |  | ML_PL (mm) | AP_MSE_Sum | 26 | 0.180 | 0.379 | 0.644 |
|  |  | CHA (mm²) | AP_MSE_Sum | 26 | 0.166 | 0.417 | 0.644 |
|  |  | ML_Vel_SD (mm/s) | AP_MSE_Sum | 26 | 0.145 | 0.481 | 0.681 |
|  |  | AP_Amp (mm) | AP_MSE_Sum | 26 | 0.112 | 0.585 | 0.787 |
|  |  | AP_SI (%) | AP_MSE_Sum | 26 | 0.044 | 0.829 | 0.899 |
|  |  | AP_RMS (mm) | AP_MSE_Sum | 26 | -0.042 | 0.837 | 0.899 |
|  |  | AP_SD (mm) | AP_MSE_Sum | 26 | -0.033 | 0.872 | 0.899 |
|  |  | ML_SI (%) | AP_MSE_Sum | 26 | -0.017 | 0.935 | 0.935 |
|  |  | AP_PL (mm) | ML_MSE_Sum | 26 | 0.508 | 0.008 | 0.273 |
|  |  | ML_SD (mm) | ML_MSE_Sum | 26 | 0.403 | 0.041 | 0.285 |
|  |  | ML_RMS (mm) | ML_MSE_Sum | 26 | 0.403 | 0.041 | 0.285 |
|  |  | AP_Vel_SD (mm/s) | ML_MSE_Sum | 26 | 0.362 | 0.069 | 0.294 |
|  |  | TPL (mm) | ML_MSE_Sum | 26 | 0.352 | 0.078 | 0.294 |
|  |  | MV (mm/s) | ML_MSE_Sum | 26 | 0.341 | 0.088 | 0.294 |
|  |  | Area_95 (mm²) | ML_MSE_Sum | 26 | 0.334 | 0.095 | 0.294 |
|  |  | ML_PL (mm) | ML_MSE_Sum | 26 | 0.260 | 0.200 | 0.410 |
|  |  | ML_Amp (mm) | ML_MSE_Sum | 26 | 0.257 | 0.205 | 0.410 |
|  |  | LFS (1/mm) | ML_MSE_Sum | 26 | -0.210 | 0.304 | 0.544 |
|  |  | CHA (mm²) | ML_MSE_Sum | 26 | 0.171 | 0.404 | 0.644 |
|  |  | ML_Vel_SD (mm/s) | ML_MSE_Sum | 26 | 0.156 | 0.447 | 0.661 |
|  |  | AP_SD (mm) | ML_MSE_Sum | 26 | 0.107 | 0.602 | 0.787 |
|  |  | AP_RMS (mm) | ML_MSE_Sum | 26 | 0.101 | 0.625 | 0.787 |
|  |  | AP_SI (%) | ML_MSE_Sum | 26 | 0.070 | 0.732 | 0.882 |
|  |  | ML_SI (%) | ML_MSE_Sum | 26 | -0.065 | 0.753 | 0.882 |
|  |  | AP_Amp (mm) | ML_MSE_Sum | 26 | 0.036 | 0.860 | 0.899 |
|  | DEC | AP_PL (mm) | AP_MSE_Sum | 26 | -0.376 | 0.058 | 0.484 |
|  |  | TPL (mm) | AP_MSE_Sum | 26 | -0.368 | 0.064 | 0.484 |
|  |  | MV (mm/s) | AP_MSE_Sum | 26 | -0.368 | 0.064 | 0.484 |
|  |  | AP_Vel_SD (mm/s) | AP_MSE_Sum | 26 | -0.334 | 0.095 | 0.484 |
|  |  | ML_PL (mm) | AP_MSE_Sum | 26 | -0.291 | 0.149 | 0.564 |
|  |  | ML_Vel_SD (mm/s) | AP_MSE_Sum | 26 | -0.246 | 0.225 | 0.639 |
|  |  | CHA (mm²) | AP_MSE_Sum | 26 | -0.154 | 0.453 | 0.891 |
|  |  | ML_Amp (mm) | AP_MSE_Sum | 26 | -0.151 | 0.460 | 0.891 |
|  |  | Area_95 (mm²) | AP_MSE_Sum | 26 | -0.144 | 0.483 | 0.891 |
|  |  | ML_SD (mm) | AP_MSE_Sum | 26 | -0.135 | 0.511 | 0.891 |
|  |  | ML_RMS (mm) | AP_MSE_Sum | 26 | -0.135 | 0.511 | 0.891 |
|  |  | AP_SD (mm) | AP_MSE_Sum | 26 | -0.067 | 0.746 | 0.903 |
|  |  | AP_RMS (mm) | AP_MSE_Sum | 26 | -0.067 | 0.746 | 0.903 |
|  |  | AP_Amp (mm) | AP_MSE_Sum | 26 | -0.057 | 0.782 | 0.903 |
|  |  | LFS (1/mm) | AP_MSE_Sum | 26 | -0.023 | 0.912 | 0.941 |
|  |  | AP_SI (%) | AP_MSE_Sum | 26 | 0.015 | 0.941 | 0.941 |
|  |  | ML_SI (%) | AP_MSE_Sum | 26 | -0.015 | 0.941 | 0.941 |
|  |  | AP_Vel_SD (mm/s) | ML_MSE_Sum | 26 | -0.352 | 0.078 | 0.484 |
|  |  | TPL (mm) | ML_MSE_Sum | 26 | -0.330 | 0.100 | 0.484 |
|  |  | MV (mm/s) | ML_MSE_Sum | 26 | -0.330 | 0.100 | 0.484 |
|  |  | AP_PL (mm) | ML_MSE_Sum | 26 | -0.311 | 0.122 | 0.520 |
|  |  | ML_Vel_SD (mm/s) | ML_MSE_Sum | 26 | -0.256 | 0.207 | 0.639 |
|  |  | ML_PL (mm) | ML_MSE_Sum | 26 | -0.253 | 0.213 | 0.639 |
|  |  | ML_SD (mm) | ML_MSE_Sum | 26 | -0.147 | 0.472 | 0.891 |
|  |  | ML_RMS (mm) | ML_MSE_Sum | 26 | -0.147 | 0.472 | 0.891 |
|  |  | CHA (mm²) | ML_MSE_Sum | 26 | -0.130 | 0.527 | 0.891 |
|  |  | Area_95 (mm²) | ML_MSE_Sum | 26 | -0.123 | 0.550 | 0.891 |
|  |  | ML_Amp (mm) | ML_MSE_Sum | 26 | -0.105 | 0.610 | 0.903 |
|  |  | AP_Amp (mm) | ML_MSE_Sum | 26 | -0.074 | 0.721 | 0.903 |
|  |  | AP_SD (mm) | ML_MSE_Sum | 26 | -0.063 | 0.761 | 0.903 |
|  |  | AP_RMS (mm) | ML_MSE_Sum | 26 | -0.063 | 0.761 | 0.903 |
|  |  | AP_SI (%) | ML_MSE_Sum | 26 | 0.053 | 0.797 | 0.903 |
|  |  | ML_SI (%) | ML_MSE_Sum | 26 | -0.053 | 0.797 | 0.903 |
|  |  | LFS (1/mm) | ML_MSE_Sum | 26 | -0.027 | 0.896 | 0.941 |
|  | REO | LFS (1/mm) | AP_MSE_Sum | 26 | 0.273 | 0.177 | 0.767 |
|  |  | AP_SI (%) | AP_MSE_Sum | 26 | 0.235 | 0.248 | 0.767 |
|  |  | ML_SI (%) | AP_MSE_Sum | 26 | -0.235 | 0.248 | 0.767 |
|  |  | Area_95 (mm²) | AP_MSE_Sum | 26 | -0.157 | 0.444 | 0.929 |
|  |  | ML_SD (mm) | AP_MSE_Sum | 26 | -0.144 | 0.484 | 0.929 |
|  |  | ML_RMS (mm) | AP_MSE_Sum | 26 | -0.144 | 0.484 | 0.929 |
|  |  | AP_PL (mm) | AP_MSE_Sum | 26 | 0.140 | 0.496 | 0.929 |
|  |  | AP_SD (mm) | AP_MSE_Sum | 26 | -0.136 | 0.506 | 0.929 |
|  |  | AP_RMS (mm) | AP_MSE_Sum | 26 | -0.136 | 0.506 | 0.929 |
|  |  | AP_Vel_SD (mm/s) | AP_MSE_Sum | 26 | 0.108 | 0.601 | 0.929 |
|  |  | ML_Amp (mm) | AP_MSE_Sum | 26 | -0.087 | 0.672 | 0.929 |
|  |  | ML_PL (mm) | AP_MSE_Sum | 26 | -0.082 | 0.692 | 0.929 |
|  |  | ML_Vel_SD (mm/s) | AP_MSE_Sum | 26 | -0.065 | 0.751 | 0.929 |
|  |  | CHA (mm²) | AP_MSE_Sum | 26 | -0.022 | 0.915 | 0.988 |
|  |  | AP_Amp (mm) | AP_MSE_Sum | 26 | -0.018 | 0.930 | 0.988 |
|  |  | TPL (mm) | AP_MSE_Sum | 26 | -0.003 | 0.988 | 0.988 |
|  |  | MV (mm/s) | AP_MSE_Sum | 26 | -0.003 | 0.988 | 0.988 |
|  |  | AP_SI (%) | ML_MSE_Sum | 26 | 0.442 | 0.024 | 0.404 |
|  |  | ML_SI (%) | ML_MSE_Sum | 26 | -0.442 | 0.024 | 0.404 |
|  |  | LFS (1/mm) | ML_MSE_Sum | 26 | 0.400 | 0.043 | 0.483 |
|  |  | ML_SD (mm) | ML_MSE_Sum | 26 | -0.341 | 0.088 | 0.601 |
|  |  | ML_RMS (mm) | ML_MSE_Sum | 26 | -0.341 | 0.088 | 0.601 |
|  |  | ML_Amp (mm) | ML_MSE_Sum | 26 | -0.302 | 0.134 | 0.759 |
|  |  | AP_Vel_SD (mm/s) | ML_MSE_Sum | 26 | 0.250 | 0.218 | 0.767 |
|  |  | AP_PL (mm) | ML_MSE_Sum | 26 | 0.242 | 0.233 | 0.767 |
|  |  | Area_95 (mm²) | ML_MSE_Sum | 26 | -0.199 | 0.329 | 0.929 |
|  |  | ML_PL (mm) | ML_MSE_Sum | 26 | -0.099 | 0.631 | 0.929 |
|  |  | ML_Vel_SD (mm/s) | ML_MSE_Sum | 26 | -0.099 | 0.631 | 0.929 |
|  |  | AP_SD (mm) | ML_MSE_Sum | 26 | -0.076 | 0.711 | 0.929 |
|  |  | AP_RMS (mm) | ML_MSE_Sum | 26 | -0.076 | 0.711 | 0.929 |
|  |  | AP_Amp (mm) | ML_MSE_Sum | 26 | 0.070 | 0.734 | 0.929 |
|  |  | CHA (mm²) | ML_MSE_Sum | 26 | -0.062 | 0.765 | 0.929 |
|  |  | TPL (mm) | ML_MSE_Sum | 26 | 0.035 | 0.867 | 0.983 |
|  |  | MV (mm/s) | ML_MSE_Sum | 26 | 0.035 | 0.867 | 0.983 |
|  | REC | AP_SD (mm) | AP_MSE_Sum | 26 | -0.272 | 0.179 | 0.903 |
|  |  | AP_RMS (mm) | AP_MSE_Sum | 26 | -0.272 | 0.179 | 0.903 |
|  |  | TPL (mm) | AP_MSE_Sum | 26 | -0.182 | 0.373 | 0.903 |
|  |  | MV (mm/s) | AP_MSE_Sum | 26 | -0.182 | 0.373 | 0.903 |
|  |  | ML_PL (mm) | AP_MSE_Sum | 26 | -0.164 | 0.423 | 0.903 |
|  |  | Area_95 (mm²) | AP_MSE_Sum | 26 | -0.158 | 0.440 | 0.903 |
|  |  | ML_Vel_SD (mm/s) | AP_MSE_Sum | 26 | -0.149 | 0.468 | 0.903 |
|  |  | ML_Amp (mm) | AP_MSE_Sum | 26 | 0.115 | 0.577 | 0.903 |
|  |  | AP_PL (mm) | AP_MSE_Sum | 26 | -0.104 | 0.615 | 0.903 |
|  |  | AP_Amp (mm) | AP_MSE_Sum | 26 | -0.090 | 0.662 | 0.903 |
|  |  | AP_SI (%) | AP_MSE_Sum | 26 | 0.084 | 0.684 | 0.903 |
|  |  | ML_SI (%) | AP_MSE_Sum | 26 | -0.084 | 0.684 | 0.903 |
|  |  | CHA (mm²) | AP_MSE_Sum | 26 | -0.083 | 0.687 | 0.903 |
|  |  | LFS (1/mm) | AP_MSE_Sum | 26 | 0.066 | 0.750 | 0.903 |
|  |  | ML_SD (mm) | AP_MSE_Sum | 26 | -0.061 | 0.769 | 0.903 |
|  |  | ML_RMS (mm) | AP_MSE_Sum | 26 | -0.061 | 0.769 | 0.903 |
|  |  | AP_Vel_SD (mm/s) | AP_MSE_Sum | 26 | -0.056 | 0.787 | 0.903 |
|  |  | AP_SD (mm) | ML_MSE_Sum | 26 | -0.381 | 0.055 | 0.903 |
|  |  | AP_RMS (mm) | ML_MSE_Sum | 26 | -0.381 | 0.055 | 0.903 |
|  |  | ML_Vel_SD (mm/s) | ML_MSE_Sum | 26 | -0.226 | 0.266 | 0.903 |
|  |  | ML_PL (mm) | ML_MSE_Sum | 26 | -0.223 | 0.274 | 0.903 |
|  |  | TPL (mm) | ML_MSE_Sum | 26 | -0.194 | 0.343 | 0.903 |
|  |  | MV (mm/s) | ML_MSE_Sum | 26 | -0.194 | 0.343 | 0.903 |
|  |  | AP_PL (mm) | ML_MSE_Sum | 26 | -0.157 | 0.444 | 0.903 |
|  |  | ML_Amp (mm) | ML_MSE_Sum | 26 | 0.151 | 0.461 | 0.903 |
|  |  | Area_95 (mm²) | ML_MSE_Sum | 26 | -0.135 | 0.512 | 0.903 |
|  |  | AP_Amp (mm) | ML_MSE_Sum | 26 | -0.092 | 0.654 | 0.903 |
|  |  | CHA (mm²) | ML_MSE_Sum | 26 | -0.059 | 0.774 | 0.903 |
|  |  | AP_Vel_SD (mm/s) | ML_MSE_Sum | 26 | -0.058 | 0.778 | 0.903 |
|  |  | LFS (1/mm) | ML_MSE_Sum | 26 | 0.041 | 0.841 | 0.903 |
|  |  | ML_SD (mm) | ML_MSE_Sum | 26 | 0.039 | 0.850 | 0.903 |
|  |  | ML_RMS (mm) | ML_MSE_Sum | 26 | 0.039 | 0.850 | 0.903 |
|  |  | AP_SI (%) | ML_MSE_Sum | 26 | 0.004 | 0.984 | 0.984 |
|  |  | ML_SI (%) | ML_MSE_Sum | 26 | -0.004 | 0.984 | 0.984 |
|  | LEO | ML_Amp (mm) | AP_MSE_Sum | 26 | -0.520 | 0.006 | 0.128 |
|  |  | ML_SD (mm) | AP_MSE_Sum | 26 | -0.489 | 0.011 | 0.128 |
|  |  | ML_RMS (mm) | AP_MSE_Sum | 26 | -0.489 | 0.011 | 0.128 |
|  |  | CHA (mm²) | AP_MSE_Sum | 26 | -0.432 | 0.027 | 0.186 |
|  |  | Area_95 (mm²) | AP_MSE_Sum | 26 | -0.411 | 0.037 | 0.211 |
|  |  | LFS (1/mm) | AP_MSE_Sum | 26 | 0.387 | 0.051 | 0.221 |
|  |  | AP_Vel_SD (mm/s) | AP_MSE_Sum | 26 | -0.381 | 0.055 | 0.221 |
|  |  | AP_Amp (mm) | AP_MSE_Sum | 26 | -0.367 | 0.065 | 0.222 |
|  |  | AP_SI (%) | AP_MSE_Sum | 26 | -0.268 | 0.186 | 0.352 |
|  |  | ML_SI (%) | AP_MSE_Sum | 26 | 0.268 | 0.186 | 0.352 |
|  |  | AP_PL (mm) | AP_MSE_Sum | 26 | -0.257 | 0.205 | 0.368 |
|  |  | AP_SD (mm) | AP_MSE_Sum | 26 | -0.201 | 0.325 | 0.442 |
|  |  | AP_RMS (mm) | AP_MSE_Sum | 26 | -0.201 | 0.325 | 0.442 |
|  |  | ML_Vel_SD (mm/s) | AP_MSE_Sum | 26 | -0.190 | 0.351 | 0.457 |
|  |  | TPL (mm) | AP_MSE_Sum | 26 | -0.181 | 0.377 | 0.457 |
|  |  | MV (mm/s) | AP_MSE_Sum | 26 | -0.181 | 0.377 | 0.457 |
|  |  | ML_PL (mm) | AP_MSE_Sum | 26 | -0.125 | 0.541 | 0.614 |
|  |  | ML_Amp (mm) | ML_MSE_Sum | 26 | -0.438 | 0.025 | 0.186 |
|  |  | CHA (mm²) | ML_MSE_Sum | 26 | -0.376 | 0.059 | 0.221 |
|  |  | LFS (1/mm) | ML_MSE_Sum | 26 | 0.329 | 0.101 | 0.293 |
|  |  | ML_SD (mm) | ML_MSE_Sum | 26 | -0.318 | 0.113 | 0.293 |
|  |  | ML_RMS (mm) | ML_MSE_Sum | 26 | -0.318 | 0.113 | 0.293 |
|  |  | AP_Amp (mm) | ML_MSE_Sum | 26 | -0.312 | 0.121 | 0.293 |
|  |  | AP_Vel_SD (mm/s) | ML_MSE_Sum | 26 | -0.296 | 0.141 | 0.307 |
|  |  | Area_95 (mm²) | ML_MSE_Sum | 26 | -0.294 | 0.144 | 0.307 |
|  |  | AP_SI (%) | ML_MSE_Sum | 26 | -0.212 | 0.298 | 0.442 |
|  |  | ML_SI (%) | ML_MSE_Sum | 26 | 0.212 | 0.298 | 0.442 |
|  |  | AP_SD (mm) | ML_MSE_Sum | 26 | -0.204 | 0.318 | 0.442 |
|  |  | AP_RMS (mm) | ML_MSE_Sum | 26 | -0.204 | 0.318 | 0.442 |
|  |  | AP_PL (mm) | ML_MSE_Sum | 26 | -0.159 | 0.438 | 0.513 |
|  |  | ML_Vel_SD (mm/s) | ML_MSE_Sum | 26 | -0.063 | 0.759 | 0.792 |
|  |  | TPL (mm) | ML_MSE_Sum | 26 | -0.061 | 0.769 | 0.792 |
|  |  | MV (mm/s) | ML_MSE_Sum | 26 | -0.061 | 0.769 | 0.792 |
|  |  | ML_PL (mm) | ML_MSE_Sum | 26 | -0.018 | 0.930 | 0.930 |
|  | LEC | AP_Amp (mm) | AP_MSE_Sum | 26 | -0.287 | 0.155 | 0.435 |
|  |  | AP_SD (mm) | AP_MSE_Sum | 26 | -0.286 | 0.156 | 0.435 |
|  |  | AP_RMS (mm) | AP_MSE_Sum | 26 | -0.286 | 0.156 | 0.435 |
|  |  | LFS (1/mm) | AP_MSE_Sum | 26 | 0.271 | 0.180 | 0.435 |
|  |  | AP_SI (%) | AP_MSE_Sum | 26 | -0.213 | 0.296 | 0.478 |
|  |  | ML_SI (%) | AP_MSE_Sum | 26 | 0.213 | 0.296 | 0.478 |
|  |  | ML_Amp (mm) | AP_MSE_Sum | 26 | 0.186 | 0.362 | 0.535 |
|  |  | Area_95 (mm²) | AP_MSE_Sum | 26 | -0.129 | 0.530 | 0.751 |
|  |  | CHA (mm²) | AP_MSE_Sum | 26 | -0.089 | 0.665 | 0.879 |
|  |  | ML_SD (mm) | AP_MSE_Sum | 26 | 0.073 | 0.724 | 0.879 |
|  |  | ML_RMS (mm) | AP_MSE_Sum | 26 | 0.073 | 0.724 | 0.879 |
|  |  | ML_PL (mm) | AP_MSE_Sum | 26 | 0.073 | 0.724 | 0.879 |
|  |  | AP_Vel_SD (mm/s) | AP_MSE_Sum | 26 | -0.054 | 0.792 | 0.929 |
|  |  | AP_PL (mm) | AP_MSE_Sum | 26 | -0.046 | 0.823 | 0.933 |
|  |  | ML_Vel_SD (mm/s) | AP_MSE_Sum | 26 | 0.033 | 0.872 | 0.957 |
|  |  | TPL (mm) | AP_MSE_Sum | 26 | 0.009 | 0.964 | 0.985 |
|  |  | MV (mm/s) | AP_MSE_Sum | 26 | 0.004 | 0.985 | 0.985 |
|  |  | AP_SD (mm) | ML_MSE_Sum | 26 | -0.543 | 0.004 | 0.070 |
|  |  | AP_RMS (mm) | ML_MSE_Sum | 26 | -0.543 | 0.004 | 0.070 |
|  |  | AP_Amp (mm) | ML_MSE_Sum | 26 | -0.450 | 0.021 | 0.238 |
|  |  | Area_95 (mm²) | ML_MSE_Sum | 26 | -0.428 | 0.029 | 0.248 |
|  |  | CHA (mm²) | ML_MSE_Sum | 26 | -0.381 | 0.055 | 0.375 |
|  |  | LFS (1/mm) | ML_MSE_Sum | 26 | 0.331 | 0.099 | 0.435 |
|  |  | AP_PL (mm) | ML_MSE_Sum | 26 | -0.327 | 0.104 | 0.435 |
|  |  | AP_Vel_SD (mm/s) | ML_MSE_Sum | 26 | -0.275 | 0.175 | 0.435 |
|  |  | MV (mm/s) | ML_MSE_Sum | 26 | -0.258 | 0.204 | 0.435 |
|  |  | TPL (mm) | ML_MSE_Sum | 26 | -0.254 | 0.211 | 0.435 |
|  |  | ML_SD (mm) | ML_MSE_Sum | 26 | -0.248 | 0.222 | 0.435 |
|  |  | ML_RMS (mm) | ML_MSE_Sum | 26 | -0.248 | 0.222 | 0.435 |
|  |  | AP_SI (%) | ML_MSE_Sum | 26 | -0.244 | 0.230 | 0.435 |
|  |  | ML_SI (%) | ML_MSE_Sum | 26 | 0.244 | 0.230 | 0.435 |
|  |  | ML_Vel_SD (mm/s) | ML_MSE_Sum | 26 | -0.228 | 0.263 | 0.470 |
|  |  | ML_PL (mm) | ML_MSE_Sum | 26 | -0.208 | 0.309 | 0.478 |
|  |  | ML_Amp (mm) | ML_MSE_Sum | 26 | -0.014 | 0.947 | 0.985 |

Abbreviations: Total Path Length(TPL), Antero-posterior Path Length(AP_PL), Medio-lateral Path Length(ML_PL), Antero-posterior Displacement Amplitude(AP_Amp), Medio-lateral Displacement Amplitude(ML_Amp), Antero-posterior Sway Index(AP_SI), Medio-lateral Sway Index(ML_SI), Antero-posterior Displacement Standard Deviation(AP_SD), Medio-lateral Displacement Standard Deviation(ML_SD), Total Mean Velocity(MV), Antero-posterior Velocity Standard Deviation(AP_Vel_SD), Medio-lateral Velocity Standard Deviation(ML_Vel_SD), Antero-posterior Root-Mean-Square Displacement(AP_RMS), Medio-lateral Root-Mean-Square Displacement(ML_RMS), 95% Confidence Ellipse Area(Area_95), Convex Hull Area(CHA). double-leg stance with eyes open (DEO), double-leg stance with eyes closed (DEC), right-leg stance with eyes open (REO); 4) right-leg stance with eyes closed (REC), left-leg stance with eyes open (LEO), left-leg stance with eyes closed (LEC). *, P<0.05.

Appendix 4 Between-group comparisons of COP-MSE Spearman correlations (Fisher's r-to-z transformation), FDR-corrected across all 204 pairs.

| **Condition** | **COP Indicator** | **MSE Metric** | **GJH_r** | **GJH_n** | **Healthy_r** | **Healthy_n** | **Fisher_Z** | **P** | **FDR_P** |
| --- | --- | --- | --- | --- | --- | --- | --- | --- | --- |
| DEC | AP_Amp (mm) | AP_MSE_Sum | -0.331 | 28 | -0.057 | 26 | -0.993 | 0.321 | 0.661 |
|  | AP_PL (mm) | AP_MSE_Sum | -0.338 | 28 | -0.376 | 26 | 0.151 | 0.880 | 0.962 |
|  | AP_RMS (mm) | AP_MSE_Sum | -0.401 | 28 | -0.067 | 26 | -1.238 | 0.216 | 0.574 |
|  | AP_SD (mm) | AP_MSE_Sum | -0.401 | 28 | -0.067 | 26 | -1.238 | 0.216 | 0.574 |
|  | AP_SI (%) | AP_MSE_Sum | -0.359 | 28 | 0.015 | 26 | -1.352 | 0.176 | 0.545 |
|  | AP_Vel_SD (mm/s) | AP_MSE_Sum | -0.647 | 28 | -0.334 | 26 | -1.463 | 0.143 | 0.496 |
|  | Area_95 (mm²) | AP_MSE_Sum | -0.351 | 28 | -0.144 | 26 | -0.767 | 0.443 | 0.741 |
|  | CHA (mm²) | AP_MSE_Sum | -0.401 | 28 | -0.154 | 26 | -0.933 | 0.351 | 0.688 |
|  | LFS (1/mm) | AP_MSE_Sum | 0.376 | 28 | -0.023 | 26 | 1.448 | 0.148 | 0.500 |
|  | ML_Amp (mm) | AP_MSE_Sum | -0.304 | 28 | -0.151 | 26 | -0.560 | 0.576 | 0.839 |
|  | ML_PL (mm) | AP_MSE_Sum | 0.008 | 28 | -0.291 | 26 | 1.065 | 0.287 | 0.660 |
|  | ML_RMS (mm) | AP_MSE_Sum | -0.296 | 28 | -0.135 | 26 | -0.586 | 0.558 | 0.819 |
|  | ML_SD (mm) | AP_MSE_Sum | -0.296 | 28 | -0.135 | 26 | -0.586 | 0.558 | 0.819 |
|  | ML_SI (%) | AP_MSE_Sum | 0.359 | 28 | -0.015 | 26 | 1.352 | 0.176 | 0.545 |
|  | ML_Vel_SD (mm/s) | AP_MSE_Sum | -0.266 | 28 | -0.246 | 26 | -0.074 | 0.941 | 0.987 |
|  | MV (mm/s) | AP_MSE_Sum | -0.407 | 28 | -0.368 | 26 | -0.159 | 0.874 | 0.962 |
|  | TPL (mm) | AP_MSE_Sum | -0.254 | 28 | -0.368 | 26 | 0.438 | 0.662 | 0.912 |
|  | AP_Amp (mm) | ML_MSE_Sum | -0.066 | 28 | -0.074 | 26 | 0.028 | 0.978 | 0.990 |
|  | AP_PL (mm) | ML_MSE_Sum | 0.165 | 28 | -0.311 | 26 | 1.690 | 0.091 | 0.481 |
|  | AP_RMS (mm) | ML_MSE_Sum | -0.146 | 28 | -0.063 | 26 | -0.291 | 0.771 | 0.952 |
|  | AP_SD (mm) | ML_MSE_Sum | -0.146 | 28 | -0.063 | 26 | -0.291 | 0.771 | 0.952 |
|  | AP_SI (%) | ML_MSE_Sum | 0.366 | 28 | 0.053 | 26 | 1.145 | 0.252 | 0.613 |
|  | AP_Vel_SD (mm/s) | ML_MSE_Sum | 0.090 | 28 | -0.352 | 26 | 1.585 | 0.113 | 0.492 |
|  | Area_95 (mm²) | ML_MSE_Sum | -0.143 | 28 | -0.123 | 26 | -0.070 | 0.944 | 0.987 |
|  | CHA (mm²) | ML_MSE_Sum | -0.170 | 28 | -0.130 | 26 | -0.142 | 0.887 | 0.962 |
|  | LFS (1/mm) | ML_MSE_Sum | 0.185 | 28 | -0.027 | 26 | 0.741 | 0.459 | 0.761 |
|  | ML_Amp (mm) | ML_MSE_Sum | -0.196 | 28 | -0.105 | 26 | -0.323 | 0.747 | 0.952 |
|  | ML_PL (mm) | ML_MSE_Sum | -0.174 | 28 | -0.253 | 26 | 0.287 | 0.774 | 0.952 |
|  | ML_RMS (mm) | ML_MSE_Sum | -0.069 | 28 | -0.147 | 26 | 0.273 | 0.785 | 0.952 |
|  | ML_SD (mm) | ML_MSE_Sum | -0.069 | 28 | -0.147 | 26 | 0.273 | 0.785 | 0.952 |
|  | ML_SI (%) | ML_MSE_Sum | -0.366 | 28 | -0.053 | 26 | -1.145 | 0.252 | 0.613 |
|  | ML_Vel_SD (mm/s) | ML_MSE_Sum | -0.304 | 28 | -0.256 | 26 | -0.180 | 0.857 | 0.962 |
|  | MV (mm/s) | ML_MSE_Sum | 0.034 | 28 | -0.330 | 26 | 1.304 | 0.192 | 0.560 |
|  | TPL (mm) | ML_MSE_Sum | 0.084 | 28 | -0.330 | 26 | 1.478 | 0.139 | 0.492 |
| DEO | AP_Amp (mm) | AP_MSE_Sum | -0.473 | 28 | 0.112 | 26 | -2.168 | 0.030 | 0.360 |
|  | AP_PL (mm) | AP_MSE_Sum | -0.421 | 28 | 0.344 | 26 | -2.795 | 0.005 | 0.176 |
|  | AP_RMS (mm) | AP_MSE_Sum | -0.471 | 28 | -0.042 | 26 | -1.624 | 0.104 | 0.492 |
|  | AP_SD (mm) | AP_MSE_Sum | -0.471 | 28 | -0.033 | 26 | -1.656 | 0.098 | 0.492 |
|  | AP_SI (%) | AP_MSE_Sum | -0.365 | 28 | 0.044 | 26 | -1.477 | 0.140 | 0.492 |
|  | AP_Vel_SD (mm/s) | AP_MSE_Sum | -0.590 | 28 | 0.267 | 26 | -3.293 | 0.001 | 0.068 |
|  | Area_95 (mm²) | AP_MSE_Sum | -0.543 | 28 | 0.414 | 26 | -3.630 | 0.000 | 0.058 |
|  | CHA (mm²) | AP_MSE_Sum | -0.555 | 28 | 0.166 | 26 | -2.745 | 0.006 | 0.176 |
|  | LFS (1/mm) | AP_MSE_Sum | 0.567 | 28 | -0.318 | 26 | 3.366 | 0.001 | 0.068 |
|  | ML_Amp (mm) | AP_MSE_Sum | -0.389 | 28 | 0.283 | 26 | -2.428 | 0.015 | 0.310 |
|  | ML_PL (mm) | AP_MSE_Sum | -0.097 | 28 | 0.180 | 26 | -0.967 | 0.334 | 0.661 |
|  | ML_RMS (mm) | AP_MSE_Sum | -0.461 | 28 | 0.388 | 26 | -3.143 | 0.002 | 0.068 |
|  | ML_SD (mm) | AP_MSE_Sum | -0.461 | 28 | 0.388 | 26 | -3.143 | 0.002 | 0.068 |
|  | ML_SI (%) | AP_MSE_Sum | 0.365 | 28 | -0.017 | 26 | 1.383 | 0.167 | 0.540 |
|  | ML_Vel_SD (mm/s) | AP_MSE_Sum | -0.382 | 28 | 0.145 | 26 | -1.898 | 0.058 | 0.408 |
|  | MV (mm/s) | AP_MSE_Sum | -0.434 | 28 | 0.248 | 26 | -2.485 | 0.013 | 0.293 |
|  | TPL (mm) | AP_MSE_Sum | -0.332 | 28 | 0.276 | 26 | -2.175 | 0.030 | 0.360 |
|  | AP_Amp (mm) | ML_MSE_Sum | -0.282 | 28 | 0.036 | 26 | -1.128 | 0.259 | 0.623 |
|  | AP_PL (mm) | ML_MSE_Sum | 0.073 | 28 | 0.508 | 26 | -1.685 | 0.092 | 0.481 |
|  | AP_RMS (mm) | ML_MSE_Sum | -0.330 | 28 | 0.101 | 26 | -1.537 | 0.124 | 0.492 |
|  | AP_SD (mm) | ML_MSE_Sum | -0.330 | 28 | 0.107 | 26 | -1.558 | 0.119 | 0.492 |
|  | AP_SI (%) | ML_MSE_Sum | 0.014 | 28 | 0.070 | 26 | -0.194 | 0.846 | 0.962 |
|  | AP_Vel_SD (mm/s) | ML_MSE_Sum | -0.235 | 28 | 0.362 | 26 | -2.141 | 0.032 | 0.360 |
|  | Area_95 (mm²) | ML_MSE_Sum | -0.319 | 28 | 0.334 | 26 | -2.346 | 0.019 | 0.352 |
|  | CHA (mm²) | ML_MSE_Sum | -0.344 | 28 | 0.171 | 26 | -1.839 | 0.066 | 0.408 |
|  | LFS (1/mm) | ML_MSE_Sum | 0.244 | 28 | -0.210 | 26 | 1.600 | 0.110 | 0.492 |
|  | ML_Amp (mm) | ML_MSE_Sum | -0.189 | 28 | 0.257 | 26 | -1.572 | 0.116 | 0.492 |
|  | ML_PL (mm) | ML_MSE_Sum | 0.172 | 28 | 0.260 | 26 | -0.320 | 0.749 | 0.952 |
|  | ML_RMS (mm) | ML_MSE_Sum | -0.179 | 28 | 0.403 | 26 | -2.105 | 0.035 | 0.360 |
|  | ML_SD (mm) | ML_MSE_Sum | -0.179 | 28 | 0.403 | 26 | -2.105 | 0.035 | 0.360 |
|  | ML_SI (%) | ML_MSE_Sum | -0.014 | 28 | -0.065 | 26 | 0.177 | 0.860 | 0.962 |
|  | ML_Vel_SD (mm/s) | ML_MSE_Sum | -0.293 | 28 | 0.156 | 26 | -1.589 | 0.112 | 0.492 |
|  | MV (mm/s) | ML_MSE_Sum | -0.092 | 28 | 0.341 | 26 | -1.549 | 0.121 | 0.492 |
|  | TPL (mm) | ML_MSE_Sum | 0.156 | 28 | 0.352 | 26 | -0.728 | 0.466 | 0.762 |
| LEC | AP_Amp (mm) | AP_MSE_Sum | -0.519 | 28 | -0.287 | 26 | -0.968 | 0.333 | 0.661 |
|  | AP_PL (mm) | AP_MSE_Sum | -0.331 | 28 | -0.046 | 26 | -1.031 | 0.302 | 0.661 |
|  | AP_RMS (mm) | AP_MSE_Sum | -0.459 | 28 | -0.286 | 26 | -0.699 | 0.485 | 0.766 |
|  | AP_SD (mm) | AP_MSE_Sum | -0.459 | 28 | -0.286 | 26 | -0.699 | 0.485 | 0.766 |
|  | AP_SI (%) | AP_MSE_Sum | -0.416 | 28 | -0.213 | 26 | -0.784 | 0.433 | 0.741 |
|  | AP_Vel_SD (mm/s) | AP_MSE_Sum | -0.567 | 28 | -0.054 | 26 | -2.039 | 0.041 | 0.376 |
|  | Area_95 (mm²) | AP_MSE_Sum | -0.470 | 28 | -0.129 | 26 | -1.316 | 0.188 | 0.556 |
|  | CHA (mm²) | AP_MSE_Sum | -0.559 | 28 | -0.089 | 26 | -1.876 | 0.061 | 0.408 |
|  | LFS (1/mm) | AP_MSE_Sum | 0.450 | 28 | 0.271 | 26 | 0.716 | 0.474 | 0.762 |
|  | ML_Amp (mm) | AP_MSE_Sum | -0.507 | 28 | 0.186 | 26 | -2.585 | 0.010 | 0.248 |
|  | ML_PL (mm) | AP_MSE_Sum | -0.149 | 28 | 0.073 | 26 | -0.773 | 0.440 | 0.741 |
|  | ML_RMS (mm) | AP_MSE_Sum | -0.436 | 28 | 0.073 | 26 | -1.870 | 0.061 | 0.408 |
|  | ML_SD (mm) | AP_MSE_Sum | -0.436 | 28 | 0.073 | 26 | -1.870 | 0.061 | 0.408 |
|  | ML_SI (%) | AP_MSE_Sum | 0.416 | 28 | 0.213 | 26 | 0.784 | 0.433 | 0.741 |
|  | ML_Vel_SD (mm/s) | AP_MSE_Sum | -0.191 | 28 | 0.033 | 26 | -0.784 | 0.433 | 0.741 |
|  | MV (mm/s) | AP_MSE_Sum | -0.279 | 28 | 0.004 | 26 | -1.006 | 0.315 | 0.661 |
|  | TPL (mm) | AP_MSE_Sum | -0.317 | 28 | 0.009 | 26 | -1.167 | 0.243 | 0.612 |
|  | AP_Amp (mm) | ML_MSE_Sum | -0.314 | 28 | -0.450 | 26 | 0.553 | 0.580 | 0.840 |
|  | AP_PL (mm) | ML_MSE_Sum | -0.200 | 28 | -0.327 | 26 | 0.473 | 0.636 | 0.889 |
|  | AP_RMS (mm) | ML_MSE_Sum | -0.465 | 28 | -0.543 | 26 | 0.362 | 0.717 | 0.945 |
|  | AP_SD (mm) | ML_MSE_Sum | -0.465 | 28 | -0.543 | 26 | 0.362 | 0.717 | 0.945 |
|  | AP_SI (%) | ML_MSE_Sum | -0.307 | 28 | -0.244 | 26 | -0.236 | 0.813 | 0.952 |
|  | AP_Vel_SD (mm/s) | ML_MSE_Sum | -0.238 | 28 | -0.275 | 26 | 0.137 | 0.891 | 0.962 |
|  | Area_95 (mm²) | ML_MSE_Sum | -0.408 | 28 | -0.428 | 26 | 0.084 | 0.933 | 0.987 |
|  | CHA (mm²) | ML_MSE_Sum | -0.359 | 28 | -0.381 | 26 | 0.088 | 0.930 | 0.987 |
|  | LFS (1/mm) | ML_MSE_Sum | 0.529 | 28 | 0.331 | 26 | 0.847 | 0.397 | 0.732 |
|  | ML_Amp (mm) | ML_MSE_Sum | -0.362 | 28 | -0.014 | 26 | -1.264 | 0.206 | 0.574 |
|  | ML_PL (mm) | ML_MSE_Sum | -0.143 | 28 | -0.208 | 26 | 0.232 | 0.816 | 0.952 |
|  | ML_RMS (mm) | ML_MSE_Sum | -0.343 | 28 | -0.248 | 26 | -0.361 | 0.718 | 0.945 |
|  | ML_SD (mm) | ML_MSE_Sum | -0.343 | 28 | -0.248 | 26 | -0.361 | 0.718 | 0.945 |
|  | ML_SI (%) | ML_MSE_Sum | 0.307 | 28 | 0.244 | 26 | 0.236 | 0.813 | 0.952 |
|  | ML_Vel_SD (mm/s) | ML_MSE_Sum | -0.217 | 28 | -0.228 | 26 | 0.040 | 0.968 | 0.990 |
|  | MV (mm/s) | ML_MSE_Sum | -0.278 | 28 | -0.258 | 26 | -0.075 | 0.941 | 0.987 |
|  | TPL (mm) | ML_MSE_Sum | -0.243 | 28 | -0.254 | 26 | 0.041 | 0.968 | 0.990 |
| LEO | AP_Amp (mm) | AP_MSE_Sum | -0.375 | 28 | -0.367 | 26 | -0.032 | 0.974 | 0.990 |
|  | AP_PL (mm) | AP_MSE_Sum | -0.185 | 28 | -0.257 | 26 | 0.262 | 0.793 | 0.952 |
|  | AP_RMS (mm) | AP_MSE_Sum | -0.501 | 28 | -0.201 | 26 | -1.201 | 0.230 | 0.586 |
|  | AP_SD (mm) | AP_MSE_Sum | -0.501 | 28 | -0.201 | 26 | -1.201 | 0.230 | 0.586 |
|  | AP_SI (%) | AP_MSE_Sum | -0.569 | 28 | -0.268 | 26 | -1.285 | 0.199 | 0.563 |
|  | AP_Vel_SD (mm/s) | AP_MSE_Sum | -0.418 | 28 | -0.381 | 26 | -0.152 | 0.879 | 0.962 |
|  | Area_95 (mm²) | AP_MSE_Sum | -0.375 | 28 | -0.411 | 26 | 0.147 | 0.883 | 0.962 |
|  | CHA (mm²) | AP_MSE_Sum | -0.370 | 28 | -0.432 | 26 | 0.256 | 0.798 | 0.952 |
|  | LFS (1/mm) | AP_MSE_Sum | 0.249 | 28 | 0.387 | 26 | -0.533 | 0.594 | 0.847 |
|  | ML_Amp (mm) | AP_MSE_Sum | -0.146 | 28 | -0.520 | 26 | 1.486 | 0.137 | 0.492 |
|  | ML_PL (mm) | AP_MSE_Sum | -0.085 | 28 | -0.125 | 26 | 0.140 | 0.889 | 0.962 |
|  | ML_RMS (mm) | AP_MSE_Sum | -0.103 | 28 | -0.489 | 26 | 1.493 | 0.135 | 0.492 |
|  | ML_SD (mm) | AP_MSE_Sum | -0.103 | 28 | -0.489 | 26 | 1.493 | 0.135 | 0.492 |
|  | ML_SI (%) | AP_MSE_Sum | 0.569 | 28 | 0.268 | 26 | 1.285 | 0.199 | 0.563 |
|  | ML_Vel_SD (mm/s) | AP_MSE_Sum | -0.202 | 28 | -0.190 | 26 | -0.043 | 0.966 | 0.990 |
|  | MV (mm/s) | AP_MSE_Sum | -0.263 | 28 | -0.181 | 26 | -0.299 | 0.765 | 0.952 |
|  | TPL (mm) | AP_MSE_Sum | -0.151 | 28 | -0.181 | 26 | 0.107 | 0.915 | 0.982 |
|  | AP_Amp (mm) | ML_MSE_Sum | -0.530 | 28 | -0.312 | 26 | -0.925 | 0.355 | 0.689 |
|  | AP_PL (mm) | ML_MSE_Sum | -0.502 | 28 | -0.159 | 26 | -1.355 | 0.175 | 0.545 |
|  | AP_RMS (mm) | ML_MSE_Sum | -0.375 | 28 | -0.204 | 26 | -0.648 | 0.517 | 0.781 |
|  | AP_SD (mm) | ML_MSE_Sum | -0.375 | 28 | -0.204 | 26 | -0.648 | 0.517 | 0.781 |
|  | AP_SI (%) | ML_MSE_Sum | -0.205 | 28 | -0.212 | 26 | 0.025 | 0.980 | 0.990 |
|  | AP_Vel_SD (mm/s) | ML_MSE_Sum | -0.550 | 28 | -0.296 | 26 | -1.084 | 0.278 | 0.653 |
|  | Area_95 (mm²) | ML_MSE_Sum | -0.527 | 28 | -0.294 | 26 | -0.980 | 0.327 | 0.661 |
|  | CHA (mm²) | ML_MSE_Sum | -0.554 | 28 | -0.376 | 26 | -0.792 | 0.429 | 0.741 |
|  | LFS (1/mm) | ML_MSE_Sum | 0.104 | 28 | 0.329 | 26 | -0.821 | 0.411 | 0.737 |
|  | ML_Amp (mm) | ML_MSE_Sum | -0.307 | 28 | -0.438 | 26 | 0.528 | 0.598 | 0.847 |
|  | ML_PL (mm) | ML_MSE_Sum | -0.493 | 28 | -0.018 | 26 | -1.807 | 0.071 | 0.417 |
|  | ML_RMS (mm) | ML_MSE_Sum | -0.318 | 28 | -0.318 | 26 | 0.000 | 1.000 | 1.000 |
|  | ML_SD (mm) | ML_MSE_Sum | -0.318 | 28 | -0.318 | 26 | 0.000 | 1.000 | 1.000 |
|  | ML_SI (%) | ML_MSE_Sum | 0.205 | 28 | 0.212 | 26 | -0.025 | 0.980 | 0.990 |
|  | ML_Vel_SD (mm/s) | ML_MSE_Sum | -0.595 | 28 | -0.063 | 26 | -2.154 | 0.031 | 0.360 |
|  | MV (mm/s) | ML_MSE_Sum | -0.537 | 28 | -0.061 | 26 | -1.865 | 0.062 | 0.408 |
|  | TPL (mm) | ML_MSE_Sum | -0.524 | 28 | -0.061 | 26 | -1.802 | 0.071 | 0.417 |
| REC | AP_Amp (mm) | AP_MSE_Sum | -0.358 | 28 | -0.090 | 26 | -0.984 | 0.325 | 0.661 |
|  | AP_PL (mm) | AP_MSE_Sum | -0.335 | 28 | -0.104 | 26 | -0.845 | 0.398 | 0.732 |
|  | AP_RMS (mm) | AP_MSE_Sum | -0.351 | 28 | -0.272 | 26 | -0.303 | 0.762 | 0.952 |
|  | AP_SD (mm) | AP_MSE_Sum | -0.351 | 28 | -0.272 | 26 | -0.303 | 0.762 | 0.952 |
|  | AP_SI (%) | AP_MSE_Sum | -0.150 | 28 | 0.084 | 26 | -0.815 | 0.415 | 0.737 |
|  | AP_Vel_SD (mm/s) | AP_MSE_Sum | -0.388 | 28 | -0.056 | 26 | -1.223 | 0.221 | 0.579 |
|  | Area_95 (mm²) | AP_MSE_Sum | -0.345 | 28 | -0.158 | 26 | -0.694 | 0.488 | 0.766 |
|  | CHA (mm²) | AP_MSE_Sum | -0.394 | 28 | -0.083 | 26 | -1.154 | 0.249 | 0.613 |
|  | LFS (1/mm) | AP_MSE_Sum | 0.355 | 28 | 0.066 | 26 | 1.056 | 0.291 | 0.660 |
|  | ML_Amp (mm) | AP_MSE_Sum | -0.396 | 28 | 0.115 | 26 | -1.850 | 0.064 | 0.408 |
|  | ML_PL (mm) | AP_MSE_Sum | -0.347 | 28 | -0.164 | 26 | -0.680 | 0.496 | 0.767 |
|  | ML_RMS (mm) | AP_MSE_Sum | -0.346 | 28 | -0.061 | 26 | -1.038 | 0.299 | 0.661 |
|  | ML_SD (mm) | AP_MSE_Sum | -0.346 | 28 | -0.061 | 26 | -1.038 | 0.299 | 0.661 |
|  | ML_SI (%) | AP_MSE_Sum | 0.150 | 28 | -0.084 | 26 | 0.815 | 0.415 | 0.737 |
|  | ML_Vel_SD (mm/s) | AP_MSE_Sum | -0.407 | 28 | -0.149 | 26 | -0.976 | 0.329 | 0.661 |
|  | MV (mm/s) | AP_MSE_Sum | -0.364 | 28 | -0.182 | 26 | -0.683 | 0.494 | 0.767 |
|  | TPL (mm) | AP_MSE_Sum | -0.358 | 28 | -0.182 | 26 | -0.659 | 0.510 | 0.781 |
|  | AP_Amp (mm) | ML_MSE_Sum | -0.194 | 28 | -0.092 | 26 | -0.361 | 0.718 | 0.945 |
|  | AP_PL (mm) | ML_MSE_Sum | -0.216 | 28 | -0.157 | 26 | -0.212 | 0.832 | 0.954 |
|  | AP_RMS (mm) | ML_MSE_Sum | -0.216 | 28 | -0.381 | 26 | 0.629 | 0.529 | 0.788 |
|  | AP_SD (mm) | ML_MSE_Sum | -0.216 | 28 | -0.381 | 26 | 0.629 | 0.529 | 0.788 |
|  | AP_SI (%) | ML_MSE_Sum | 0.245 | 28 | 0.004 | 26 | 0.852 | 0.394 | 0.732 |
|  | AP_Vel_SD (mm/s) | ML_MSE_Sum | -0.325 | 28 | -0.058 | 26 | -0.966 | 0.334 | 0.661 |
|  | Area_95 (mm²) | ML_MSE_Sum | -0.365 | 28 | -0.135 | 26 | -0.854 | 0.393 | 0.732 |
|  | CHA (mm²) | ML_MSE_Sum | -0.414 | 28 | -0.059 | 26 | -1.320 | 0.187 | 0.556 |
|  | LFS (1/mm) | ML_MSE_Sum | 0.344 | 28 | 0.041 | 26 | 1.099 | 0.272 | 0.644 |
|  | ML_Amp (mm) | ML_MSE_Sum | -0.437 | 28 | 0.151 | 26 | -2.148 | 0.032 | 0.360 |
|  | ML_PL (mm) | ML_MSE_Sum | -0.433 | 28 | -0.223 | 26 | -0.819 | 0.413 | 0.737 |
|  | ML_RMS (mm) | ML_MSE_Sum | -0.491 | 28 | 0.039 | 26 | -1.995 | 0.046 | 0.376 |
|  | ML_SD (mm) | ML_MSE_Sum | -0.491 | 28 | 0.039 | 26 | -1.995 | 0.046 | 0.376 |
|  | ML_SI (%) | ML_MSE_Sum | -0.245 | 28 | -0.004 | 26 | -0.852 | 0.394 | 0.732 |
|  | ML_Vel_SD (mm/s) | ML_MSE_Sum | -0.627 | 28 | -0.226 | 26 | -1.753 | 0.080 | 0.439 |
|  | MV (mm/s) | ML_MSE_Sum | -0.431 | 28 | -0.194 | 26 | -0.916 | 0.360 | 0.692 |
|  | TPL (mm) | ML_MSE_Sum | -0.322 | 28 | -0.194 | 26 | -0.476 | 0.634 | 0.889 |
| REO | AP_Amp (mm) | AP_MSE_Sum | -0.116 | 28 | -0.018 | 26 | -0.341 | 0.733 | 0.952 |
|  | AP_PL (mm) | AP_MSE_Sum | -0.420 | 28 | 0.140 | 26 | -2.037 | 0.042 | 0.376 |
|  | AP_RMS (mm) | AP_MSE_Sum | -0.014 | 28 | -0.136 | 26 | 0.425 | 0.671 | 0.912 |
|  | AP_SD (mm) | AP_MSE_Sum | -0.014 | 28 | -0.136 | 26 | 0.425 | 0.671 | 0.912 |
|  | AP_SI (%) | AP_MSE_Sum | -0.117 | 28 | 0.235 | 26 | -1.236 | 0.217 | 0.574 |
|  | AP_Vel_SD (mm/s) | AP_MSE_Sum | -0.493 | 28 | 0.108 | 26 | -2.244 | 0.025 | 0.360 |
|  | Area_95 (mm²) | AP_MSE_Sum | -0.115 | 28 | -0.157 | 26 | 0.148 | 0.882 | 0.962 |
|  | CHA (mm²) | AP_MSE_Sum | -0.240 | 28 | -0.022 | 26 | -0.771 | 0.441 | 0.741 |
|  | LFS (1/mm) | AP_MSE_Sum | -0.026 | 28 | 0.273 | 26 | -1.059 | 0.289 | 0.660 |
|  | ML_Amp (mm) | AP_MSE_Sum | -0.362 | 28 | -0.087 | 26 | -1.011 | 0.312 | 0.661 |
|  | ML_PL (mm) | AP_MSE_Sum | -0.436 | 28 | -0.082 | 26 | -1.333 | 0.183 | 0.556 |
|  | ML_RMS (mm) | AP_MSE_Sum | -0.204 | 28 | -0.144 | 26 | -0.214 | 0.830 | 0.954 |
|  | ML_SD (mm) | AP_MSE_Sum | -0.204 | 28 | -0.144 | 26 | -0.214 | 0.830 | 0.954 |
|  | ML_SI (%) | AP_MSE_Sum | 0.117 | 28 | -0.235 | 26 | 1.236 | 0.217 | 0.574 |
|  | ML_Vel_SD (mm/s) | AP_MSE_Sum | -0.462 | 28 | -0.065 | 26 | -1.505 | 0.132 | 0.492 |
|  | MV (mm/s) | AP_MSE_Sum | -0.473 | 28 | -0.003 | 26 | -1.768 | 0.077 | 0.436 |
|  | TPL (mm) | AP_MSE_Sum | -0.426 | 28 | -0.003 | 26 | -1.564 | 0.118 | 0.492 |
|  | AP_Amp (mm) | ML_MSE_Sum | -0.019 | 28 | 0.070 | 26 | -0.308 | 0.758 | 0.952 |
|  | AP_PL (mm) | ML_MSE_Sum | -0.349 | 28 | 0.242 | 26 | -2.115 | 0.034 | 0.360 |
|  | AP_RMS (mm) | ML_MSE_Sum | -0.002 | 28 | -0.076 | 26 | 0.257 | 0.797 | 0.952 |
|  | AP_SD (mm) | ML_MSE_Sum | -0.002 | 28 | -0.076 | 26 | 0.257 | 0.797 | 0.952 |
|  | AP_SI (%) | ML_MSE_Sum | 0.046 | 28 | 0.442 | 26 | -1.484 | 0.138 | 0.492 |
|  | AP_Vel_SD (mm/s) | ML_MSE_Sum | -0.311 | 28 | 0.250 | 26 | -1.997 | 0.046 | 0.376 |
|  | Area_95 (mm²) | ML_MSE_Sum | -0.074 | 28 | -0.199 | 26 | 0.441 | 0.659 | 0.912 |
|  | CHA (mm²) | ML_MSE_Sum | -0.213 | 28 | -0.062 | 26 | -0.534 | 0.593 | 0.847 |
|  | LFS (1/mm) | ML_MSE_Sum | -0.136 | 28 | 0.400 | 26 | -1.940 | 0.052 | 0.408 |
|  | ML_Amp (mm) | ML_MSE_Sum | -0.237 | 28 | -0.302 | 26 | 0.243 | 0.808 | 0.952 |
|  | ML_PL (mm) | ML_MSE_Sum | -0.365 | 28 | -0.099 | 26 | -0.981 | 0.327 | 0.661 |
|  | ML_RMS (mm) | ML_MSE_Sum | -0.146 | 28 | -0.341 | 26 | 0.721 | 0.471 | 0.762 |
|  | ML_SD (mm) | ML_MSE_Sum | -0.146 | 28 | -0.341 | 26 | 0.721 | 0.471 | 0.762 |
|  | ML_SI (%) | ML_MSE_Sum | -0.046 | 28 | -0.442 | 26 | 1.484 | 0.138 | 0.492 |
|  | ML_Vel_SD (mm/s) | ML_MSE_Sum | -0.505 | 28 | -0.099 | 26 | -1.581 | 0.114 | 0.492 |
|  | MV (mm/s) | ML_MSE_Sum | -0.364 | 28 | 0.035 | 26 | -1.442 | 0.149 | 0.500 |
|  | TPL (mm) | ML_MSE_Sum | -0.357 | 28 | 0.035 | 26 | -1.414 | 0.157 | 0.518 |

Abbreviations: Total Path Length(TPL), Antero-posterior Path Length(AP_PL), Medio-lateral Path Length(ML_PL), Antero-posterior Displacement Amplitude(AP_Amp), Medio-lateral Displacement Amplitude(ML_Amp), Antero-posterior Sway Index(AP_SI), Medio-lateral Sway Index(ML_SI), Antero-posterior Displacement Standard Deviation(AP_SD), Medio-lateral Displacement Standard Deviation(ML_SD), Total Mean Velocity(MV), Antero-posterior Velocity Standard Deviation(AP_Vel_SD), Medio-lateral Velocity Standard Deviation(ML_Vel_SD), Antero-posterior Root-Mean-Square Displacement(AP_RMS), Medio-lateral Root-Mean-Square Displacement(ML_RMS), 95% Confidence Ellipse Area(Area_95), Convex Hull Area(CHA). double-leg stance with eyes open (DEO), double-leg stance with eyes closed (DEC), right-leg stance with eyes open (REO); 4) right-leg stance with eyes closed (REC), left-leg stance with eyes open (LEO), left-leg stance with eyes closed (LEC). *, P<0.05.

Appendix 5 Intraclass correlation coefficients (ICC) for COP postural sway parameters.

| Condition | Metric | N_pairs | ICC(2,1) |
| --- | --- | --- | --- |
| DEO | TPL (mm) | 41 | 0.439 |
|  | AP_PL (mm) | 41 | 0.466 |
|  | ML_PL (mm) | 41 | 0.41 |
|  | MV (mm/s) | 41 | 0.426 |
|  | AP_Vel_SD (mm/s) | 41 | 0.241 |
|  | ML_Vel_SD (mm/s) | 41 | 0.097 |
|  | AP_Amp (mm) | 41 | 0.497 |
|  | ML_Amp (mm) | 41 | 0.157 |
|  | AP_SD (mm) | 41 | 0.676 |
|  | ML_SD (mm) | 41 | 0.158 |
|  | AP_SI (%) | 41 | 0.442 |
|  | ML_SI (%) | 41 | 0.442 |
|  | AP_RMS (mm) | 41 | 0.676 |
|  | ML_RMS (mm) | 41 | 0.158 |
|  | Area_95 (mm²) | 41 | 0.377 |
|  | CHA (mm²) | 41 | 0.214 |
|  | LFS (1/mm) | 41 | 0.424 |
| DEC | TPL (mm) | 44 | 0.815 |
|  | AP_PL (mm) | 44 | 0.802 |
|  | ML_PL (mm) | 44 | 0.796 |
|  | MV (mm/s) | 44 | 0.797 |
|  | AP_Vel_SD (mm/s) | 44 | 0.697 |
|  | ML_Vel_SD (mm/s) | 44 | 0.684 |
|  | AP_Amp (mm) | 44 | 0.543 |
|  | ML_Amp (mm) | 44 | 0.529 |
|  | AP_SD (mm) | 44 | 0.608 |
|  | ML_SD (mm) | 44 | 0.576 |
|  | AP_SI (%) | 44 | 0.743 |
|  | ML_SI (%) | 44 | 0.743 |
|  | AP_RMS (mm) | 44 | 0.608 |
|  | ML_RMS (mm) | 44 | 0.576 |
|  | Area_95 (mm²) | 44 | 0.688 |
|  | CHA (mm²) | 44 | 0.654 |
|  | LFS (1/mm) | 44 | 0.528 |
| LEO | TPL (mm) | 52 | 0.785 |
|  | AP_PL (mm) | 52 | 0.839 |
|  | ML_PL (mm) | 52 | 0.674 |
|  | MV (mm/s) | 52 | 0.778 |
|  | AP_Vel_SD (mm/s) | 52 | 0.568 |
|  | ML_Vel_SD (mm/s) | 52 | 0.636 |
|  | AP_Amp (mm) | 52 | 0.265 |
|  | ML_Amp (mm) | 52 | 0.511 |
|  | AP_SD (mm) | 52 | 0.515 |
|  | ML_SD (mm) | 52 | 0.58 |
|  | AP_SI (%) | 52 | 0.684 |
|  | ML_SI (%) | 52 | 0.684 |
|  | AP_RMS (mm) | 52 | 0.515 |
|  | ML_RMS (mm) | 52 | 0.58 |
|  | Area_95 (mm²) | 52 | 0.594 |
|  | CHA (mm²) | 52 | 0.433 |
|  | LFS (1/mm) | 52 | 0.523 |
| LEC | TPL (mm) | 52 | 0.908 |
|  | AP_PL (mm) | 52 | 0.898 |
|  | ML_PL (mm) | 52 | 0.884 |
|  | MV (mm/s) | 52 | 0.907 |
|  | AP_Vel_SD (mm/s) | 52 | 0.753 |
|  | ML_Vel_SD (mm/s) | 52 | 0.85 |
|  | AP_Amp (mm) | 52 | 0.688 |
|  | ML_Amp (mm) | 52 | 0.791 |
|  | AP_SD (mm) | 52 | 0.782 |
|  | ML_SD (mm) | 52 | 0.862 |
|  | AP_SI (%) | 52 | 0.692 |
|  | ML_SI (%) | 52 | 0.692 |
|  | AP_RMS (mm) | 52 | 0.782 |
|  | ML_RMS (mm) | 52 | 0.862 |
|  | Area_95 (mm²) | 52 | 0.848 |
|  | CHA (mm²) | 52 | 0.864 |
|  | LFS (1/mm) | 52 | 0.746 |
| REO | TPL (mm) | 53 | 0.753 |
|  | AP_PL (mm) | 53 | 0.769 |
|  | ML_PL (mm) | 53 | 0.744 |
|  | MV (mm/s) | 53 | 0.742 |
|  | AP_Vel_SD (mm/s) | 53 | 0.714 |
|  | ML_Vel_SD (mm/s) | 53 | 0.699 |
|  | AP_Amp (mm) | 53 | 0.297 |
|  | ML_Amp (mm) | 53 | 0.579 |
|  | AP_SD (mm) | 53 | 0.421 |
|  | ML_SD (mm) | 53 | 0.582 |
|  | AP_SI (%) | 53 | 0.816 |
|  | ML_SI (%) | 53 | 0.816 |
|  | AP_RMS (mm) | 53 | 0.421 |
|  | ML_RMS (mm) | 53 | 0.582 |
|  | Area_95 (mm²) | 53 | 0.369 |
|  | CHA (mm²) | 53 | 0.337 |
|  | LFS (1/mm) | 53 | 0.438 |
| REC | TPL (mm) | 50 | 0.694 |
|  | AP_PL (mm) | 50 | 0.64 |
|  | ML_PL (mm) | 50 | 0.771 |
|  | MV (mm/s) | 50 | 0.71 |
|  | AP_Vel_SD (mm/s) | 50 | 0.304 |
|  | ML_Vel_SD (mm/s) | 50 | 0.19 |
|  | AP_Amp (mm) | 50 | 0.345 |
|  | ML_Amp (mm) | 50 | 0.409 |
|  | AP_SD (mm) | 50 | 0.494 |
|  | ML_SD (mm) | 50 | 0.335 |
|  | AP_SI (%) | 50 | 0.636 |
|  | ML_SI (%) | 50 | 0.636 |
|  | AP_RMS (mm) | 50 | 0.494 |
|  | ML_RMS (mm) | 50 | 0.335 |
|  | Area_95 (mm²) | 50 | 0.287 |
|  | CHA (mm²) | 50 | 0.127 |
|  | LFS (1/mm) | 50 | 0.627 |

Abbreviations: Total Path Length(TPL), Antero-posterior Path Length(AP_PL), Medio-lateral Path Length(ML_PL), Antero-posterior Displacement Amplitude(AP_Amp), Medio-lateral Displacement Amplitude(ML_Amp), Antero-posterior Sway Index(AP_SI), Medio-lateral Sway Index(ML_SI), Antero-posterior Displacement Standard Deviation(AP_SD), Medio-lateral Displacement Standard Deviation(ML_SD), Total Mean Velocity(MV), Antero-posterior Velocity Standard Deviation(AP_Vel_SD), Medio-lateral Velocity Standard Deviation(ML_Vel_SD), Antero-posterior Root-Mean-Square Displacement(AP_RMS), Medio-lateral Root-Mean-Square Displacement(ML_RMS), 95% Confidence Ellipse Area(Area_95), Convex Hull Area(CHA). double-leg stance with eyes open (DEO), double-leg stance with eyes closed (DEC), right-leg stance with eyes open (REO); 4) right-leg stance with eyes closed (REC), left-leg stance with eyes open (LEO), left-leg stance with eyes closed (LEC). *, P<0.05. N_pairs is notably lower for the DEO condition (N = 41) because trial-level exports for the third trial were unavailable for a subset of participants in this condition within the reliability-analysis dataset, despite complete averaged values being available for all 54 participants in the primary analysis.

Appendix 6 Intraclass correlation coefficients (ICC) for MSE complexity metrics.

| Condition | Metric | N_pairs | ICC(2,1) |
| --- | --- | --- | --- |
| DEO | AP_MSE_Sum | 43 | 0.572 |
|  | ML_MSE_Sum | 43 | 0.262 |
|  | AP_MSE_Scale1 | 43 | 0.373 |
|  | AP_MSE_Scale2 | 43 | 0.573 |
|  | AP_MSE_Scale3 | 43 | 0.594 |
|  | AP_MSE_Scale4 | 43 | 0.619 |
|  | AP_MSE_Scale5 | 43 | 0.592 |
|  | AP_MSE_Scale6 | 43 | 0.516 |
|  | AP_MSE_Scale7 | 43 | 0.541 |
|  | AP_MSE_Scale8 | 43 | 0.436 |
|  | ML_MSE_Scale1 | 43 | 0.275 |
|  | ML_MSE_Scale2 | 43 | 0.358 |
|  | ML_MSE_Scale3 | 43 | 0.322 |
|  | ML_MSE_Scale4 | 43 | 0.275 |
|  | ML_MSE_Scale5 | 43 | 0.144 |
|  | ML_MSE_Scale6 | 43 | 0.073 |
|  | ML_MSE_Scale7 | 43 | 0.186 |
|  | ML_MSE_Scale8 | 43 | 0.234 |
| DEC | AP_MSE_Sum | 44 | 0.567 |
|  | ML_MSE_Sum | 44 | 0.482 |
|  | AP_MSE_Scale1 | 44 | 0.397 |
|  | AP_MSE_Scale2 | 44 | 0.717 |
|  | AP_MSE_Scale3 | 44 | 0.668 |
|  | AP_MSE_Scale4 | 44 | 0.604 |
|  | AP_MSE_Scale5 | 44 | 0.588 |
|  | AP_MSE_Scale6 | 44 | 0.371 |
|  | AP_MSE_Scale7 | 44 | 0.429 |
|  | AP_MSE_Scale8 | 44 | 0.249 |
|  | ML_MSE_Scale1 | 44 | 0.225 |
|  | ML_MSE_Scale2 | 44 | 0.616 |
|  | ML_MSE_Scale3 | 44 | 0.571 |
|  | ML_MSE_Scale4 | 44 | 0.576 |
|  | ML_MSE_Scale5 | 44 | 0.243 |
|  | ML_MSE_Scale6 | 44 | 0.256 |
|  | ML_MSE_Scale7 | 44 | 0.069 |
|  | ML_MSE_Scale8 | 44 | 0.425 |
| LEO | AP_MSE_Sum | 54 | 0.914 |
|  | ML_MSE_Sum | 54 | 0.908 |
|  | AP_MSE_Scale1 | 54 | 0.662 |
|  | AP_MSE_Scale2 | 54 | 0.904 |
|  | AP_MSE_Scale3 | 54 | 0.93 |
|  | AP_MSE_Scale4 | 54 | 0.929 |
|  | AP_MSE_Scale5 | 54 | 0.895 |
|  | AP_MSE_Scale6 | 54 | 0.888 |
|  | AP_MSE_Scale7 | 54 | 0.869 |
|  | AP_MSE_Scale8 | 54 | 0.713 |
|  | ML_MSE_Scale1 | 54 | 0.721 |
|  | ML_MSE_Scale2 | 54 | 0.917 |
|  | ML_MSE_Scale3 | 54 | 0.939 |
|  | ML_MSE_Scale4 | 54 | 0.904 |
|  | ML_MSE_Scale5 | 54 | 0.875 |
|  | ML_MSE_Scale6 | 54 | 0.85 |
|  | ML_MSE_Scale7 | 54 | 0.765 |
|  | ML_MSE_Scale8 | 54 | 0.64 |
| LEC | AP_MSE_Sum | 53 | 0.725 |
|  | ML_MSE_Sum | 53 | 0.723 |
|  | AP_MSE_Scale1 | 53 | 0.541 |
|  | AP_MSE_Scale2 | 53 | 0.761 |
|  | AP_MSE_Scale3 | 53 | 0.774 |
|  | AP_MSE_Scale4 | 53 | 0.739 |
|  | AP_MSE_Scale5 | 53 | 0.68 |
|  | AP_MSE_Scale6 | 53 | 0.695 |
|  | AP_MSE_Scale7 | 53 | 0.615 |
|  | AP_MSE_Scale8 | 53 | 0.618 |
|  | ML_MSE_Scale1 | 53 | 0.909 |
|  | ML_MSE_Scale2 | 53 | 0.88 |
|  | ML_MSE_Scale3 | 53 | 0.853 |
|  | ML_MSE_Scale4 | 53 | 0.804 |
|  | ML_MSE_Scale5 | 53 | 0.663 |
|  | ML_MSE_Scale6 | 53 | 0.593 |
|  | ML_MSE_Scale7 | 53 | 0.53 |
|  | ML_MSE_Scale8 | 53 | 0.322 |
| REO | AP_MSE_Sum | 53 | 0.857 |
|  | ML_MSE_Sum | 53 | 0.885 |
|  | AP_MSE_Scale1 | 53 | 0.579 |
|  | AP_MSE_Scale2 | 53 | 0.818 |
|  | AP_MSE_Scale3 | 53 | 0.872 |
|  | AP_MSE_Scale4 | 53 | 0.884 |
|  | AP_MSE_Scale5 | 53 | 0.843 |
|  | AP_MSE_Scale6 | 53 | 0.821 |
|  | AP_MSE_Scale7 | 53 | 0.786 |
|  | AP_MSE_Scale8 | 53 | 0.717 |
|  | ML_MSE_Scale1 | 53 | 0.87 |
|  | ML_MSE_Scale2 | 53 | 0.915 |
|  | ML_MSE_Scale3 | 53 | 0.923 |
|  | ML_MSE_Scale4 | 53 | 0.895 |
|  | ML_MSE_Scale5 | 53 | 0.881 |
|  | ML_MSE_Scale6 | 53 | 0.805 |
|  | ML_MSE_Scale7 | 53 | 0.752 |
|  | ML_MSE_Scale8 | 53 | 0.582 |
| REC | AP_MSE_Sum | 52 | 0.792 |
|  | ML_MSE_Sum | 52 | 0.81 |
|  | AP_MSE_Scale1 | 52 | 0.64 |
|  | AP_MSE_Scale2 | 52 | 0.9 |
|  | AP_MSE_Scale3 | 52 | 0.885 |
|  | AP_MSE_Scale4 | 52 | 0.828 |
|  | AP_MSE_Scale5 | 52 | 0.737 |
|  | AP_MSE_Scale6 | 52 | 0.736 |
|  | AP_MSE_Scale7 | 52 | 0.615 |
|  | AP_MSE_Scale8 | 52 | 0.558 |
|  | ML_MSE_Scale1 | 52 | 0.523 |
|  | ML_MSE_Scale2 | 52 | 0.798 |
|  | ML_MSE_Scale3 | 52 | 0.862 |
|  | ML_MSE_Scale4 | 52 | 0.83 |
|  | ML_MSE_Scale5 | 52 | 0.786 |
|  | ML_MSE_Scale6 | 52 | 0.667 |
|  | ML_MSE_Scale7 | 52 | 0.643 |
|  | ML_MSE_Scale8 | 52 | 0.587 |

Abbreviations: Total Path Length(TPL), Antero-posterior Path Length(AP_PL), Medio-lateral Path Length(ML_PL), Antero-posterior Displacement Amplitude(AP_Amp), Medio-lateral Displacement Amplitude(ML_Amp), Antero-posterior Sway Index(AP_SI), Medio-lateral Sway Index(ML_SI), Antero-posterior Displacement Standard Deviation(AP_SD), Medio-lateral Displacement Standard Deviation(ML_SD), Total Mean Velocity(MV), Antero-posterior Velocity Standard Deviation(AP_Vel_SD), Medio-lateral Velocity Standard Deviation(ML_Vel_SD), Antero-posterior Root-Mean-Square Displacement(AP_RMS), Medio-lateral Root-Mean-Square Displacement(ML_RMS), 95% Confidence Ellipse Area(Area_95), Convex Hull Area(CHA). double-leg stance with eyes open (DEO), double-leg stance with eyes closed (DEC), right-leg stance with eyes open (REO); 4) right-leg stance with eyes closed (REC), left-leg stance with eyes open (LEO), left-leg stance with eyes closed (LEC). *, P<0.05. N_pairs is notably lower for the DEO condition (N = 43) because trial-level exports for the third trial were unavailable for a subset of participants in this condition within the reliability-analysis dataset, despite complete averaged values being available for all 54 participants in the primary analysis.
